# Cancer molecular subtyping using limited multi-omics data with missingness

**DOI:** 10.1371/journal.pcbi.1012710

**Published:** 2024-12-26

**Authors:** Yongqi Bu, Jiaxuan Liang, Zhen Li, Jianbo Wang, Jun Wang, Guoxian Yu

**Affiliations:** 1 School of Software, Shandong University, Jinan, Shandong, China; 2 Joint SDU-NTU Centre for Artificial Intelligence Research, Shandong University, Jinan, Shandong, China; 3 Department of Gastroenterology, Qilu Hospital of Shandong University, Jinan, Shandong, China; 4 Department of Radiation Oncology, Qilu Hospital of Shandong University, Jinan, Shandong, China; University College London, UNITED KINGDOM OF GREAT BRITAIN AND NORTHERN IRELAND

## Abstract

Diagnosing cancer subtypes is a prerequisite for precise treatment. Existing multi-omics data fusion-based diagnostic solutions build on the requisite of sufficient samples with complete multi-omics data, which is challenging to obtain in clinical applications. To address the bottleneck of collecting sufficient samples with complete data in clinical applications, we proposed a flexible integrative model (CancerSD) to diagnose cancer subtype using limited samples with incomplete multi-omics data. CancerSD designs contrastive learning tasks and masking-and-reconstruction tasks to reliably impute missing omics, and fuses available omics data with the imputed ones to accurately diagnose cancer subtypes. To address the issue of limited clinical samples, it introduces a category-level contrastive loss to extend the meta-learning framework, effectively transferring knowledge from external datasets to pretrain the diagnostic model. Experiments on benchmark datasets show that CancerSD not only gives accurate diagnosis, but also maintains a high authenticity and good interpretability. In addition, CancerSD identifies important molecular characteristics associated with cancer subtypes, and it defines the Integrated CancerSD Score that can serve as an independent predictive factor for patient prognosis.

## Author summary

With the continuous development of high-throughput life science measurement technologies, various omics data for disease have been rapidly accumulating, driving the application of advanced artificial intelligence technologies in cancer-related research. These massive data generated by high-throughput technologies are collectively referred to as multi-omics data, which include genomics, transcriptomics, and epigenomics, among others. The integration of such multi-omics data provides researchers with a foundation to decode the intricate mechanisms of biological systems. However, the high heterogeneity among these data hinders effective integration. Furthermore, due to factors such as high costs, technical challenges, and patient opposition, collecting sufficient samples with complete data in clinical scenarios is highly challenging, which further complicates the integration process. In light of these challenges, we developed a new integration framework called CancerSD to utilize limited multi-omics data with missingness for accurate and flexible cancer subtype diagnosis. Our results demonstrate that CancerSD outperforms existing multi-omics integration algorithms and exhibits good interpretability and application value.

## Introduction

Cancer, a complex disease stemming from diverse origins, stands as the leading cause of premature death worldwide, significantly impeding further extension of life expectancy [1]. Cancer is generally regarded as a cellular disease [2], where genetic mutations, epigenetic changes, cellular biological backgrounds, individual patient-specific characteristics, and environmental influences may all contribute to its initiation and proliferation [3]. The high heterogeneity and complex molecular mechanisms inherent in cancer underscore the subdivision of each single-tissue cancer type into multiple molecular subtypes [4]. Patients with different subtypes typically manifest distinct clinical phenotypes, therapeutic strategy, and prognoses [5–7]. Therefore, accurate subtype diagnosis holds immense potential for propelling advancements in personalized medicine treatments, reducing mortality rates, and prolonging patient survival. However, clinical diagnosis of these molecular subtypes is often costly, time-consuming, and reliant on specialized expertise. Given that, the imperative arises to develop accurate and trustworthy computational solutions for cancer subtype diagnosis.

Early researches [8–10] primarily depended on single-omics data to identify cancer subtypes and proved the feasibility of computationally diagnosing subtypes. With the rapid development of high-throughput sequencing, a wealth of multi-omics data has emerged, providing a comprehensive insight into organisms and revealing intricate mechanistic underpinnings of biological systems from diverse perspectives [11]. Consequently, more recent developments [12–14] have shifted their focus towards integrating multiple omics data. However, these methods canonically assume the availability of abundant well-annotated cancer samples characterized by completely-paired multi-omics data. Unfortunately, collecting such samples is challenging, predominantly due to limitations in inspection equipment, high testing costs, and considerations of legal and ethical aspects. In practice, only limited samples with incomplete multi-omics data are available, which significantly limits the applicability and effectiveness of the aforementioned methods.

One naive solution for incomplete multi-omics data is to remove corresponding samples directly [14–16], which often results in the loss of valuable samples [17]. To mitigate such information loss, various strategies [18–21] have been explored to recover the missing omics. These methods mostly involve meticulously designed imputation processes, and some even apply distinct treatments for different scenarios, thus imposing specific requirements on the quantity of training data. However, the acquisition of well-annotated samples remains a formidable challenge in biomedical domains [22]. In such cases, it is challenging to train an accurate and reliable diagnostic model using limited samples from the in-house dataset.

To address the challenge of limited training data, few-shot (or few-sample) learning has become a prevalent paradigm in the biomedical field. A typical solution is to leverage external datasets with abundant and relevant samples to support the optimization of the model applied to the target dataset [23–25]. However, these existing methods may overlook disparities among different datasets (i.e., sample distributions), which give rise to negative transfer [26]. In such cases, abundant samples from external datasets fail to further improve the model for downstream tasks on the target dataset but even make the optimization process more challenging.

**Fig 1:**
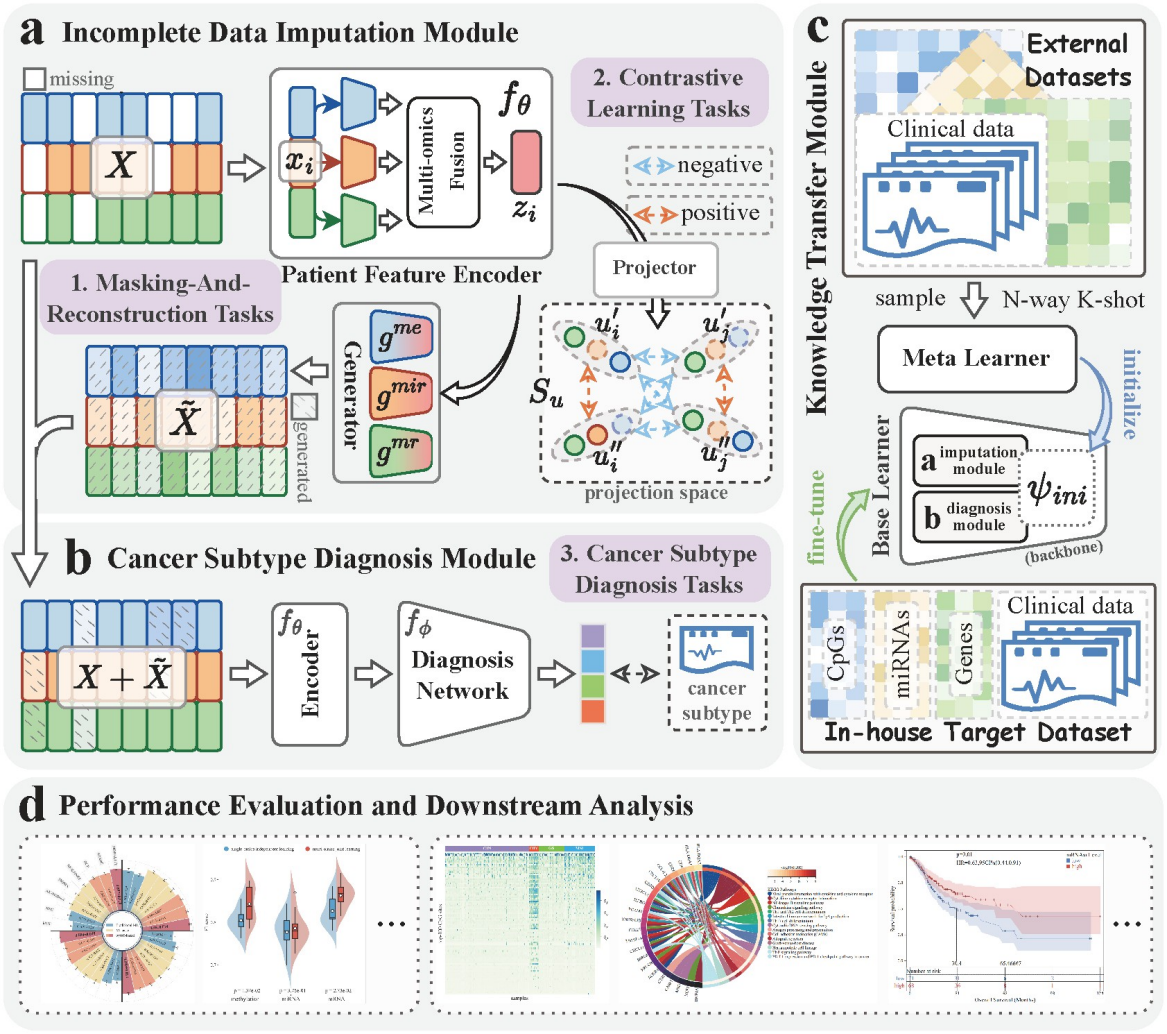
The overview of CancerSD. **(a)** The incomplete data imputation module uses contrastive learning to extract cross-omics consistent features from available patient data. Then, it feeds these features into the generator, facilitating the imputation of missing omics in samples. **(b)** The cancer subtype diagnosis module leverages available and imputed omics of samples to diagnose cancer subtypes. **(c)** The knowledge transfer module follows the meta-learning paradigm, which develops a meta learner and a category-level contrastive loss to mine domain-specific knowledge from external datasets and to initialize a backbone network composed with the representation and diagnosis modules. **(d)** A series of comparison experiments and downstream analyses are conducted to evaluate the performance and application value of CancerSD.

Here, we proposed CancerSD (Fig 1) to integrate incomplete multi-omics data from limited clinical samples for accurate Cancer Subtype Diagnosis. CancerSD designs Contrastive Learning tasks and Masking-and-Reconstruction tasks to effectively impute missing omics using available ones. Subsequently, it fuses both available and imputed data to make accurate subtype diagnoses. To alleviate the negative impact that arose from limited training samples in in-house dataset, CancerSD introduces category-level contrastive loss and extends the meta-learning framework, facilitating further optimization of the diagnostic model. Experimental results on multiple cancer datasets of typical complex cancers demonstrate the effectiveness of CancerSD. It delivers superior performance for cancer subtype diagnosis (e.g., gastric, lung, and breast cancer), offering higher authenticity and better interpretability. Furthermore, extensive analyses of the gastric cancer dataset (TCGA-STAD) indicate that CancerSD can identify discriminative molecules in different subtypes, which have associations with the stemness features of gastric cancer cells. Its scoring for subtypes serves as a valuable prognostic predictor. These results confirm the potential application of CancerSD in assisting clinical decision-making.

## Results

In this study, we testified our CancerSD in various scenarios. To validate its effectiveness in cancer subtype diagnosis, we first compared CancerSD with representative and competitive algorithms on multiple cancer datasets with incomplete data, including gastric cancer (GC), lung cancer, and breast cancer (see Table A in S1 Text). More specifically, we evaluated its performance in both standard supervised learning settings and few-sample scenarios. We then investigated its adaptability to different types of omics data and its capability to impute missing omics data, thereby providing insights into the performance of CancerSD in cancer subtype diagnosis based on incomplete multi-omics data. Finally, we delved into the authenticity and application value of CancerSD for clinical diagnostics. Among the cancer datasets involved in the above experiments, lung cancer and breast cancer have been well studied in terms of multi-omics integration [18, 27, 28], with a wealth of analyses of mechanisms underlying these two cancers. In contrast, there has been relatively little similar exploration of GC datasets, despite GC remaining a significant challenge in global health care [29]. Moreover, GC exhibits distinct molecular and histopathological characteristics, such as significant changes in the tumor microenvironment and a high incidence of mixed histological subtypes [30–32]. These characteristics complicate the diagnosis and treatment for GC, making it an ideal case for evaluating the adaptability and performance of computational models. Therefore, we focus more on evaluating the performance of CancerSD on GC datasets and attempt to explore the pathology underlying GC. We primarily report and analyse the results on GC datasets, while the relevant results for lung and breast cancer are presented in S1 and S2 Text.

### Methodology overview of CancerSD

Our CancerSD has the advantage to accurately diagnose cancer subtypes by leveraging incomplete multi-omics data of patients and to reduce its dependence on the quantity of in-house cancer samples for training by absorbing knowledge from external datasets, as depicted in Fig 1. The CancerSD pipeline comprises four components dedicated to accomplishing reliable and flexible cancer subtype diagnosis in scenarios characterized by incomplete data and scarce samples. (i) It firstly establishes the patient feature encoder, a tensor-based fusion network, to efficiently integrate multi-omics data from samples. (ii) Then, it constructs the missing omics imputation network to reliably impute missing omics of samples, which consists of an encoder, a projector, and multiple omics-specific generators. After that, it defines Contrastive Learning tasks alongside Masking-And-Reconstruction (MAR) tasks to optimize this imputation network. The former explores the consistent patient representations across different augmented views, while the latter utilizes such representations to impute the missing omics data. (iii) Next, it introduces the cancer subtype diagnosis network that fuses available and imputed omics data to calculate the probability of each patient suffered from a particular subtype. (iv) To enable model optimization on the scarce in-house clinical samples, CancerSD further proposes a knowledge transfer network to extract meta-knowledge from external datasets. We wanted to remark that the first three networks are collectively referred as CancerSD backbone or the base learner (CancerSD_b_), while the last network is designated as the meta learner (CancerSD_m_).

The detailed description of the CancerSD framework is presented in S1 Fig. For clarity, we illustrated the operational workflow of CancerSD using the GC dataset as a paradigmatic example. It is important to emphasize that our CancerSD framework can be readily extended to subtype diagnosis for various cancers. The framework takes incomplete multi-omics $X={\left\{X^{m}\right\}}_{m=1}^{M}$ from GC patients as input and finally outputs the probability of them being diagnosed with a certain subtype. Here, incomplete multi-omics implies that certain omics data for some patients are absent due to loss or lack of measurement. *M* denotes the number of omics types, and $X^{m}\in R^{N\times d_{m}}$ is the data matrix for the *m*-th omics with *N* patients (or samples) and *d*_*m*_-dimensional features. Specifically, CancerSD starts by constructing a shared encoder, which encodes the multi-omics features of each patient into an integrated representation. The encoder captures distinctive features of different omics through the omics-specific encoding networks and explores inter-omics relationships using tensor outer products.

Afterward, CancerSD extracts cross-omics underlying information from available omics data of GC patients to reliably impute their missing omics. To this end, CancerSD first generates diverse augmented views by masking certain omics in samples with completely-paired multi-omics data. Then, it employs contrastive learning to guide the patient feature encoder, thereby discerning consistency across different views of the same GC patient. This acquired information is subsequently fed into generators to reconstruct the masked omics and recover the missing omics. Finally, CancerSD inputs the imputed multi-omics data along with available ones into its diagnostic network to discriminate the GC subtypes of patients.

To address the practical challenge of collecting a sufficient number of well-annotated GC samples, CancerSD extends the meta-learning framework to facilitate the circulation of knowledge from external public datasets to the in-house GC samples. It introduces a category-level contrastive loss to minimize differences between samples of the same subtype across different datasets at the distribution level, which aims to selectively learn knowledge from external datasets. Finally, CancerSD utilizes the assimilated knowledge to initialize the backbone, enabling rapid adaptation to the target dataset with limited samples and consequently improving the diagnosis performance.

### CancerSD outperforms existing comparison cancer subtype diagnosis methods in the standard supervised learning setting

To assess the effectiveness of CancerSD, we first evaluated and compared it against representative cancer subtype diagnosis methods. Considering that the existing algorithms are based on conventional supervised learning models, we adjusted the training pipeline of CancerSD to adapt to this scenario for a fair comparison. Specifically, we designated the training set as an external dataset for training and fine-tuning, while the testing set serves as the target dataset for evaluating diagnostic performance. Since the experiments conducted here do not involve cross-dataset knowledge transfer, they validated the effectiveness of CancerSD backbone. Overall, the comparison methods can be broadly categorized into three groups: Traditional Machine Learning (TML), Multi-omics Integration (MI)-based methods, and incomplete Multi-omics Integration (iMI)-based methods. A detailed description of these methods is provided in Section A in S2 Text.

**Fig 2:**
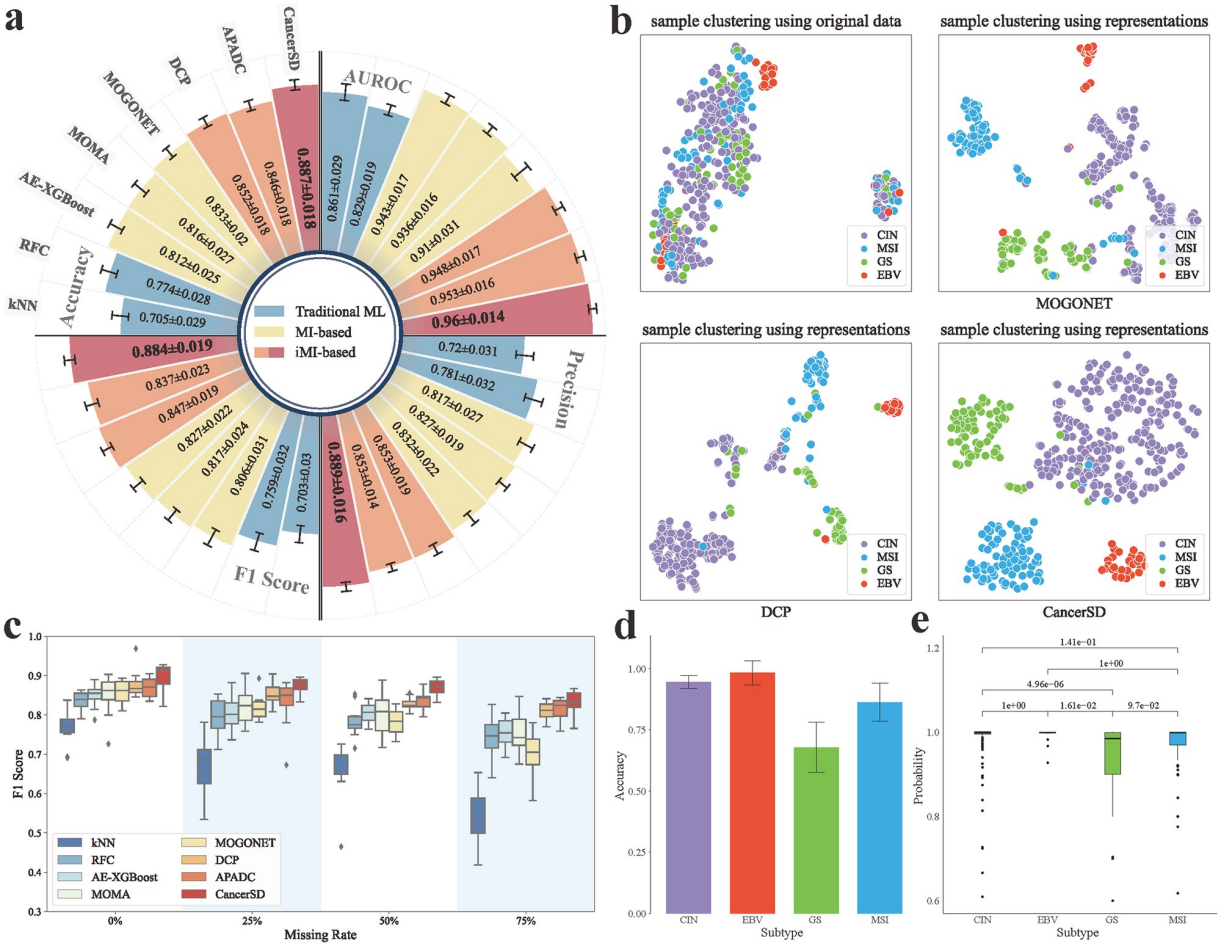
Diagnostic performance on the GC dataset in the standard supervised learning setting. **(a)** The cancer subtype diagnosis performance of CancerSD vs. comparison methods, including kNN, RFC, AE-XGBoost, MOMA, MOGONET, DCP, and APADC on the STAD dataset.**(b)** Sample clustering using original data and embedded representations given by CancerSD and other methods.**(c)** The diagnosis performance of the tested methods under different degrees of omics missingness.**(d)** The diagnostic accuracy of CancerSD for different GC subtypes.**(e)** The diagnostic probability of CancerSD for different GC subtypes.

The experimental results presented in Fig 2a and Tables B-C in S1 Text demonstrate that CancerSD consistently makes top performance in subtype diagnosis. Then, we utilized t-SNE [33] to reduce dimensionality and visualized the embedding spaces of better-performing methods (MOGONET, DCP, and CancerSD). As depicted in Fig 2b, the challenge posed by incomplete data manifests in the distortion of sample distributions, leading to a noticeable division into two distinct clusters. This distortion greatly heightens the difficulty of subtype diagnosis. Despite these challenges, CancerSD still exhibits the best clustering result, with samples of the same subtype more concentrated in the same cluster. These observations underscore the capability of CancerSD in representation learning and its superiority in cancer subtype diagnosis using incomplete multi-omics data. In Section B in S2 Text, we conducted more analyses of the above results, thus gaining a more comprehensive and detailed perspective on the performance differences among different methods.

Next, we evaluated the diagnostic performance of these methods under diverse degrees of multi-omics missingness. We intentionally simulated omics missing scenarios by randomly masking specific omics in samples with completely-paired multi-omics data. Specifically, the simulated missing rates are set at 0%, 25%, 50%, and 75% in sequence, indicating the specified proportion of samples selected for missing certain omics. Fig 2c and Table D in S1 Text demonstrate that CancerSD consistently makes the top performance across all the missing rates. In line with the previous analyses, the performance of the tested methods at different rates manifests a step-like distribution. iMI-based methods generally outshine their MI-based counterparts, while traditional machine learning methods display relatively poor performance. As the simulated missing rate increases, there is a gradual performance decline in all methods, with the most significant decrease witnessed in methods that involve sample similarity calculations, such as kNN and MOGONET. Moreover, it is noteworthy that when multi-omics data for samples is either complete or involves minor incomplete issues, iMI-based methods also marginally outperform MI-based ones, primarily due to the auxiliary tasks constructed for recovering the missing omics. As the degree of omics missing intensifies, a substantial performance gap becomes evident between them. Besides the simulated scenarios of multi-omics data with random missing, we also recognize that the missingness of omics data may not be entirely random in the clinical practice. Therefore, we have conducted experiments focusing on the specific omics absence, displayed the results in Tables E-G in S1 Text, and provided further analysis in Section C in S2 Text.

Finally, we investigated the identification preferences of CancerSD for different GC subtypes, including Epstein-Barr virus (EBV), microsatellite instability (MSI), genomically stable (GS), and chromosomal instability (CIN) categorized by the Cancer Genome Atlas (TCGA) Research Network [34]. As shown in Fig 2d, CancerSD can easily and accurately diagnose the EBV and CIN subtypes while maintaining high accuracy for the MSI subtype. However, CancerSD struggles with the identification of the GS subtype. In Fig 2e, it is evident that the accuracy of CancerSD in diagnosing the GS subtype is significantly lower than others. Based on Fig 2b, we noted that the clusters containing samples of GS and CIN subtypes are consistently close to each other, even overlapping. We further analyzed samples where diagnostic errors occurred in the experiments and find that compared to other subtype pairs, these two subtypes are more likely to be misdiagnosed as each other (see S2 Fig). In fact, Lee et al. [35] observed cases of subtype transition between these two subtypes after metastasis (transitions from CIN to GS and vice versa), while the subtype of metastatic tumors is generally the same as the primary tumor. These findings suggest a potential similarity between the GS and CIN subtypes, which gives rise to the confusion of CancerSD toward these two subtypes.

As mentioned above, the experiments conducted under standard supervised learning settings actually highlight the capabilities of CancerSD_b_. Furthermore, we studied more details for CancerSD_b_, including evaluation its robustness (see S3 Fig and Section D in S2 Text), exploring its optimal architecture (see Table H in S1 Text and Section E in S2 Text), investigating the impact of different data augmentation operations (see S4 Fig and Section F in S2 Text), and examining its sensitivity to changes in hyper-parameters (see S5 Fig and Section G in S2 Text). These experiments and analyses provide a more in-depth and comprehensive perspective on why CancerSD can make superior subtype diagnosis performance.

### CancerSD demonstrates superior diagnostic performance in the few-sample scenario

Cancer subtype diagnosis is a classical few-sample scenario, where in-house datasets often contain only a limited number of samples, posing challenges in optimizing an accurate diagnostic model. Moreover, variations in sample collection sources and biases in sample selection contribute to significant differences among different datasets (i.e., sample distribution disparities, as illustrated in S6 Fig). Disregarding this situation and directly transferring knowledge from external datasets to in-house ones can lead to negative transfer, which potentially undermines the performance of the diagnostic model. To assess the effectiveness of CancerSD in addressing these issues, we constructed knowledge transfer tasks across different datasets. The detailed experimental setups and description of comparison approaches are presented in Section Material and methods.

**Fig 3:**
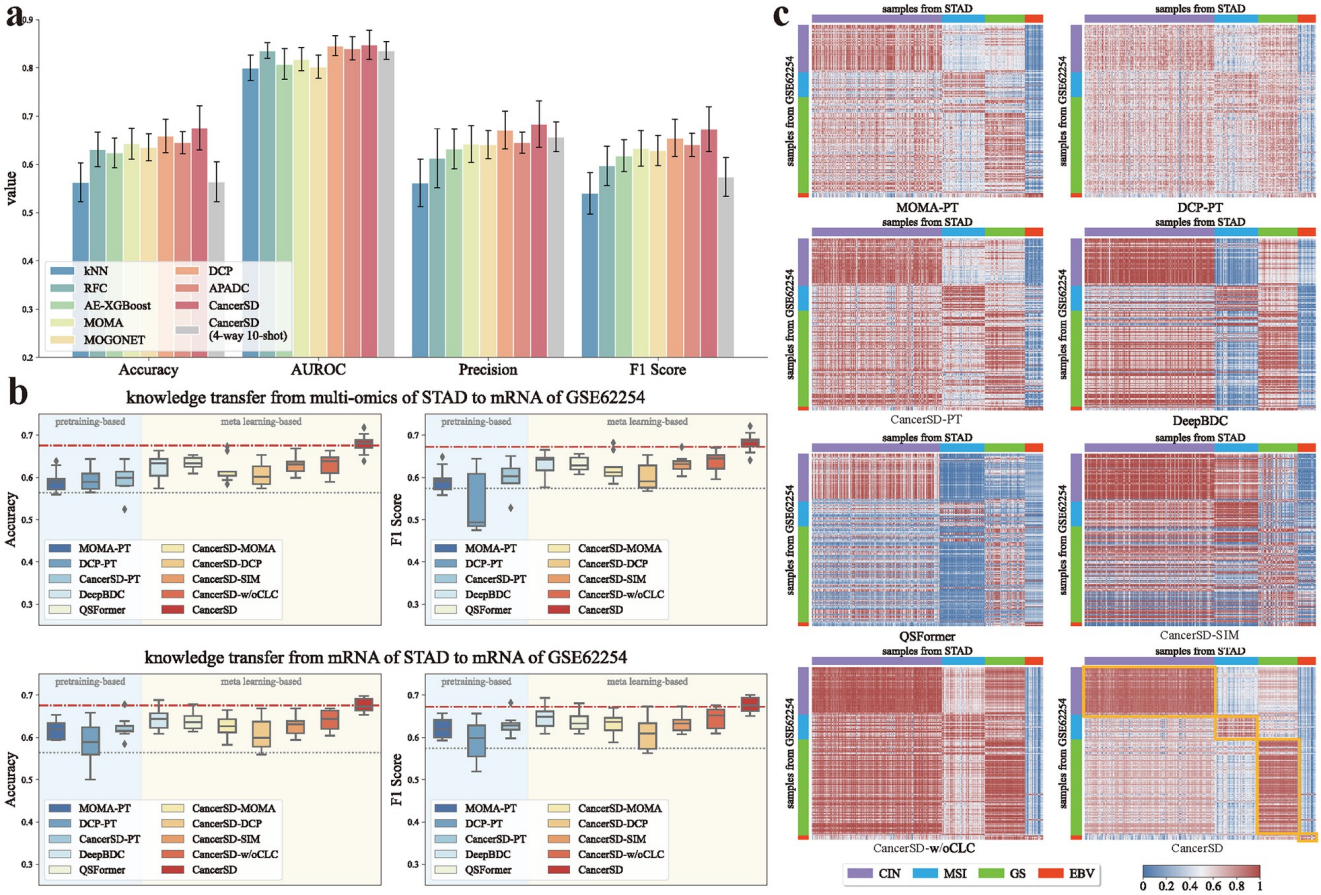
Diagnostic performance on the GC datasets in the few-sample scenario. **(a)** The cancer subtype diagnosis performance of CancerSD vs. comparison methods on the GSE62254 dataset under the standard supervised learning setting.**(b)** The cancer subtype diagnosis performance (Accuracy and F1 Score) of different methods under the multi2mRNA (upper figure) and mRNA2mRNA (lower figure) settings. Here, the red dot dash line represents the performance obtained by optimizing CancerSD with the entire training set, while the gray dotted line indicates that obtained by optimizing CancerSD with a 4-way 10-shot set.**(c)** The similarity of representations for samples from different datasets.

First, we evaluated the performance of certain cancer subtype diagnosis methods on the GSE62254 dataset under the conventional supervised learning settings, which serves as the baseline performance. In this scenario, all methods only use the data from GSE62254, with no knowledge learned from external datasets. As shown in Fig 3a, it is observed that various methods exhibit similar diagnostic performance. Particularly, there is minimal difference among the performance of iMI-based methods, including CancerSD, DCP, and APADC. This phenomenon mainly arises from the fact that GSE62254 contains mRNA expression profiles as the sole omics data, leading all methods to degrade into simple classifiers. Nonetheless, owing to auxiliary tasks such as data reconstruction, iMI-based methods still perform slightly better than others. Additionally, we observed a significant performance decline when training CancerSD with only a small amount of samples (4-way 10-shot, ten samples for each subtype). In fact, by referring to Fig 3a and Table I in S1 Text, we noted that all methods perform poorly under this scenario, which could be attributed to two main reasons. On the one hand, since only mRNA data is accessible in GSE62254 dataset, MI-based and iMI-based methods essentially degrade into single-model classifier, losing their advantage in modeling multi-omics interactions. On the other hand, all compared methods can only use a limited amount of data from GSE62254, preventing sufficient optimization and causing underfitting.

Next, we attempted to transfer knowledge from the STAD dataset, supporting the optimization of diagnostic models on GSE62254. Specifically, we initialized and trained diagnostic models on STAD and subsequently select a small number of samples (4-way 10-shot) from the training set of GSE62254 to fine-tune these models. To include traditional machine learning methods into the experiment, we combined STAD and the data used for fine-tune from GSE62254 into a unified training set, which was then provided to these methods. Here, we explored knowledge transfer under two strategies: from multi-omics data of STAD to the mRNA expression profile of GSE62254 (multi2mRNA) and from the mRNA expression profile of STAD to that of GSE62254 (mRNA2mRNA). As illustrated in Fig 3b, and Tables J-K in S1 Text, We observed that even if TML approaches are granted access to external datasets, they struggle to make reasonable use of these data, due to the lack of effective knowledge extraction strategies. This demonstrates the difficulty of TML methods in adapting to the few-sample scenario, compared to few-shot learning-based approaches. We also found that meta-learning-based methods generally exhibit superior transfer performance compared to pretraining-based ones. This is likely due to the emphasis of meta-learning on the cross-dataset generalization ability of the models, while pretraining strategies focus on models’ performance on the current dataset. Notably, with only a 4-way 10-shot set sampled from the training set, our CancerSD achieves performance comparable to using the entire training set. This observation emphasizes the capability of CancerSD_m_ in cross-dataset knowledge transfer. Moreover, the performance of tested methods under the mRNA2mRNA strategy is superior (or comparable) to that under the multi2mRNA. This could be attributed to the fact that embedding spaces generated from mRNA data in different datasets are more similar in distribution than those generated separately from multi-omics and mRNA data. Consequently, under the mRNA2mRNA strategy, the models can more easily absorb knowledge from external datasets.

Taking results under mRNA2mRNA as example, we further analyzed the effectiveness of CancerSD_m_ and the importance of category-level contrastive loss. In the knowledge transfer task, CancerSD achieves an Accuracy of 67.5%, AUROC of 83.7%, Precision of 73.3%, and F1 Score of 67.6%, outperforming its comparison methods and variations across almost all metrics. The superior performance can be attributed to its powerful backbone and domain-specific knowledge transfer capabilities. Specifically, CancerSD outperforms its variants CancerSD-MOMA and CancerSD-DCP, whose backbone networks are replaced with MOMA and DCP, respectively. This superiority underscores the capability of CancerSD_b_ to effectively impute missing omics data and integrate multiple omics for accurate and reliable cancer subtype diagnosis. On the other hand, the significant performance decline of CancerSD-w/oCLC highlights the importance of the distribution-based category-level contrastive loss. By leveraging this loss, CancerSD can alleviate the sample distribution discrepancy across different datasets and focus on extracting knowledge relevant to the assigned downstream diagnosis tasks from external datasets. Fig 3c supports this perspective by showing that representations obtained by CancerSD for samples of the same subtype from different datasets exhibit higher similarity. This observation indicates that CancerSD effectively captures the consistency of cancer subtypes across different datasets and integrates the consistency into sample representations, thereby improving the similarity among samples of the same subtype. The comparison results also demonstrate that the similarity-based category-level contrastive loss fails to acquire knowledge from datasets with more samples effectively and may even hamper the generalization ability of CancerSD_b_. This is because CancerSD-SIM utilizes the instance-level similarity to cluster samples of the same subtype, which potentially leads to severe overfitting problems and is susceptible to noise and outliers. In contrast, CancerSD attempts to cluster samples of the same subtype at the distribution level, thereby alleviating such issues.

The above analyses provide insights into why CancerSD_m_ can effectively extract knowledge from other datasets. In summary, CancerSD_m_ adopts a meta-learning strategy to mine and transfer meta-knowledge from external datasets and utilizes the category-level contrastive loss to maximize the agreement of distributions between samples with the same subtype across different datasets, thereby improving the diagnostic performance of the model on target dataset.

### Diagnostic performance of CancerSD under different omics data types

Although CancerSD fuses three types of omics data (DNA methylation profiles, miRNA expression profiles, and mRNA expression profiles) for cancer subtype diagnosis in the above experiments, it can readily adapt to different numbers of omics data types. To verify the importance of multi-omics integration in improving the diagnosis performance and to assess the capability of CancerSD in multi-omics integration, we evaluate CancerSD using various combinations of omics data. Here, we only consider samples with completely-paired multi-omics data.

**Fig 4:**
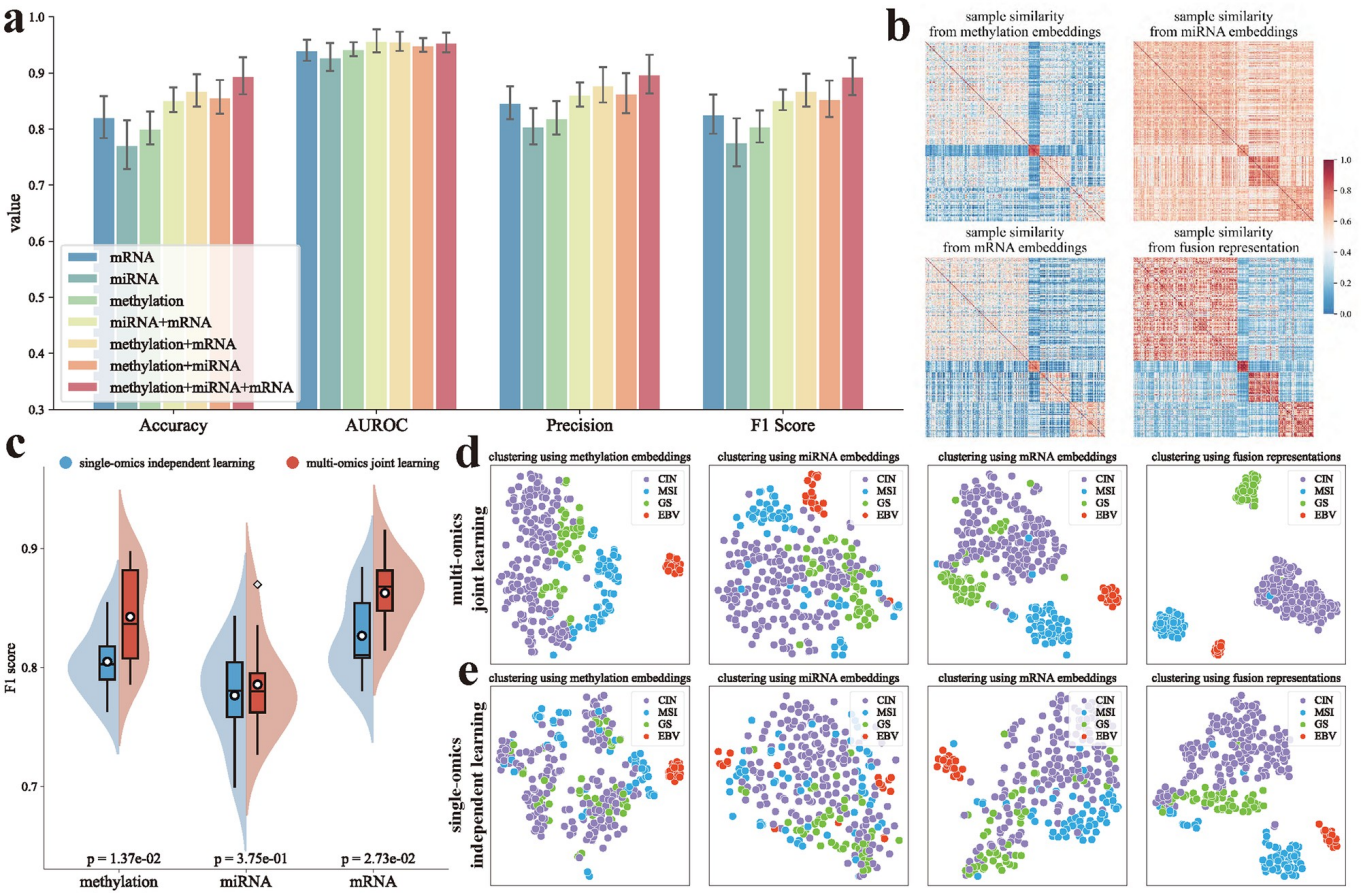
Diagnostic performance under different omics data types. **(a)** Performance comparison for subtype diagnosis using different types of omics. Among them, methylation, miRNA, and mRNA refer to make diagnosis via CancerSD using DNA methylation data, miRNA expression data, and mRNA expression data, respectively; meth+miRNA, miRNA+mRNA, and meth+mRNA refer to diagnosis with two types of omics; meth+miRNA+mRNA refers to diagnosis with three types of omics.**(b)** Sample similarity heatmaps obtained from representation at different levels.**(c)** Diagnostic performance of CancerSD using only a single type of omics under different training strategies.**(d)** Sample clustering based on omics and fusion representations output by CancerSD under multi-omics joint training strategy.**(e)** Sample clustering based on omics and fusion representations output by CancerSD under single-omics independent training strategy.

Fig 4a shows that the diagnostic performance of CancerSD is improved continuously by integrating more omics data. In concrete terms, CancerSD trained with all three omics outperforms the model using a combination of two types of omics. The performance of CancerSD trained with two omics is also superior to that of only single omics. These results highlight the advantages of integrating multiple omics data for more accurate subtype diagnosis. Moreover, it is worth noting that CancerSD trained with mRNA expression data performs best when employing only single omics for training. This finding suggests that mRNA features contain information conducive to distinguishing GC subtypes, potentially harboring valuable biomarkers. In contrast, the performance of CancerSD trained using miRNA data is the poorest. This may be attributed to the lower dimensionality of its original data compared to the other two types of omics (702 vs. 3278 and 4089), which provide less discriminative information for subtyping.

Expanding upon the results mentioned above, we delve deeper into the effectiveness of CancerSD in integrating multi-omics data. First, we focus on the sample similarity pattern as an illustrative example to elucidate the capability of CancerSD in extracting cross-omics consistency information. As depicted in Fig 4b, after mining subtype-related discriminative information in the corresponding omics data using omics-specific feature extractors, samples of the same subtype exhibit relatively high similarity across different omics. CancerSD adeptly identifies and captures this cross-omics consistent pattern of sample similarity, incorporating these patterns into the multi-omics fusion representation. Meanwhile, CancerSD takes into account discrepant patterns across multiple omics to mitigate the impact of erroneously high similarity (i.e., the globally high similarity from miRNA embedding). Consequently, the fusion representation learned by CancerSD more accurately reflects the correct similarity relationships between samples.

Next, we conduct extensive experiments to assess the capability of CancerSD in exploring cooperation between diverse omics data. To this end, we compare the subtype diagnosis performance of CancerSD in two distinct training strategies: (i) multi-omics joint learning, which uses multiple omics data simultaneously to optimize the model; (ii) single-omics independent learning, which uses only a single type of omics to optimize the model each time. As shown in Fig 4c and Table L in S1 Text, it is evident that under the identical condition of diagnosing, CancerSD optimized by multi-omics joint learning more accurately identifies patient subtypes compared to the model optimized by single-omics independent learning strategy. To gain further insights, we investigate the omics embeddings obtained under different training strategies and evaluate the diagnostic potential of multi-omics fusion representations derived from these embeddings. Specifically, we separately construct and optimize multi-omics fusion networks (see Experimental Section) to integrate multiple omics embeddings obtained under joint learning and independent learning strategies. Then, we utilize the resulting fusion representations to perform cancer subtype diagnosis tasks. The results presented in Table L in S1 Text suggest that the fusion representations integrated from omics embeddings learned by joint learning are more conducive to identifying subtypes. Furthermore, we visualize sample clustering for different training strategies. From Fig 4d and 4e, we can find that, in comparison with the output obtained by initially performing single-omics independent learning and then integrating the resulting omics embeddings, the fusion representations obtained through multi-omics joint learning can generate a more compact clustering structure, with more apparent margins between clusters. These observations prove that CancerSD has effectively learned cooperation between different omics during the multi-omics joint optimization process, thereby enhancing the performance of multi-omics fusion.

In summary, multi-omics data can offer more prosperous and more comprehensive patient features. CancerSD effectively integrates these data by extracting cross-omics consistency and cooperation information, significantly improving the performance of cancer subtype diagnosis. To further highlight the superiority of our CancerSD, we also evaluated the diagnostic performance of several comparison methods across different omics types (see Table M in S1 Text), with a concise analysis provided in Section H in S2 Text. In addition to integrating multi-omics, CancerSD also possesses a notable capability in handling missing omics data. Even in cases of extensive omics data missingness (see Table N in S1 Text), CancerSD effectively recovers biologically meaningful expression values, affirming its authenticity and effectiveness in imputing missing omics. The details regarding this aspect are presented in S7–S13 Figs and are discussed more extensively in Section I in S2 Text.

### CancerSD identifies important molecules related to gastric cancer

Identifying important biomarkers is crucial for understanding the underlying mechanisms of GC and interpreting the corresponding diagnostic decision made by CancerSD. To this end, we investigated the importance of each molecular characteristic on the diagnostic outcomes to find potential biomarkers. Specifically, we systematically shuffled the values of each molecular characteristic across all samples in the testing set and then evaluate the diagnostic performance of CancerSD using these modified features. After that, we compared the performance with results obtained when using all features, allowing us to discern the contribution of each molecule to diagnosis tasks, where the diagnosis loss (Eq 10) serves as a quantitative indicator for multi-classification tasks. The more loss increases, the more important the currently permutated molecule is. For a more robust result, we conduct ten random experiments and take the average performance degradation as the final result. Within each type of omics, we selected the top-ranked molecular characteristics for further analysis and validation.

**Fig 5:**
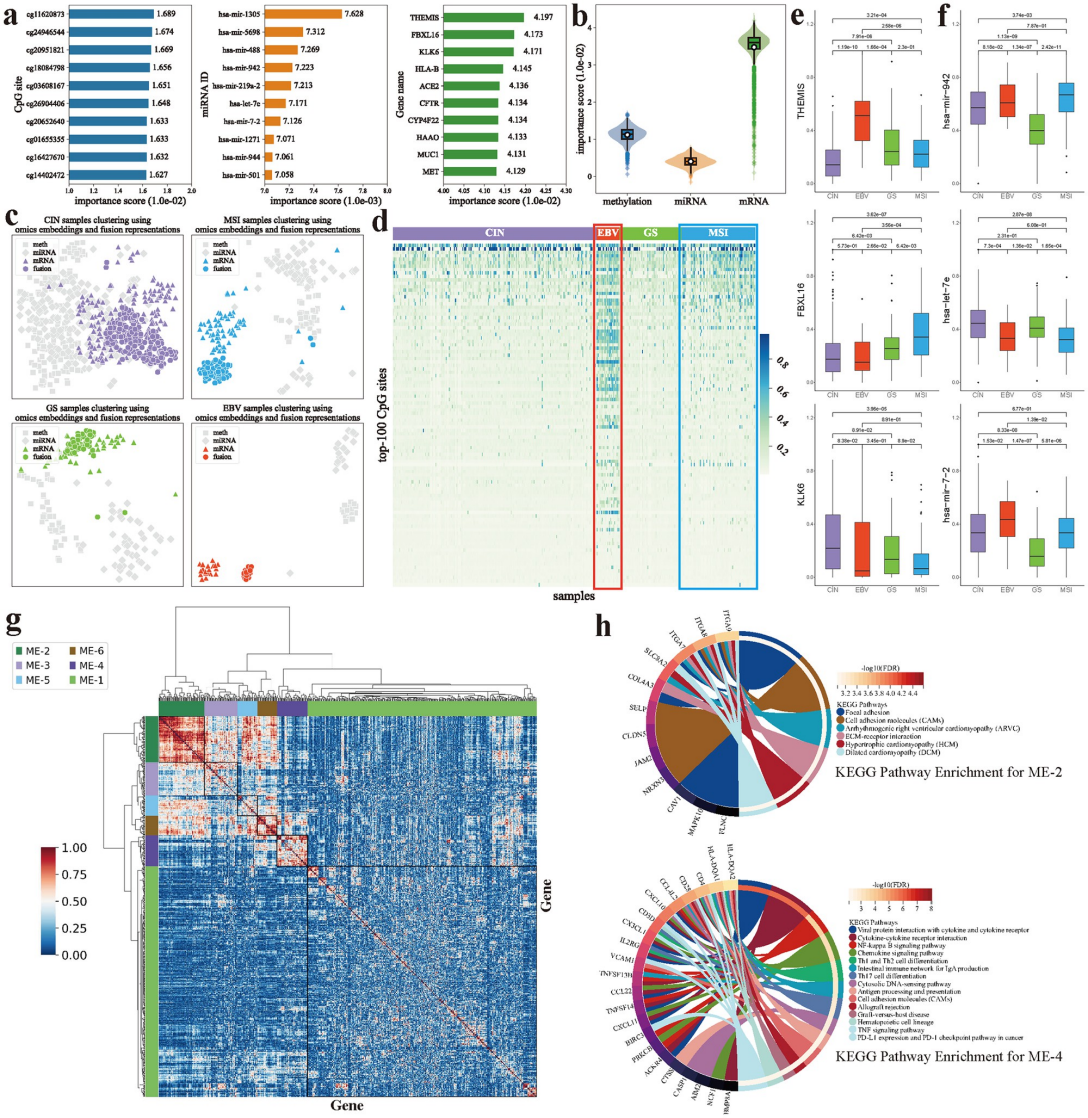
Important molecules identified by CancerSD. **(a)** Importance scores of the top-ranked molecules identified by CancerSD in various omics.**(b)** Differences in the importance scores of molecules across various omics.**(c)** Clustering for samples of different subtypes using omics embeddings and fusion representation output by CancerSD, respectively.**(d)** The methylation levels of the top 100 CpG sites ranked by importance, where the CpG sites are secondary sorting based on the average values across all samples.**(e)** The expression levels of mRNA characteristics across different GC subtypes.**(f)** The expression levels of mRNA and miRNA characteristics across different GC subtypes, where the expression values subjected to log2 transformation and normalization. Wilcoxon rank-sum test is employed to evaluate the differences in the expression levels of specific molecules among patients of distinct subtypes.**(g)** Gene co-expression analysis result for EBV subtype.**(h)** KEGG Pathway Enrichment results for module-2 (ME-2, top) and module-4 (ME-4, bottom), respectively.

First, we presented importance scores of the top ten ranked molecules from each omics. As shown in Fig 5a and 5b, it is evident that there are significant importance differences of molecules across various omics. Among them, mRNA features obtain the highest importance scores, while miRNA features have the lowest, indicating that CancerSD relies more on mRNA expression profiles in the diagnostic decision-making process. This perspective is further highlighted in Fig 5c, where clusters from mRNA embeddings are closer to clusters from fusion representations than from other omics embeddings for patients. We speculated that the prominence of mRNA features may be mainly attributed to two reasons. On the one hand, owing to the higher dimensionality of the raw data, mRNA features can provide richer discriminative information for subtype diagnosis. On the other hand, mRNA expression data more directly reflects the activity of genes and cellular functions. Meanwhile, mRNA expression is influenced by multiple regulatory layers, including DNA methylation and miRNA regulation, among others, potentially more comprehensively reflecting the integrated effects of gene expression regulation. The division of cancer subtypes is often associated with changes in genes. Therefore, mRNA features play a more crucial role in the diagnostic process.

Then, we visualized the expression of molecules identified by CancerSD in each omics type to preliminarily showcase the authenticity of CancerSD in making diagnostic decisions. The molecules selected in this step are detailed in Table 1 (genes and miRNAs are listed) and Table O in S1 Text (CpG sites are listed). For the DNA methylation profile, we explored differences in methylation patterns at CpG sites among patients of distinct subtypes, focusing on the top 100 ranked features. As depicted in Fig 5d, differences in methylation patterns emerge across GC subtypes. Specifically, EBV patients exhibit elevated methylation levels at most CpG sites in comparison to other subtypes, with MSI patients ranking second. Patients with the other two subtypes show a relatively similar DNA methylation pattern. These observations are consistent with the previous study [34]. For miRNA and mRNA expression profiles, we investigated the differences in the expression levels of the top 10 important molecules among various subtypes. Based on Fig 5e–5f and S14 Fig, we could observe that most top-ranked characteristics exhibit significantly different expression levels across various subtypes. These observations indicate that CancerSD primarily relies on molecules that exhibit distinction across different subtypes in the diagnostic process.

**Table 1:** Important molecules identified by CancerSD.

Omics type	Molecule
DNA methylation (10)	***CD9*** *, C19orf51, ENC1, RHPN2,* ***CXCR4*** *, TRAF3IP2, ZFPM1, SLC5A5, ARHGEF7, HLA-F*
mRNA expression (10)	*THEMIS, FBXL16,* ***KLK6***,***HLA-B*** *, ACE2, CFTR, CYP4F22, HAAO,* ***MUC1***,***MET***
miRNA expression (10)	**hsa-mir-1305**, hsa-mir-5698, **hsa-mir-488**, **hsa-mir-942**, **hsa-mir-219a-2**, hsa-let-7e, **hsa-mir-7–2**, hsa-mir-1271, **hsa-mir-944**, hsa-mir-501

Note: Molecules indicated in bold face have been confirmed to be associated with gastric cancer.

Next, we explored the relationship between top-10 important molecules in each omics (see Table 1) and GC. Among them, characteristics derived from DNA methylation and mRNA expression profiles are inferred as their corresponding genes. Notably, nearly half of these molecules have been extensively studied. For instance, Yasumoto et al. [36] discovered that the *CXCR4*/*CXCL12* axis plays a role in the development of peritoneal carcinomatosis, which is an incurable complication during the advanced stages of GC. Subsequent research by Hashimoto et al. [37] revealed that blocking the *CXCR4*/mTOR signaling pathway may contribute to the treatment of this complication. Moreover, Xiang et al. [38] demonstrated that *CXCR4* can cross-activate with *CXCR2*, promoting the epithelial-mesenchymal transition, metastasis, and invasion in GC. Simultaneous inhibition of these two genes has been shown to reduce the metastasis of GC effectively. These studies highlight the close association between *CXCR4* and GC. Besides important genes, the miRNAs identified by CancerSD have also been confirmed to have profound associations with GC. Taking hsa-mir-488 as an example, several studies [39, 40] demonstrated that its overexpression can delay the malignant progression of GC, suggesting that hsa-mir-488 holds promise as a valuable biomarker for the diagnosis and treatment for GC. Alongside the molecules mentioned above, existing studies (see Table P in S1 Text) have reported that *CD9*, *KLK6*, *HLA-B*, *MUC1*, *MET*, hsa-mir-7–2, hsa-mir-944, hsa-mir-942, hsa-mir-219a-2, and hsa-mir-1305 also play an important role in the occurrence, progression, or diagnosis and treatment of GC.

While the relationships between certain molecules in Table 1 and GC remain unclear, some are closely associated with other digestive tract cancers. For example, Fujita et al. [41] found that the overexpression of *ENC1* may suppress the differentiation of colon cells, potentially leading to the development of colorectal cancer. This process could be achieved through JAK2/STAT5/AKT axis-mediated epithelial-mesenchymal transition and stemness. [42] In addition, Than et al. [43] indicated that *CFTR* is a tumor suppressor gene in intestinal cancer. Similarity mechanisms might exist in GC, and further exploration of these genes could contribute to a more profound understanding of GC, as well as elucidating connections and distinctions among various digestive tract cancers.

Finally, we conducted a detailed analysis of important genes identified by CancerSD for each GC subtype, including genes derived from DNA methylation and mRNA expression profiles. During the calculation of diagnosis loss, we individually assessed the impact of feature shuffling on the diagnosis for each subtype. These processes involve partitioning the testing set based on subtypes and separately calculating diagnosis loss for each subtype. By quantifying the increase of the loss, we assign importance to specific characteristics. For the top-10% ranked important genes (619 / 6186) identified for each subtype, we utilized the WGCNA R package [44] and OmicVerse [45] to conduct gene co-expression analysis and select certain co-expression modules for subsequent analysis. Taking the EBV subtype as an example, the selected genes are clustered into six co-expression modules (as illustrated in Fig 5g). Among these modules, genes in module-2, 3, 4, 5, and 6 exhibit significant co-expression relationships, with 75, 54, 50, 33, and 32 genes within each module. We further conducted KEGG enrichment analysis on these five modules, and the results are presented in Fig 5h and Table Q in S1 Text. In module-2 (ME-2), pathways such as ECM-receptor interaction (ko04512) and cell adhesion molecules (ko04514) are prevalent in cancer and play crucial roles in GC [46, 47]. Some pathways enriched in ME-2 are also highly associated with EBV. Liang et al. [48] reported that focal adhesion (ko04510) signal pathways are often dysregulated due to EBV-associated genomic and epigenomic alterations, which may play a crucial role in the development of EBV-associated GC. Other three myocarditis-related pathways (ko05412, ko05410, and ko05414) are associated with a rare but severe complication of EBV infection [49]. In module-4 (ME-4), numerous immune-related pathways are significantly enriched, such as PD-L1 expression and PD-1 checkpoint pathway in cancer (ko05235), TNF signaling pathway (ko04668), antigen processing and presentation (ko04612). Among them, NF-*κ*B signaling pathway (ko04064) exhibits higher positivity in EBV-positive GC than EBV-negative one [50]. It promotes the proliferation of GC cells infected with EBV, which could be attributed to the regulation of the EBV-encoded BARF1 [51]. Similarly, the overexpression of PD-L1 has been reported as a typical characteristic of the EBV subtype [34], and PD-1 inhibition is an effective treatment for patients of this subtype [52]. These two mechanisms are closely related to ko05235 pathway. Cytokine-cytokine receptor interaction (ko04060) is also one of the core pathways dysregulated in EBV-associated GC [48].

Collectively, the aforementioned pathways may play important roles in the development of EBV-associated GC. Therefore, we hypothesized that key genes within these pathways could serve as potential biomarkers or therapeutic targets for the EBV subtype. For example, *CXCL10* and *CXCL11*, both small-molecule cytokines in the CXC chemokine family, are significantly overexpressed in the EBV subtype compared to other subtypes (see S15 Fig). These two genes regulate the migration, differentiation, and activation of immune cells through the *CXCL9/10/11/CXCR3* axis, which is also directly involved in the proliferation and metastasis of cancer cells. [53] Given their roles in guiding immune cells such as T cells and leukocytes to move towards inflammatory or infected sites, [54]*CXCL10/11* may contribute to better immunotherapeutic effects in EBV-positive GC patients. In more detail, *CXCL10/11* are regulated by EBV-related miRNAs, with the former being regulated by ebv-miR-BART1–3p [55] and the latter being regulated by ebv-mir-BHRF1–3. [56] It is possible that EBV promotes the occurrence and development of GC through these pathways, implying the potential of *CXCL10/11* as diagnostic factors for the EBV subtype. Moreover, for other GC subtypes (CIN, GS, MSI), the co-expression and KEGG pathway enrichment results are presented in S16 Fig.

The above results and analysis verify the authenticity and interpretability of CancerSD in cancer subtype diagnosis, which also prove the potential of CancerSD in assisting clinical diagnosis.

### Outcomes of CancerSD are associated with stemness features of gastric cancer subtypes and patient prognosis

In the previous analyses, CancerSD demonstrates the capability to accurately diagnose cancer subtypes using incomplete multi-omics data. Experiments conducted on the GC dataset also indicate its ability to identify key molecular signatures associated with GC. These results provide preliminary evidence of its reliability in assisting clinical diagnosis. To further investigate the role of CancerSD in diagnostic decision-making, we explored its relationship with gastric cancer subtypes and patient prognosis. Stem cells are characterized by their capacity for self-renewal, either infinitely or perpetually, alongside their ability for multi-lineage differentiation, while stemness is defined as the potential of stem cells in these two aspects [57]. Within tumor tissues, a small proportion of relatively stable cells possessing both proliferative and tumor-reconstructing abilities are identified as cancer stem cells or cancer stem-like cells [58]. These cells may cause various tumor malignancies, such as recurrence, metastasis, multidrug resistance, and radioresistance [59]. Thus, determining the stem-cell characteristic of each GC subtype is of significant importance for gaining deeper insights into mechanisms underlying tumor initiation and progression, as well as for the development of effective therapeutic strategies. To this end, we employed the stemness index model [57], known as mRNAsi, to score the stemness features of GC samples and then conduct further analysis.

We first collected gene expression profiles of pluripotent stem cells from the Progenitor Cell Biology Consortium dataset [60, 61] (syn2701943). The data are preprocessed with mean-centering. Subsequently, the stemness signature is identified through the one-class logistic regression algorithm. Next, spearman correlation analysis is performed between the normalized expression matrix of GC samples and the stemness signature. The resulting correlation coefficients are scaled to the range [0, 1] to determine the stemness index. Finally, we assessed the relationship between mRNAsi scores and our CancerSD.

**Fig 6:**
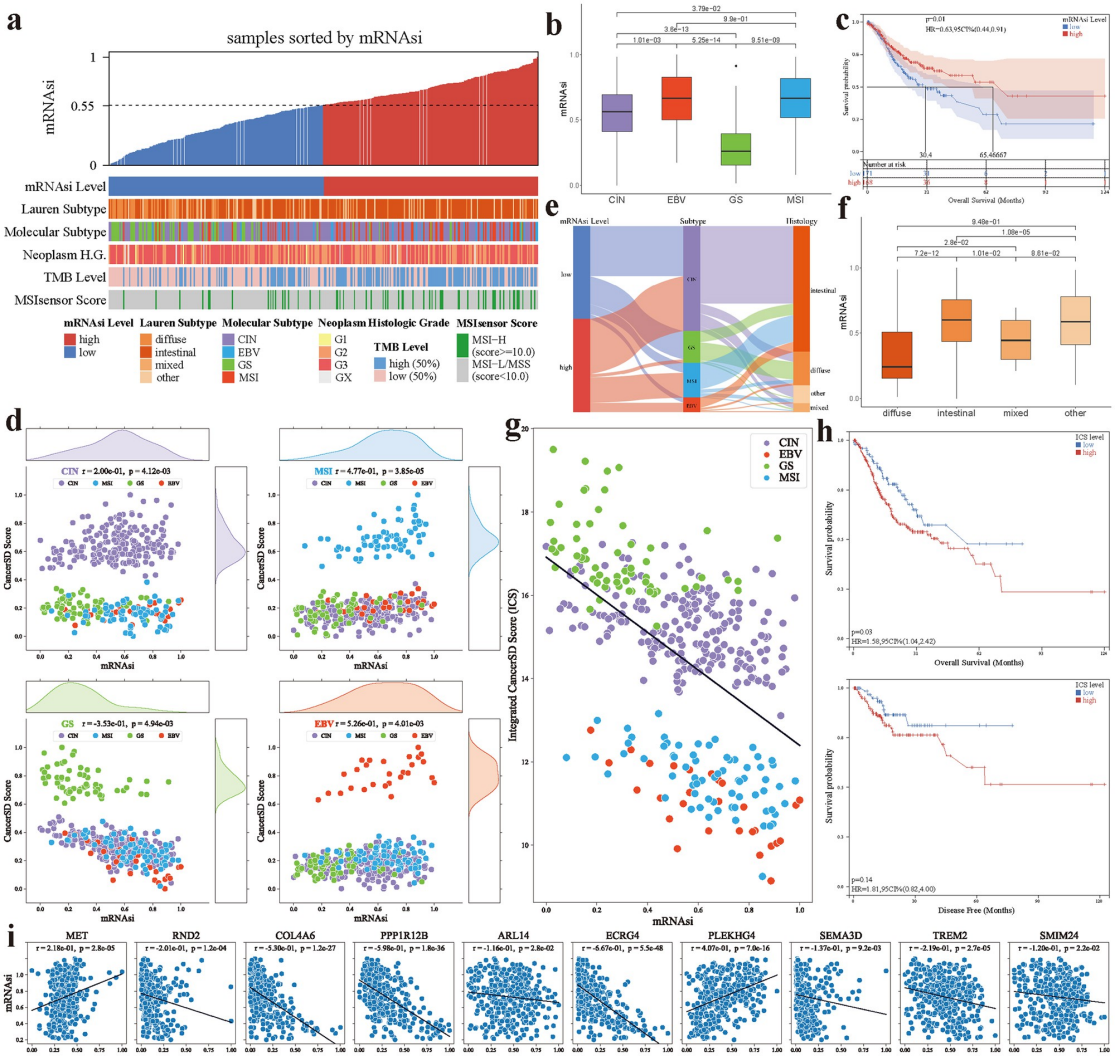
The relationship among CancerSD outcomes, mRNAsi scores, and patient clinical characteristics. **(a)** An overview of the association between the mRNAsi and clinical features. The median of mRNAsi score is used to categorize mRNAsi levels.**(b)** mRNAsi scores across different molecular subtypes.**(c)** Kaplan-Meier survival curves of different mRNAsi levels. Among them, HR and 95CI are abbreviations of Hazard Ratio and 95% Confidence Interval, respectively.**(d)** The relationship between CancerSD scores (with normalization) and mRNAsi scores in GC patients. The former is derived from the output of CancerSD before the *softmax* layer, while the latter is obtained through the mRNAsi model.**(e)** The integrated sankey diagram portrays the underlying correlations across the mRNAsi, molecular subtypes and Lauren classification.**(f)** mRNAsi scores across different Lauren subtypes.**(g)** The relationship between *Integrated CancerSD Score* (ICS) and mRNAsi scores in GC patients.**(h)** Kaplan-Meier survival curves of different ICS levels.**(i)** Correlation of mRNAsi and expression levels of important genes identified by CancerSD. The regression lines in figures are fitted by the corresponding data. The significance in the figure is estimated by pearson correlation coefficient.

From Fig 6a, we observed an association between the stemness index and clinical features in GC patients. In particular, there are significant differences in mRNAsi among patients of distinct GC subtypes (see Fig 6b). While there is typically a negative correlation between mRNAsi and the prognosis of cancer patients [62, 63], an opposite trend is noted in GC [64]. This is further highlighted in Fig 6c, where patients with higher mRNAsi tend to exhibit a favorable prognosis. Consistently, mRNAsi is the lowest in samples of the GS subtype, which corresponds to the poorest prognosis among the four subtypes [65]. Interestingly, a correlation analysis of the stemness index with the CancerSD score for each subtype shows that the GS subtype is significantly negatively correlated with the mRNAsi (*r* = −0.353, *p* = 4.94 × −10^3^, Fig 6d), where CancerSD scores represent the probability of a patient being diagnosed with a certain cancer subtype. There is currently no consensus on why the GS subtype often corresponds to the lowest mRNAsi. Considering the high overlap between samples of GS subtype and of diffuse-type GC (see Fig 6e), we might gain insights into the mechanisms behind this phenomenon from diffuse-type GC [66], which similarly obtains the lowest mRNAsi scores within its corresponding Lauren [67] classification system (see Fig 6f). In addition, CancerSD scores of samples with other subtypes also show significant correlations with mRNAsi. Given the significant correlation between mRNAsi and patient prognosis, the aforementioned observations suggest that GC subtype scores may be associated with the prognosis of GC patients.

To delve deeper into this association, we aggregated CancerSD scores for different subtypes in a manner analogous to TCGA Risk Score [65], yielding the Integrated CancerSD Score (ICS) that estimates patient prognosis risk. To ensure a smoother ICS, we replaced the *softmax* function with the *sigmoid* function, constraining all CancerSD scores to the (0, 1) interval. Specifically, we formulated ICS as follow: ICS = e^ICSraw^, where ICSraw = CIN score + (1—MSI score) + (2 × GS score) + (1—EBV score). Among that, the coefficients preceding the subtype prediction scores were determined based on their relationship with prognosis [65]. Since EBV and MSI are commonly associated with favorable prognosis, we used the inverse of their CancerSD scores. The weighting coefficient for the GS score was assigned to 2, reflecting its strong correlation with poor prognosis. The CIN score remained unchanged, as this subtype is only moderately associated with poor prognosis. Using 16.2 (the optimal cutoff value confirmed by maxstat R package [68]) as the cutoff point, we categorized ICS into low and high levels. As illustrated in Fig 6g, ICS exhibits a significant negative correlation with mRNAsi (*r* = −0.601, *p* = 6.5 × −10^37^), indicating that a high level of ICS may correspond to a poorer patient prognosis. Fig 6h validates this perspective, demonstrating a significant difference in overall survival among patients with different ICS levels (*p* = 0.04). While the stratification of patients based on ICS levels does not show a significant difference in disease-free survival (*p* = 0.16), noticeable distinctions can be observed in Fig 6h. This lack of significance may be attributed to the problem of insufficient data related to disease-free survival, with only 201 out of 415 GC patients possessing corresponding disease-free survival information. To further evaluate the prognostic value of the ICS, we conducted univariate and multivariate Cox proportional hazards regression analyses on ICS and other seven clinical variables. As listed in Table R in S1 Text, in addition to well-known prognostic factors such as T stage, N stage, M stage, and TNM stage, ICS emerged as a significant predictor for overall survival in univariate analysis. Even when incorporating all relevant clinical variables in a multivariate Cox regression analysis, ICS remains an important prognostic factor (HR, 1.7; 95% CI, 1.07–2.70, *p* = 0.03). Collectively, ICS may serve as a potential predictive factor for overall survival and even disease-free survival, highlighting the utility of CancerSD.

We further extended our investigation to the relationship between mRNAsi and the top-ranked genes identified by CancerSD. Specifically, we analyzed the correlation between the expression levels of the top 25 genes (including those identified from DNA methylation and mRNA expression profiles) across all samples and mRNAsi scores. The results are presented in Fig 6i and S17 Fig. We observed that 13 genes exhibit a significant correlation with mRNAsi. Among them, five genes are directly or indirectly involved in various biological processes, exerting impacts on the stemness characteristics of cancer cells. For example, c-Met, the protein product of the *MET* proto-oncogene, has been demonstrated to promote tumor angiogenesis, growth, and metastasis [69]. Several studies [70, 71] reported that c-Met is implicated in the stemness of cancer stem cells in various cancers. In gastric cancer, Yashiro et al. [72] found that the combination of c-Met inhibitors with SN38 may effectively target cancer stem cells in diffuse-type GC. Bahrami et al. [73] also reported that c-Met/ALK inhibitors could reduce the expression of cancer stem cell markers in gastrointestinal cancers. These evidences suggest that elevated expression of *MET* can promote the characteristics of GC stem cells, thereby positively influencing mRNAsi scores. Moreover, *ECRG4* serves as an inhibitory upstream regulator of the NF-*κ*B pathway [74], while the latter is persistently activated in cancer stem cells across various malignancies, participating in several crucial biological processes of cancer stem cells [75]. The role of NF-*κ*B in GC has been widely reported, where its activation can stimulate the proliferation and stemness of GC cells [76]. Ding et al. [77] found that the *PEAK1*-*PPP1R12B* axis can inhibit cell growth and metastasis in colorectal cancer by attenuating the Grb2/PI3K/Akt signaling pathway, and a similar mechanism might exist in GC. Considering the activation effect of PI3K/Akt pathway on the NF-*κ*B system [78], the high expression of genePPP1R12B might indirectly play a role in suppressing the stemness of GC cells. Consequently, we observe a significant negative correlation between the expressions of *ECRG4* and *PPP1R12B* and mRNAsi scores. Besides *MET* mentioned above, *ECRG4* and *PPP1R12B*, another two genes (see Table S in S1 Text), whose expression levels are significantly correlated with mRNAsi scores, are also associated with the stemness features of cancer cells. Although not all of these genes have been confirmed to play a role in GC, there might be similar mechanisms promoting or inhibiting the stemness of GC cells.

In summary, CancerSD scores for samples show a significant correlation with the stemness features of different GC subtypes and patient prognosis. Moreover, the majority of top-ranked important genes identified by CancerSD are closely associated with cancer cell stemness features. These findings once again validate the authenticity and reliability of CancerSD in GC subtype diagnosis, suggesting its potential to assist real-world clinical decision-making.

### CancerSD maintains good performance on multiple cancer datasets

In the above analyses, we primarily discussed experimental results related to GC. To investigate the generalization capability of CancerSD, we also conducted a series of experiments on lung cancer and breast cancer datasets. The relevant results are presented in Tables B, L, T in S1 Text and S8–S9 Figs. Overall, we could draw similar observations from these results as those observed in GC datasets. First, CancerSD exhibits superior (or comparable) performance in subtype diagnosis for lung cancer and breast cancer than the competitive methods. Second, experiments involving multi-omics integration and missing omics imputation on these two cancer datasets further highlight the effectiveness of our method in these regards. Furthermore, we observed that the importance of different omics varies across different cancers during the diagnostic process. Lastly, experiments involving knowledge transfer between two lung cancer datasets once again demonstrate the capabilities of CancerSD in addressing sample scarcity and mitigating negative transfer. In addition to these observations, a more detailed exposition of the relevant experiments and corresponding results is provided in Section J in S2 Text. In summary, CancerSD emerges as an effective and authentic model for cancer subtype diagnosis, which can be readily deployed to different cancers.

## Discussion

In this study, we proposed CancerSD, an end-to-end model designed for cancer subtype diagnosis using limited incomplete multi-omics data. By leveraging the tensor fusion network and contrastive learning, CancerSD can extract more informative representations from available multi-omics data of patients. Then, it employs omics-specific generators with masking and reconstruction mechanisms to reliably recover missing omics. Finally, CancerSD integrates the available and imputed omics data to make accurate subtype diagnoses. To address the issue of limited cancer samples, CancerSD extends the meta-learning framework and introduces a distribution-based category-level contrastive loss, effectively mining relevant knowledge from external datasets. To demonstrate the effectiveness and versatility of CancerSD, we conducted a series of experiments on multiple challenging cancer datasets. The experimental results show that CancerSD significantly outperforms thirteen subtype diagnosis methods and four knowledge transfer methods in most cases. Besides its superior diagnostic performance, CancerSD exhibits good interpretability and maintains high authenticity. It can effectively integrate incomplete multi-omics data and recover biologically meaningful omics data, enabling accurate and reliable subtype diagnosis. More in-depth experiments conducted on the GC dataset further highlight the potential of CancerSD in clinical applications. For important molecular characteristics and related pathways identified by CancerSD, several studies have confirmed their close association with the occurrence and progression of GC, indicating their predictive and therapeutic value. Moreover, our defined *Integrated CancerSD Score* shows a close association with the prognosis of GC patients and holds the potential to serve as an independent predictive factor for patient prognosis.

Despite the notable advancements of CancerSD in cancer subtype diagnosis, there remains room for further improvement. For instance, the usage of tensor fusion may overlook certain prior knowledge contained in multi-omics data, such as regulatory relationships among genes, miRNAs, and mRNAs. Considering these knowledge during modeling contributes to more effective integration of multi-omics data and obtaining better interpretability. Furthermore, CancerSD is currently confined to knowledge transfer across different datasets of the same cancer type. Recognizing potential differences and correlations among different types of cancers, we can further explore more extensive transfer, for example, transferring knowledge from other digestive tract cancers to improve gastric cancer subtype diagnosis. Addressing these aspects in future studies will contribute to the continued refinement and expansion of CancerSD.

## Materials and methods

### Datasets

To study the effectiveness of CancerSD, we apply it to subtype three representative cancers using benchmark datasets: STAD and GSE62254 for gastric cancer (GC) TCGA subtype [34] diagnosis; LUAD, LUSC and CPTAC [79, 80] for lung cancer subtype diagnosis; and BRCA for breast invasive carcinoma PAM50 subtype [81] diagnosis. Three types of omics data are employed for experiments, including DNA methylation profile, miRNA expression profile, and mRNA (protein-coding gene) expression profile. Among the aforementioned datasets, only samples with molecular subtype diagnoses are selected. An overview of these datasets is provided in Table A in S1 Text.

### Data preprocessing

In the data preprocessing stage, we first apply a log2 transformation to the miRNA and mRNA expression data. Then, we filter out features with low variance [14] (the threshold set as 0.2 for the DNA methylation profile, 0.1 for the miRNA expression profile, and 0.8 for the mRNA expression profile). These variance thresholds are consistently used across all experiments. Additionally, we select specific molecular characteristics following the analysis configuration proposed by Hoadley et al. [82] and incorporate them into the filtered features. Consequently, we retain 3287 DNA methylation characteristics, 702 miRNA expression characteristics, and 4089 mRNA expression characteristics. Finally, we individually normalize miRNA and mRNA data to a range of [0, 1] (the original range of DNA methylation data is already within the interval [0, 1], where 0 represents a lower level of methylation, and 1 represents a high level of methylation).

### The shared patient feature encoder

To integrate multi-omics data of patients, CancerSD constructs a module-shared patient feature encoder, which is capable of providing high-quality patient representations for both missing omics imputation and cancer subtype diagnosis. The encoder comprises *M* omics-specific feature extractors and a multi-omics fusion network.

The omics-specific feature extractors are feedforward networks designed to reduce dimensionality and capture discriminative characteristics and patterns of the corresponding omics data as:

$$\begin{array}{c} h_{i}^{m}=f^{m}\left(x_{i}^{m}\right) \end{array}$$ (1)

where $h_{i}^{m}$ is the embedded representation for the *m*-th omics data $x_{i}^{m}$ of the *i*-th patient. *f*^*m*^(⋅) corresponds to the feature extractor of the *m*-th omics.

To capitalize on the advantages of multi-omics data, we employ LMF [83] (Low-rank Multimodal Fusion) and formulate our multi-omics fusion network for data integration. In comparison to alternative methods [14, 84], LMF stands out by its ability to explore cross-omics cooperation while retaining omics-specific information, allowing more informative integration. We first concatenate an extra constant value of 1 after $h_{i}^{m}$, namely ${\tilde{h}}_{i}^{m}={\left[h_{i}^{m};1\right]}^{⊤}$, which can prevent the loss of the original features of each omics. Next, the fusion sub-network performs the outer product operation on ${\tilde{h}}_{i}^{m}$ and transforms the resulting fused tensor into a low-dimensional space as:

$$\begin{array}{c} z_{i}=W\cdot \overset{M}{\underset{m=1}{⊗}}{\tilde{h}}_{i}^{m}+b=(\sum_{r=1}^{R}\overset{M}{\underset{m=1}{⊗}}w_{r}^{m})\cdot \overset{M}{\underset{m=1}{⊗}}{\tilde{h}}_{i}^{m}+b=\overset{M}{\underset{m=1}{⋀}}[\sum_{r=1}^{R}w_{r}^{m}\cdot {\tilde{h}}_{i}^{m}]+b \end{array}$$ (2)

where $W\in R^{d_{1}\times \ldots \times d_{M}\times d_{h}}$ is a (*M* + 1)-order transformation tensor, along with a bias term $b\in R^{d_{h}}$, and *d*_*h*_ is the dimensionality of patient embedded representations. ${⊗}_{m=1}^{M}$ denotes the tensor outer product operation. $\sum_{r=1}^{R}{⊗}_{m=1}^{M}w_{r}^{m}$ is a form of decomposition of **W**, and *R* is the number of low-rank tensors obtained after decomposition. By decomposing **W** to make a separate linear transformation for ${\tilde{h}}_{i}^{m}$, we can significantly reduce the computational complexity of the multi-omics integration. ${⋀}_{m=1}^{M}$ denotes the Hadamard product of *M* vectors: ${⋀}_{m=1}^{M}t_{m}=t_{1}\circ t_{2}\circ \cdots \circ t_{M}$.

In this way, the module-shared patient feature encoder can be formalized as:

$$\begin{array}{c} z_{i}=f_{enc}\left(x_{i}\right)=f_{enc}\left(\overset{M}{\underset{m=1}{⊕}}x_{i}^{m}\right) \end{array}$$ (3)

where *f*_*enc*_ represents the patient feature encoder, $x_{i}={⊕}_{m=1}^{M}x_{i}^{m}$ is the multi-omics data for the *i*-th patient, and ${⊕}_{m=1}^{M}$ is a concatenation operator. In detail, we first split ***x*** *_i_* into *M* omics features, such as DNA methylation, miRNA, and mRNA features used here. Then, each omics data is fed into the corresponding feature extractor *f*^*m*^(⋅). Finally, the resulting omics embeddings are integrated as a fusion representation ***z*** *_i_* through the fusion sub-network.

### Incomplete data imputation module

In real clinical scenarios, it is common to have access to only several or even a single type of omics data for some patients. The multi-omics data for these patients are incomplete, potentially leading to information loss and data bias, resulting in misleading diagnostic outcomes. To avoid risks arising from such incomplete data, we utilize available patient omics to impute their missing ones. To this end, we partition **X** into two parts to perform different training tasks. Specifically, *N*^*cp*^ samples with completely-paired multi-omics data, denoted as $\hat{X}$, are utilized to execute contrastive learning and masking-and-reconstruction (MAR) tasks. Meanwhile, *N*^*inc*^ samples with incomplete data are exclusively employed for the reconstruction tasks.

Firstly, we devise contrastive learning tasks to enhance the representational capacity of information learned by CancerSD for incomplete multi-omics data, providing generators with more informative representations. These processes begin by randomly generating two distinct masking strategies, which are then applied to $\hat{X}$. For each patient, we can garner two different but correlated augmented views, denoted as $x_{i}^{\prime }$ and $x_{i}^{\prime \prime }$, which together constitute the positive pair. Subsequently, $x_{i}^{\prime }$ and $x_{i}^{\prime \prime }$ are fed into the patient feature encoder (see Eq 3), yielding $z_{i}^{\prime }$ and $z_{i}^{\prime \prime }$. These patient representations are further mapped into a projection space, where the contrastive loss is applied. In this way, we obtain higher-level representations $u_{i}^{\prime }$ and $u_{i}^{\prime \prime }$ for the augmented patient views. When taking $\hat{X}$ as the input, a representation set $S_{u}=\left\{u_{1}^{\prime },u_{1}^{\prime \prime },\cdots ,u_{i}^{\prime },u_{i}^{\prime \prime },\cdots ,u_{N^{cp}}^{\prime },u_{N^{cp}}^{\prime \prime }\right\}$ can be assembled, encompassing 2*N*^*cp*^ augmented representations. Finally, we formulate contrastive learning tasks within the projection space with the objective of maximizing the agreement among different augmented views of the same sample. Specifically, given a positive pair $\left(u_{i}^{\prime },u_{i}^{\prime \prime }\right)$, the remaining 2(*N*^*cp*^ − 1) representations in *S*_*u*_ are treated as negative samples [85]. Thus, the loss of the pair can be formulated as:

$$\begin{array}{c} \ell \left(u_{i}^{\prime },u_{i}^{\prime \prime }\right)=-\text{log}\;\frac{\;\text{exp}(\text{cos}(u_{i}^{\prime },u_{i}^{\prime \prime })/\tau )}{\sum_{u\in S_{u}\backslash \left\{u_{i}^{\prime }\right\}}\;\text{exp}(\text{cos}(u_{i}^{\prime },u)/\tau )} \end{array}$$ (4)

where *τ* denotes a temperature parameter. $S_{u}\backslash \left\{u_{i}^{\prime }\right\}$ represents the set without $u_{i}^{\prime }$ from *S*_*u*_. Consequently, the instance-level contrastive loss can be further calculated as:

$$\begin{array}{c} L_{contrastive}=\frac{1}{2N^{cp}}\sum_{i=1}^{N^{cp}}(\ell \left(u_{i}^{\prime },u_{i}^{\prime \prime }\right)+\ell \left(u_{i}^{\prime \prime },u_{i}^{\prime }\right)) \end{array}$$ (5)

Next, we proceed to construct *M* omics-specific generators based on feedforward networks to purposefully impute the missing omics data using latent features extracted from available patient omics as:

$$\begin{array}{c} {\tilde{x}}_{i}^{m}=g^{m}(f_{enc}(\overset{M}{\underset{m=1}{⊕}}\left(\Lambda_{i}^{m}x_{i}^{m}\right))) \end{array}$$ (6)

where ${\tilde{x}}_{i}^{m}\in R^{d_{m}}$ is the generated omics data and *g*^*m*^(⋅) denotes the generator designed for the *m*-th omics. The binary variable $\Lambda_{i}^{m}\in \left\{0,1\right\}$ indicates whether the *m*-th omics of the *i*-th patient is missing or not.

To acquire the imputation ability of the generators, we define MAR tasks on patient features. On the one hand, we utilize *N*^*cp*^ samples with completely-paired multi-omics data to perform the MAR tasks. These tasks involve randomly masking out certain omics of some samples (setting all data within the masked omics to zero) and subsequently reconstructing these masked values. The loss can be calculated as:

$$\begin{array}{c} \ell_{cp}=\frac{1}{N^{cp}}\sum_{i=1}^{N^{cp}}\sum_{m=1}^{M}MSE(g^{m}(f_{enc}(\overset{M}{\underset{m=1}{⊕}}\left(V_{i}^{m}x_{i}^{m}\right))),x_{i}^{m}) \end{array}$$ (7)

where *MSE*(⋅, ⋅) denotes the mean square error loss function, and $V_{i}^{m}\in \left\{0,1\right\}$ indicates whether the *m*-th omics is masked out or not for the *i*-th patient.

On the other hand, for *N*^*inc*^ samples with incomplete data, we only consider the reconstruction loss pertaining to the existing omics as:

$$\begin{array}{c} \ell_{inc}=\frac{1}{N^{inc}}\sum_{j=1}^{N^{inc}}\sum_{m=1}^{M}\Lambda_{j}^{m}MSE\left({\tilde{x}}_{j}^{m},x_{j}^{m}\right) \end{array}$$ (8)

Finally, the generation loss can be calculated as:

$$\begin{array}{c} L_{generation}=\ell_{cp}+\ell_{inc} \end{array}$$ (9)

### Cancer subtype diagnosis module

To alleviate adverse efforts posed by the absence of certain omics, CancerSD fuses both available and imputed omics data to make cancer subtype diagnosis and calculates the diagnosis loss as:

$$\begin{array}{c} L_{diagnosis}=\frac{1}{N}\sum_{i=1}^{N}w_{y_{i}}CE(f_{diag}\left(z_{i}\right),y_{i}) \end{array}$$ (10)

$$\begin{array}{c} z_{i}=f_{enc}(\overset{M}{\underset{m=1}{⊕}}(\Lambda_{i}^{m}x_{i}^{m}+\left(1-\Lambda_{i}^{m}\right){\tilde{x}}_{i}^{m})) \end{array}$$ (11)

where the representation ***z*** *_i_* is obtained by feeding the multi-omics data into the patient feature encoder *f*_*enc*_. In cases where all omics for a patient are available, we input them directly into the encoder; otherwise, we first impute the missing omics and then encode them. *f*_*diag*_ denotes the subtype diagnosis network, *y*_*i*_ is the subtype label of the *i*-th patient, and *CE*(⋅, ⋅) represents the cross entropy loss function. $w_{y_{i}}=N^{tr}/\left(N^{s}\cdot N^{y_{i}}\right)$ is the weight of subtype *y*_*i*_ when calculating the diagnosis loss, which is used to alleviate the problem of sample imbalance. *N*^*tr*^ is the number of samples in the training set, *N*^*s*^ is the number of subtypes, and $N^{y_{i}}$ is the number of samples of subtype *y*_*i*_.

In a word, CancerSD_b_ is an end-to-end architecture involving two modules that are optimized simultaneously in one stage, and the total loss can be calculated based on the individual loss of each module as:

$$\begin{array}{c} L_{base}=\lambda_{1}L_{contrastive}+\lambda_{2}L_{generation}+L_{diagnosis} \end{array}$$ (12)

where λ_1_, and λ_2_ are trade-off parameters among three individual losses. In practice, we set both of them to 1 by default. It is worth noting that although we integrate the losses from each module into $L_{base}$, each loss affects different sub-networks during the backpropagation process. For instance, $L_{contrastive}$ updates *f*_*enc*_ solely and does not impact the other modules. $L_{generation}$ optimizes both *f*_*enc*_ and the omics-specific generator *g*^*m*^. Meanwhile, $L_{diagnosis}$ updates the parameters of *f*_*enc*_ as well as *f*_*diag*_.

By optimizing CancerSD, the missing omics data can be reliably imputed from available ones. Meanwhile, CancerSD extracts essential and meaningful information from incomplete multi-omics data of samples. As a result, it can make a more accurate and flexible cancer subtype diagnosis.

### Knowledge transfer module

Cancer subtype diagnosis is a typical few-sample scenario where well-annotated cancer samples are challenging to collect. To cope with scarce training samples, meta-learning strategies emerge as promising solutions, which enable the backbone model to learn and adapt to new tasks with limited data rapidly. Among them, MAML [86] stands out as a renowned optimization-based [87] meta-learning algorithm. Its exceptional performance, flexibility, and model-agnostic nature make it widely applicable across various tasks and domains. However, the direct application of meta-learning strategies potentially leads to negative transfer issues when significant differences exist in sample distributions across different datasets. In such cases, knowledge learned from external datasets may fail to assist the model in adapting to the target dataset and even mislead its optimization. To address this issue, we aim to align the representations of samples of the same subtype and push away that of samples of different subtypes at the distribution level. Inspired by MAML and Eq 4, we construct a meta learner and formulate a distribution-based category-level contrastive loss to facilitate the desired distribution alignment during knowledge transfer.

As illustrated in Fig 1c, given an external dataset $D^{ext}={\left\{x_{i}^{ext},y_{i}^{ext}\right\}}_{i=1}^{N^{ext}}$ and a target dataset $D^{tgt}={\left\{x_{j}^{tgt},y_{j}^{tgt}\right\}}_{j=1}^{N^{tgt}}$ (where $y_{i}^{ext}$ and $y_{j}^{tgt}$ share the same label space Y), the meta learner CancerSD_m_ aims to learn a better initialization *ψ*_*ini*_ for the backbone CancerSD_b_ parameterized by *ψ*. In detail, CancerSD_m_ first forms a set of sub-batches $\left\{B_{1}^{sub},\cdots ,B_{t}^{sub},\cdots \right\}$ from the external dataset. Each sub-batch $B_{t}^{sub}$ contains a support set $S_{t}$ and a query set $Q_{t}$, both of which are N-way K-shot (each subtype sample *K* patients). Then, CancerSD_m_ undergoes a bi-level optimization procedure with two nested loops: an inner loop for learning sub-batch-specific knowledge and an outer loop for improving the model generalization capability based on multiple sub-batches. The two loops operate on a batch $B={\left\{B_{k}^{sub}\right\}}_{k=1}^{N^{sub}}$ at each iteration, which is composed of *N*^*sub*^ related sub-batches [88]. Meanwhile, to facilitate the distribution alignment, CancerSD_m_ uses a fine-tuning query set $Q_{ft}^{tgt}$ sampled from $D^{tgt}$ along with query sets from the current batch to calculate the category-level contrastive loss in the outer loop. In this step, $Q_{ft}^{tgt}$ is only involved in optimizing the encoder. Finally, we use $Q_{ft}^{tgt}$ to fine-tune the entire initialization *ψ*_*ini*_ learned by CancerSD_m_ and utilize the remaining data of the target dataset to evaluate CancerSD_b_ characterized by these further refined parameters.

Specifically, in the inner loop, CancerSD_m_ changes *ψ*_*ini*_ to sub-batch-specific $\psi_{t}^{\prime }$ for the *t*-th sub-batch by gradient descent on the support set $S_{t}$ as:

$$\begin{array}{c} \psi_{t}^{\prime }\leftarrow \psi_{ini}-\alpha \nabla L_{base}^{B_{t}^{sub}}\left(S_{t};\psi_{ini}\right) \end{array}$$ (13)

where *α* is the inner loop learning rate, $L_{base}^{B_{t}^{sub}}$ represents the sub-batch-related training loss of the base learner (see Eq 12). In this process, CancerSD_m_ separately acquires knowledge from each sub-batch.

To further explore cross-sub-batch knowledge, CancerSD_m_ comprehensively considers all sub-batches within B and calculates the loss using query sets and fine-tuning query set $Q_{ft}^{tgt}$ to update *ψ*_*ini*_ in the outer loop as:

$$\begin{array}{c} \psi_{ini}\leftarrow \psi_{ini}-\beta \nabla_{\psi_{ini}}\sum_{B_{t}^{sub}\in B}(L_{base}^{B_{t}^{sub}}\left(Q_{t};\psi_{t}^{\prime }\right)+L_{clc}\left(Q_{ft}^{tgt},Q_{t};\theta_{t}^{\prime }\right)) \end{array}$$ (14)

where *β* is the outer loop learning rate. $\theta_{t}^{\prime }$ is a subset of $\psi_{t}^{\prime }$, which represents the parameters in the patient feature encoder *f*_*enc*_. In addition, $L_{clc}$ represents the category-level contrastive loss, which can be formulated as:

$$\begin{array}{c} L_{clc}\left(Q_{ft}^{tgt},Q_{t};\theta_{t}^{\prime }\right)=\frac{1}{\left|Y\right|}\sum_{l\in Y}-\text{log}\;\frac{\;\text{exp}\;F_{dist}\left(Q_{ft}^{l},Q_{t}^{l}\right)}{\sum_{k\in Y}\sum_{Q^{k}\in \left\{Q_{ft}^{k},Q_{t}^{k}\right\}}\;\text{exp}\;F_{dist}\left(Q_{ft}^{l},Q^{k}\right)} \end{array}$$ (15)

$$\begin{array}{c} F_{dist}\left(Q_{ft}^{l},Q_{t}^{l}\right)=dist(KDE(f_{enc}\left(Q_{ft}^{l};\theta_{t}^{\prime }\right)),KDE(f_{enc}\left(Q_{t}^{l};\theta_{t}^{\prime }\right))) \end{array}$$ (16)

where *KDE*(⋅) denotes Kernel Density Estimation [89], which is used to construct sample distribution. $Q_{ft}^{l}\subset Q_{ft}^{tgt}$ and $Q_{t}^{l}\subset Q_{t}$ are subsets of samples with subtype *l*. *dist*(⋅, ⋅) is used to measure the distance between two distributions, and we employ Jensen-Shannon divergence in this context, which offers advantages over the Kullback-Leible divergence here due to its symmetry and boundedness properties. When dealing with multiple external datasets, we abstain from considering relationships among them. Instead, we designate the target dataset as the anchor and calculate the contrastive loss individually between $D^{tgt}$ and each $D^{ext}$. In brief, the category-level contrastive loss allows CancerSD_m_ to focus on extracting knowledge relevant to the target dataset from external datasets.

In summary, CancerSD_m_ inherits the merits of meta-learning. It leverages abundant samples available in external datasets to gain a better initialized CancerSD_b_, reduces the dependence on the quantity of training samples, and rapidly adapts to cancer subtype diagnosis tasks on the target dataset with limited samples.

### Experimental settings

To evaluate the effectiveness of CancerSD, we evaluated and compared its diagnostic performance for cancer subtyping in both standard supervised learning and few-sample learning scenarios.

On the one hand, we evaluated and compared various cancer subtype diagnostic methods under the standard supervised learning setting. Specifically, we first applied a random 80/20 split to each cancer dataset, where 80% of the samples were used for training and 20% for testing. To highlight the advantages of CancerSD, we then selected thirteen representative methods for comparison, covering wide-range popular and state-of-the-art approaches: (i) traditional machine learning, including k-Nearest Neighbor classifier (kNN) and Random Forest Classifier (RFC) [90]; (ii) Multi-omics Integration based methods, including AE-XGBoost [91], MOGONET [14], MOMA [15], MOFA+ [92], FactorCL [93], VICReg [94]; (iii) incomplete Multi-omics Integration based methods, including Subtype-GAN [95], scVAEIT [96], DCP [20], and APADC [21]. More detailed descriptions of these methods are provided in Section A in S2 Text. Among the comparison methods, kNN and RFC are trained with the direct concatenation of the preprocessed multi-omics data, while other methods explored effective integration of multi-omics data. It is worth noting that we included all comparative methods in the critical performance comparison experiments. However, considering the architecture or integration strategy similarities among these compared methods, we only deployed seven methods: KNN, RFC, AE-XGBoost, MOMA, MOGONET, DCP, and APADC in the subsequent experiments.

On the other hand, we designed few-sample learning scenarios to investigate the cross-dataset knowledge transfer capability of CancerSD. Taking knowledge transfer tasks on GC datasets as examples, we conducted extensive experiments on the TCGA-STAD and GSE62254 datasets. In these experiments, STAD serves as the external dataset from which we sample *N*-way *K*-shot [97] sub-batches to optimize the models. Each sub-batch consists of a support set S and a query set Q, both S and Q include *K* samples for each of the *N* subtypes. Meanwhile, GSE62254 is treated as the target dataset, which is split into a training set containing one *N*-way *K*-shot set for fine-tuning and a testing set for evaluation. Since GSE62254 only contains mRNA data for samples, we conduct experiments to transfer knowledge from mRNA data and from multi-omics data of STAD to GSE62254. For a more comprehensive evaluation, we also conducted aforementioned experiments on lung cancer datasets (including TCGA-LUAD, TCGA-LUSC, and CPTAC [79, 80]). We selected four competitive approaches for comparison, including MOMA [15]-PT, DCP [20]-PT, QSFormer [98], and DeepBDC [99]. Among them, MOMA-PT and DCP-PT follow a pretraining strategy, where they first undergo pretraining on the external dataset and subsequently fine-tune themselves using a limited amount of samples from the target dataset. QSFormer and DeepBDC are few-shot classification methods based on the meta-learning framework. Moreover, five variants of CancerSD are developed for a more comprehensive evaluation, including (i) CancerSD-PT replaces the meta-learning framework with a pretraining strategy; (ii) CancerSD-MOMA replaces the CancerSD backbone with MOMA; (iii) CancerSD-DCP replaces the CancerSD backbone with DCP; (iv) CancerSD-SIM utilizes representation similarity in the category-level contrastive loss; (v) CancerSD-w/oCLC ignores sample distribution differences among different datasets.

Each experiment randomly repeats ten times to take the average performance and standard deviations, where the diagnosis performance is measured in terms of Accuracy, AUROC, Precision, and average F1 Score weighted by the proportion of corresponding categories.

## Supporting information

S1 Fig Detailed framework of CancerSD. **(a)** CancerSD is an end-to-end deep learning model for cancer subtype diagnosis using limited data with missingness. The initial phase introduces a multi-module shared patient feature encoder to integrate diverse omics data from samples. Then it constructs the imputation and diagnosis modules upon this encoder to perform cancer subtype diagnosis tasks. In addition, it designs a plug-and-play knowledge transfer module to acquire additional knowledge for these two modules in scenarios of scarce samples. Finally, a series of downstream analyses can be conducted based on the outcomes of CancerSD.**(b)** Incomplete data imputation module uses contrastive learning to extract cross-omics consistency features from available patient data and then feeds these features into the generator, facilitating the imputation of missing omics in samples.**(c)** Cancer subtype diagnosis module leverages available and imputed omics of samples to diagnose cancer subtypes.**(d)** Knowledge transfer module follows the meta-learning paradigm, it develops a meta learner and a category-level contrastive loss to mine domain-specific knowledge from external datasets and to initialize backbone network composed with the representation and diagnosis modules.
(TIF)

S2 Fig Actual subtype of patients and the corresponding misdiagnosed subtype. We collect samples that were misdiagnosed in ten repeated experiments and visualize both their true afflictions and the subtypes diagnosed by CancerSD.
(TIF)

S3 Fig Diagnostic performance of CancerSD with random initialization. We fix the dataset (STAD) split and randomly initialize the parameters in CancerSD, thereby evaluating the robustness of CancerSD. In the figure, the red line represents the mean of all experimental results (with ten random initializations for each of the ten random dataset splits, totaling 100 experiments), and the colored shaded area represents the mean±std.
(TIF)

S4 Fig Analysis of the use of different data augmentation. **(a)** F1 Score of CancerSD in gastric cancer subtype diagnosis task under combination of different data augmentation operations.**(b)** The similarity between features resulting from different data augmentation operations and the original features. The *p*-value indicates the significance of the difference (evaluated by Mann-Whitney U test) between similarities obtained from various operations and those from omics-level masking.
(TIF)

S5 Fig Hyper-parameters analysis. **(a)** Performance of CancerSD in gastric cancer subtype diagnosis tasks under different values of temperature factor *τ*.**(b)** Performance of CancerSD in gastric cancer subtype diagnosis tasks under different values of rank *R*.**(c)** Performance of CancerSD in gastric cancer subtype diagnosis task under different values of λ_1_ (weight for instance-level contrastive loss).**(d)** Performance of CancerSD in gastric cancer subtype diagnosis task under different values of λ_2_ (weight for the missing omics generation loss).**(e)** The impact of combining different values for *τ* and *R* (left), and for λ_1_ and λ_2_ (right) on CancerSD (F1 Score).**(f)** Sample clustering under different values of λ_1_.
(TIF)

S6 Fig Sample clustering on different datasets. The STAD and GSE62254 dataset are for gastric cancer molecular subtype classification with EBV, MSI, GS, and CIN subtypes. The ADSC and CPTAC datasets are for lung cancer classification with lung adenocarcinoma (LUAD) and lung squamous cell carcinoma (LUSC), where the CPTAC luad and CPTAC lusc represent samples of these two subtypes in the CPTAC dataset. The BRCA dataset is for breast invasive carcinoma PAM50 subtype classification with Luminal A, Liminal B, Basal-like, HER2-enriched, and Normal-like subtypes.
(TIF)

S7 Fig The imputation performance of CancerSD on the gastric cancer dataset (STAD). **(a)** Sample clustering under different scenarios.**(b)** Similarity between the original samples and the samples after simulating missingness and imputation.**(c)** Differentially expressed genes are obtained separately from the original mRNA data, following by Gene Ontology functional enrichment analysis.**(d)** Differentially expressed genes are obtained separately from the imputed mRNA data, following by Gene Ontology functional enrichment analysis.
(TIF)

S8 Fig The imputation performance of CancerSD on the lung cancer dataset (ADSC). **(a)** Sample clustering under different scenarios.**(b)** Similarity between the original samples and the samples after simulating missingness and imputation.**(c)** Differentially expressed genes are obtained separately from the original mRNA data, following by Gene Ontology functional enrichment analysis.**(d)** Differentially expressed genes are obtained separately from the imputed mRNA data, following by Gene Ontology functional enrichment analysis.
(TIF)

S9 Fig The imputation performance of CancerSD on the breast cancer dataset (BRCA). **(a)** Sample clustering under different scenarios.**(b)** Similarity between the original samples and the samples after simulating missingness and imputation.**(c)** Differentially expressed genes are obtained separately from the original mRNA data, following by Gene Ontology functional enrichment analysis.**(d)** Differentially expressed genes are obtained separately from the imputed mRNA data, following by Gene Ontology functional enrichment analysis.
(TIF)

S10 Fig The imputation performance of CancerSD on STAD dataset with missingness occuring in methylation data. **(a)** Mean Absolute Error (MAE) between original and imputed methylation data in the testing set at different missing rates, and corresponding Root Mean Square Error (RMSE) at each rate.**(b)** Similarity between original and imputed methylation data under various missing rates across different subtypes.**(c)** Sample clustering using the original methylation data.**(d)** Sample clustering using the imputed methylation data under different missing rates.
(TIF)

S11 Fig The imputation performance of CancerSD on STAD dataset with missingness occuring in miRNA data. **(a)** Mean Absolute Error (MAE) between original and imputed miRNA data in the testing set at different missing rates, and corresponding Root Mean Square Error (RMSE) at each rate.**(b)** Similarity between original and imputed miRNA data under various missing rates across different subtypes.**(c)** Sample clustering using the original miRNA data.**(d)** Sample clustering using the imputed miRNA data under different missing rates.
(TIF)

S12 Fig The imputation performance of CancerSD on STAD dataset with missingness occuring in mRNA data. **(a)** Mean Absolute Error (MAE) between original and imputed mRNA data in the testing set at different missing rates, and corresponding Root Mean Square Error (RMSE) at each rate.**(b)** Similarity between original and imputed mRNA data under various missing rates across different subtypes.**(c)** Sample clustering using the original mRNA data.**(d)** Sample clustering using the imputed mRNA data under different missing rates.
(TIF)

S13 Fig Efficiency of imputation algorithms. (TIF)

S14 Fig The expression levels of mRNA and miRNA characteristics across different gastric cancer subtypes. The expression values subjected to log2 transformation and normalization. Wilcoxon rank-sum test is employed to evaluate the differences in the expression levels of specific molecules among patients of distinct subtypes.
(TIF)

S15 Fig The gene expression levels of CXCL10 and CXCL11 across different gastric cancer subtypes. The expression values subjected to log2 transformation and normalization. Wilcoxon rank-sum test is employed to evaluate the differences in the expression levels of specific molecules among patients of distinct subtypes.
(TIF)

S16 Fig Analysis of gene co-expression and KEGG pathway enrichment results in gastric cancer subtype of CIN, GS, and MSI. (TIF)

S17 Fig Correlation of mRNAsi and expression levels of top-25 important genes identified by CancerSD. The regression lines in figures are fitted by the corresponding data. The significance in the figure is estimated by pearson correlation coefficient.
(TIF)

S1 Text Supplementary Tables. Tables A-V.
(PDF)

S2 Text Supplementary Discussions and Analyses. Sections A-J.
(PDF)
