# Supplementary figures and images for: Cancer molecular subtyping using limited multi-omics data with missingness
Source: PLoS Comput Biol. 2024 Dec 26;20(12):e1012710. doi: 10.1371/journal.pcbi.1012710 (PMC11709273; doi:10.1371/journal.pcbi.1012710)

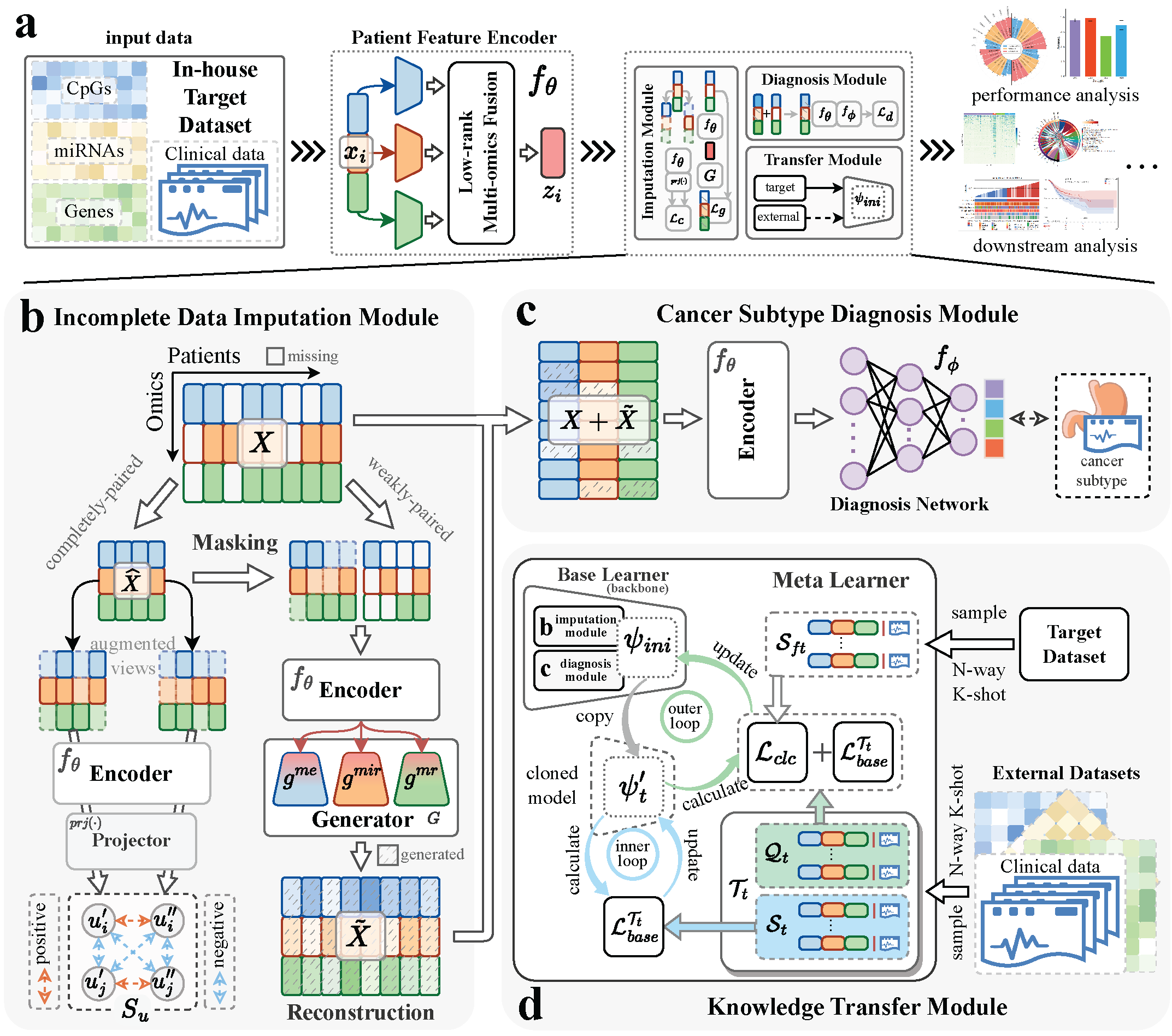

Supplement: S1 Fig — (a) CancerSD is an end-to-end deep learning model for cancer subtype diagnosis using limited data with missingness. The initial phase introduces a multi-module shared patient feature encoder to integrate diverse omics data from samples. Then it constructs the imputation and diagnosis modules upon this encoder to perform cancer subtype diagnosis tasks. In addition, it designs a plug-and-play knowledge transfer module to acquire additional knowledge for these two modules in scenarios of scarce samples. Finally, a series of downstream analyses can be conducted based on the outcomes of CancerSD.(b) Incomplete data imputation module uses contrastive learning to extract cross-omics consistency features from available patient data and then feeds these features into the generator, facilitating the imputation of missing omics in samples.(c) Cancer subtype diagnosis module leverages available and imputed omics of samples to diagnose cancer subtypes.(d) Knowledge transfer module follows the meta-learning paradigm, it develops a meta learner and a category-level contrastive loss to mine domain-specific knowledge from external datasets and to initialize backbone network composed with the representation and diagnosis modules. (TIF) [file pcbi.1012710.s001.tif]

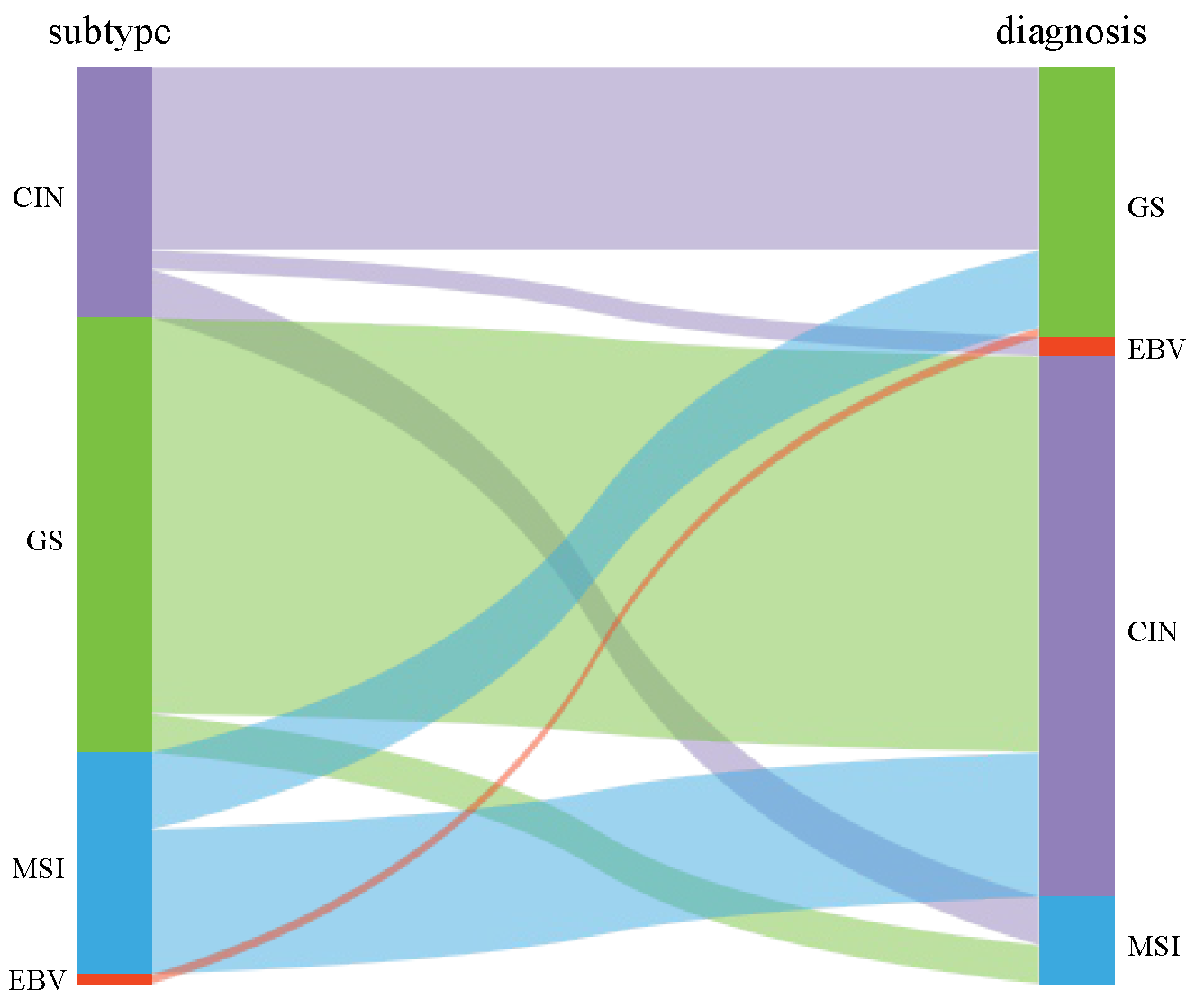

Supplement: S2 Fig — We collect samples that were misdiagnosed in ten repeated experiments and visualize both their true afflictions and the subtypes diagnosed by CancerSD. (TIF) [file pcbi.1012710.s002.tif]

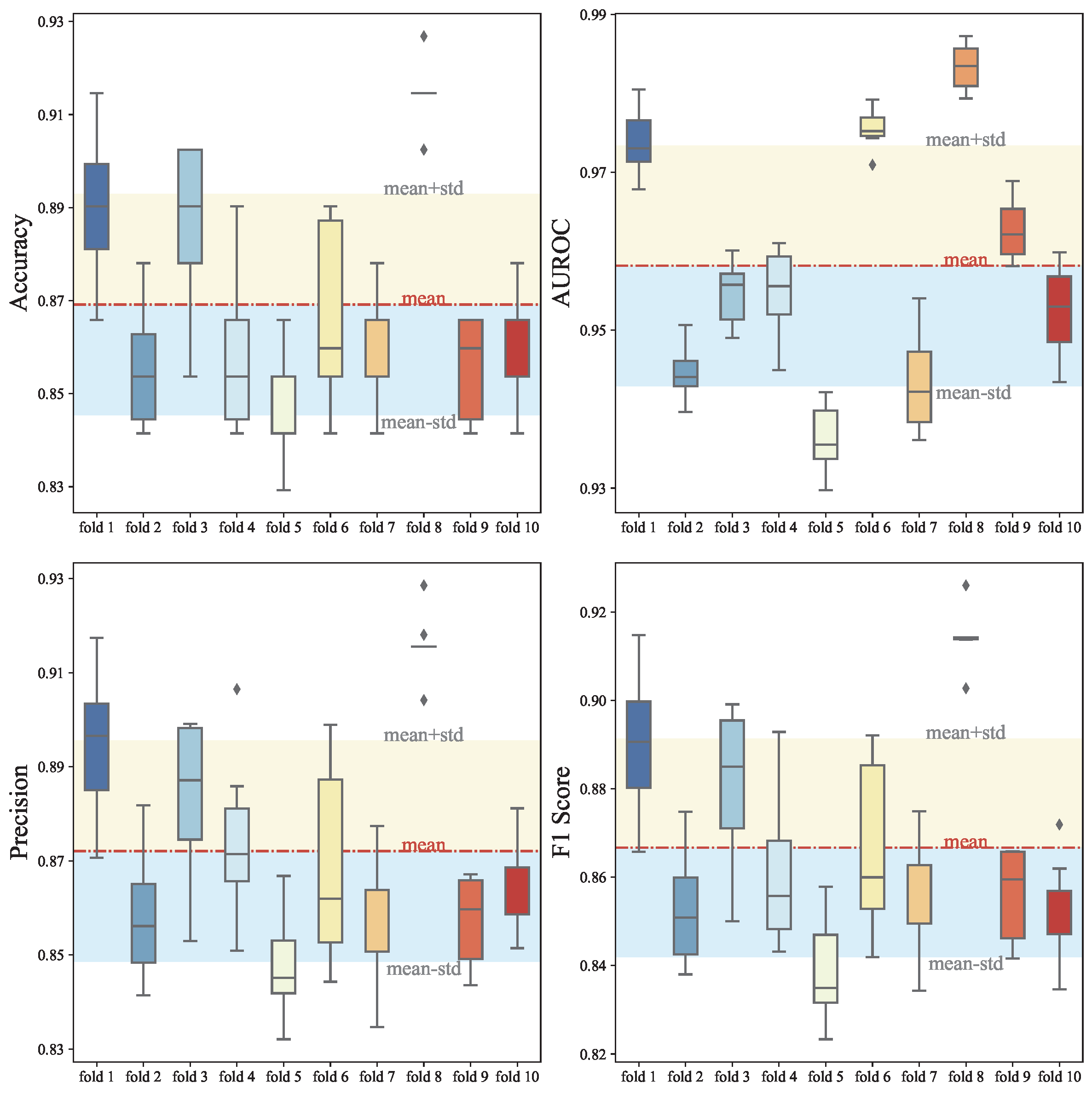

Supplement: S3 Fig — We fix the dataset (STAD) split and randomly initialize the parameters in CancerSD, thereby evaluating the robustness of CancerSD. In the figure, the red line represents the mean of all experimental results (with ten random initializations for each of the ten random dataset splits, totaling 100 experiments), and the colored shaded area represents the mean±std. (TIF) [file pcbi.1012710.s003.tif]

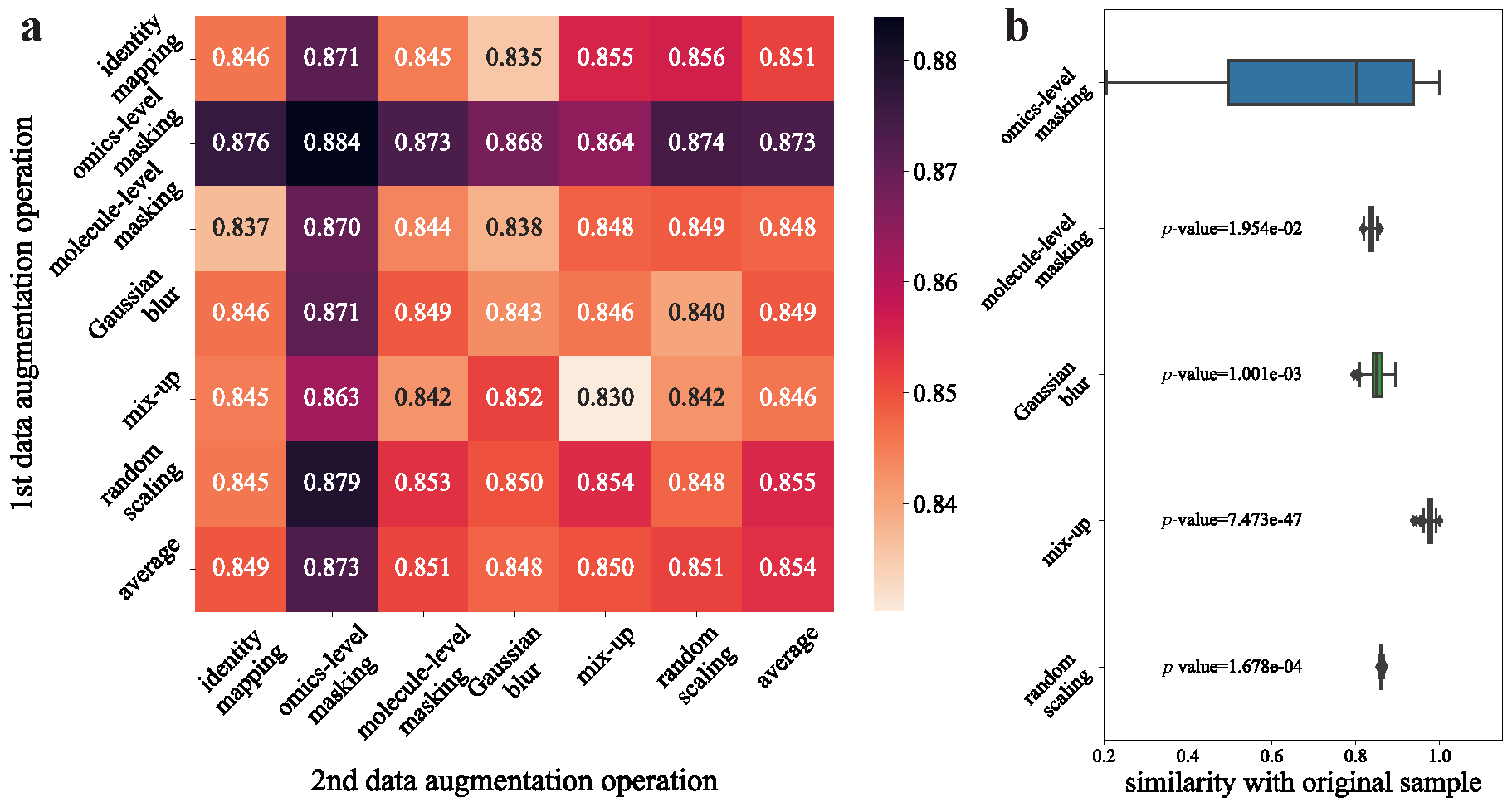

Supplement: S4 Fig — (a) F1 Score of CancerSD in gastric cancer subtype diagnosis task under combination of different data augmentation operations.(b) The similarity between features resulting from different data augmentation operations and the original features. The p-value indicates the significance of the difference (evaluated by Mann-Whitney U test) between similarities obtained from various operations and those from omics-level masking. (TIF) [file pcbi.1012710.s004.tif]

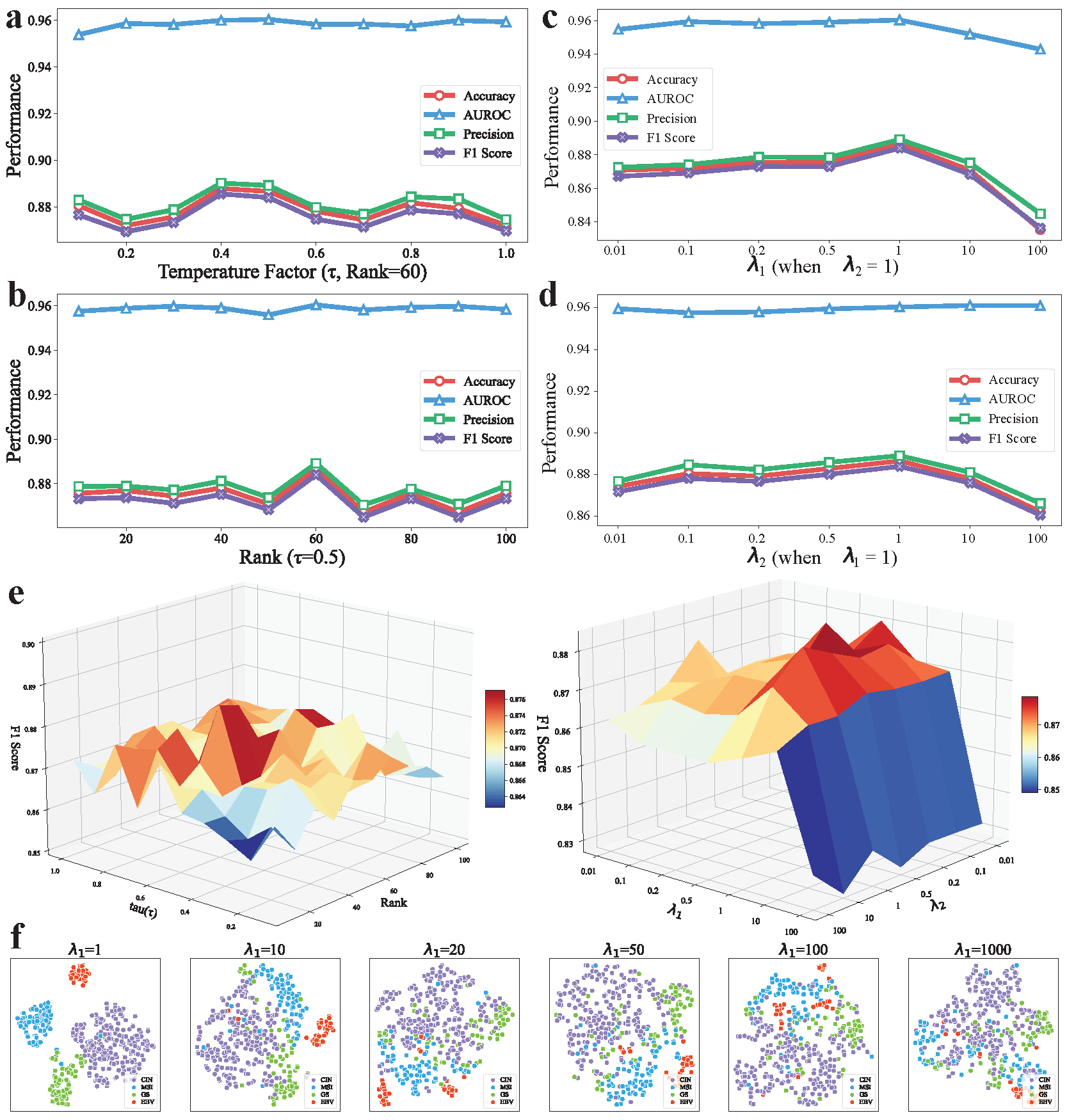

Supplement: S5 Fig — (a) Performance of CancerSD in gastric cancer subtype diagnosis tasks under different values of temperature factor τ.(b) Performance of CancerSD in gastric cancer subtype diagnosis tasks under different values of rank R.(c) Performance of CancerSD in gastric cancer subtype diagnosis task under different values of λ1 (weight for instance-level contrastive loss).(d) Performance of CancerSD in gastric cancer subtype diagnosis task under different values of λ2 (weight for the missing omics generation loss).(e) The impact of combining different values for τ and R (left), and for λ1 and λ2 (right) on CancerSD (F1 Score).(f) Sample clustering under different values of λ1. (TIF) [file pcbi.1012710.s005.tif]

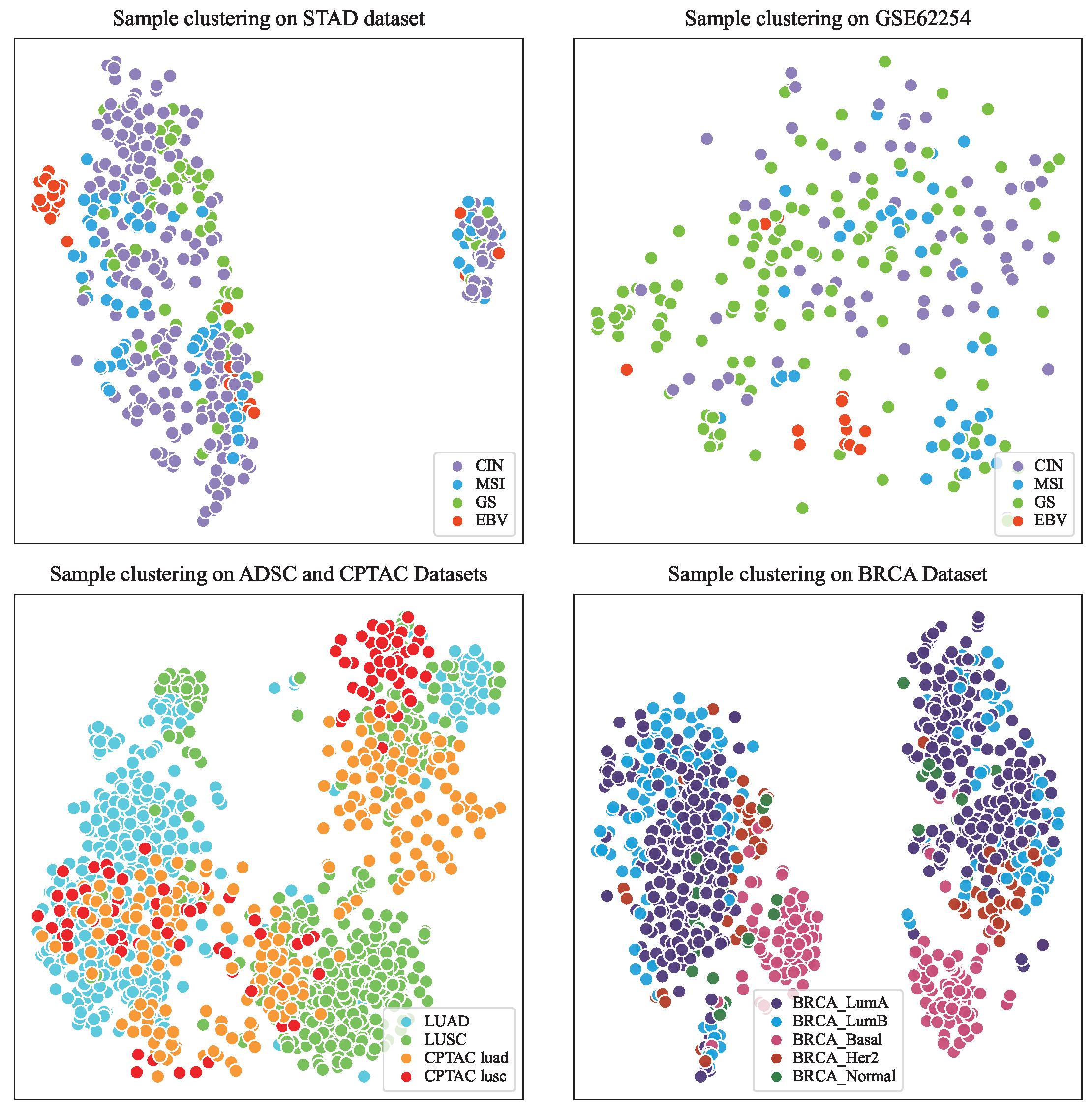

Supplement: S6 Fig — The STAD and GSE62254 dataset are for gastric cancer molecular subtype classification with EBV, MSI, GS, and CIN subtypes. The ADSC and CPTAC datasets are for lung cancer classification with lung adenocarcinoma (LUAD) and lung squamous cell carcinoma (LUSC), where the CPTAC luad and CPTAC lusc represent samples of these two subtypes in the CPTAC dataset. The BRCA dataset is for breast invasive carcinoma PAM50 subtype classification with Luminal A, Liminal B, Basal-like, HER2-enriched, and Normal-like subtypes. (TIF) [file pcbi.1012710.s006.tif]

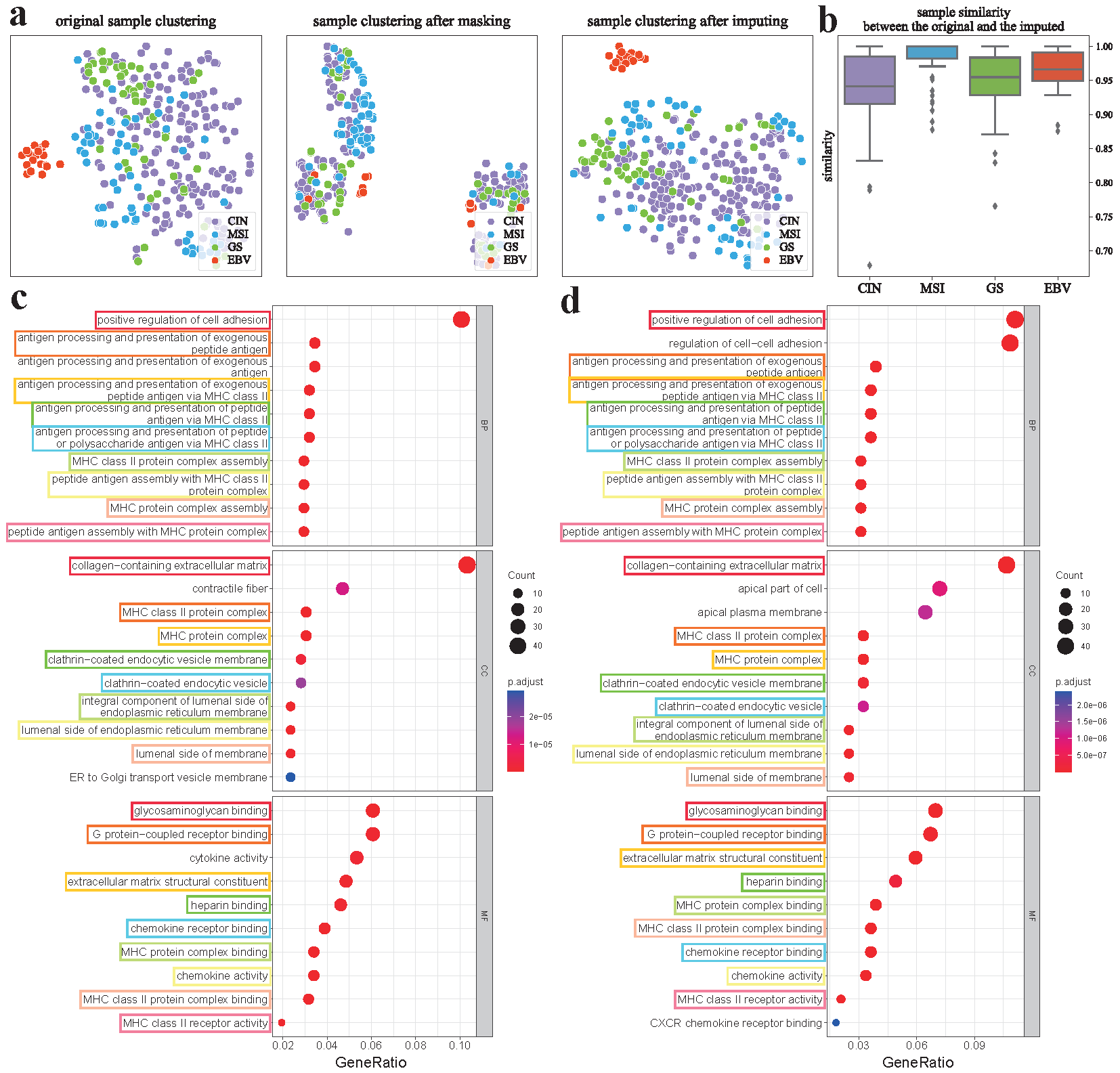

Supplement: S7 Fig — (a) Sample clustering under different scenarios.(b) Similarity between the original samples and the samples after simulating missingness and imputation.(c) Differentially expressed genes are obtained separately from the original mRNA data, following by Gene Ontology functional enrichment analysis.(d) Differentially expressed genes are obtained separately from the imputed mRNA data, following by Gene Ontology functional enrichment analysis. (TIF) [file pcbi.1012710.s007.tif]

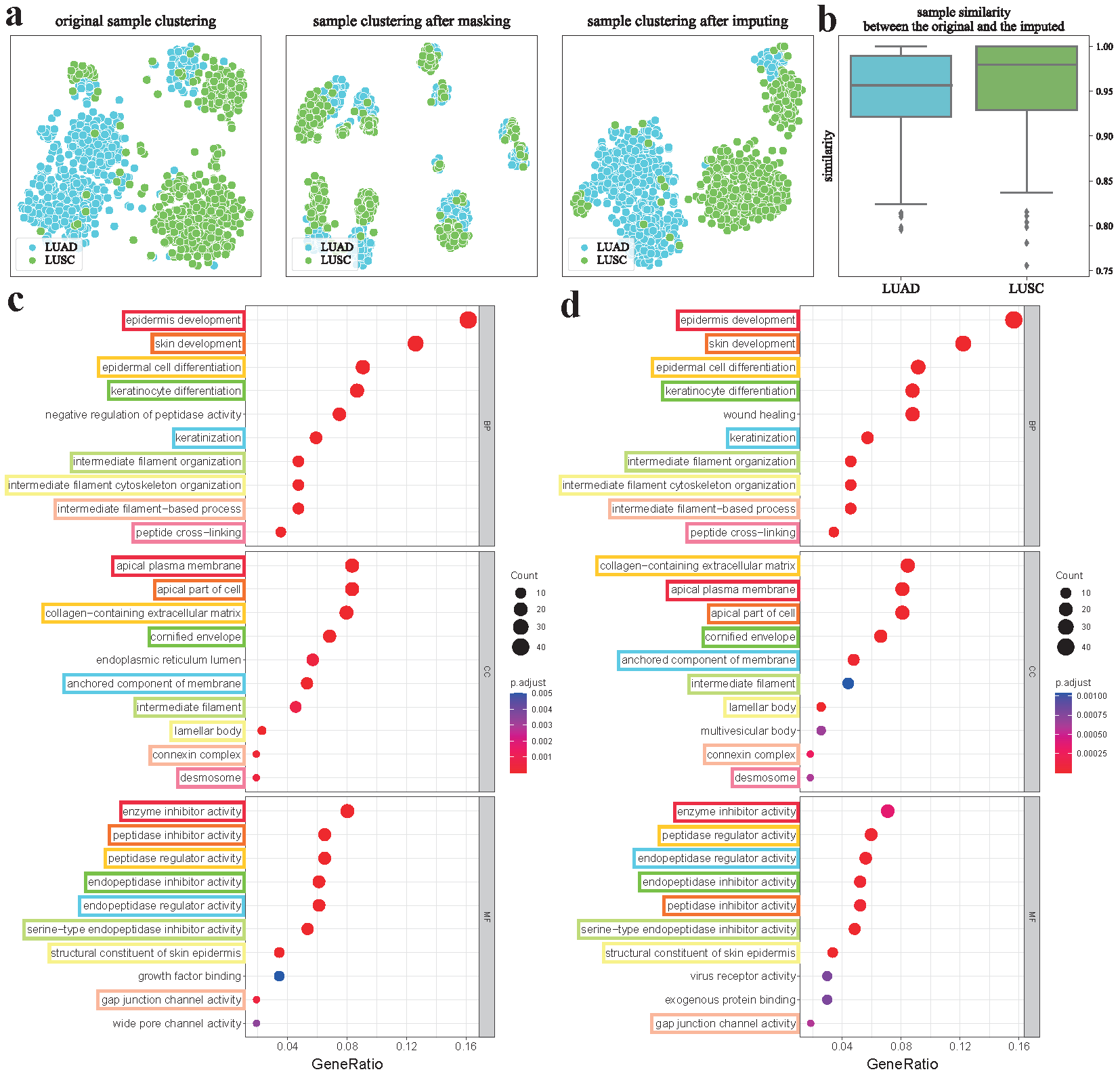

Supplement: S8 Fig — (a) Sample clustering under different scenarios.(b) Similarity between the original samples and the samples after simulating missingness and imputation.(c) Differentially expressed genes are obtained separately from the original mRNA data, following by Gene Ontology functional enrichment analysis.(d) Differentially expressed genes are obtained separately from the imputed mRNA data, following by Gene Ontology functional enrichment analysis. (TIF) [file pcbi.1012710.s008.tif]

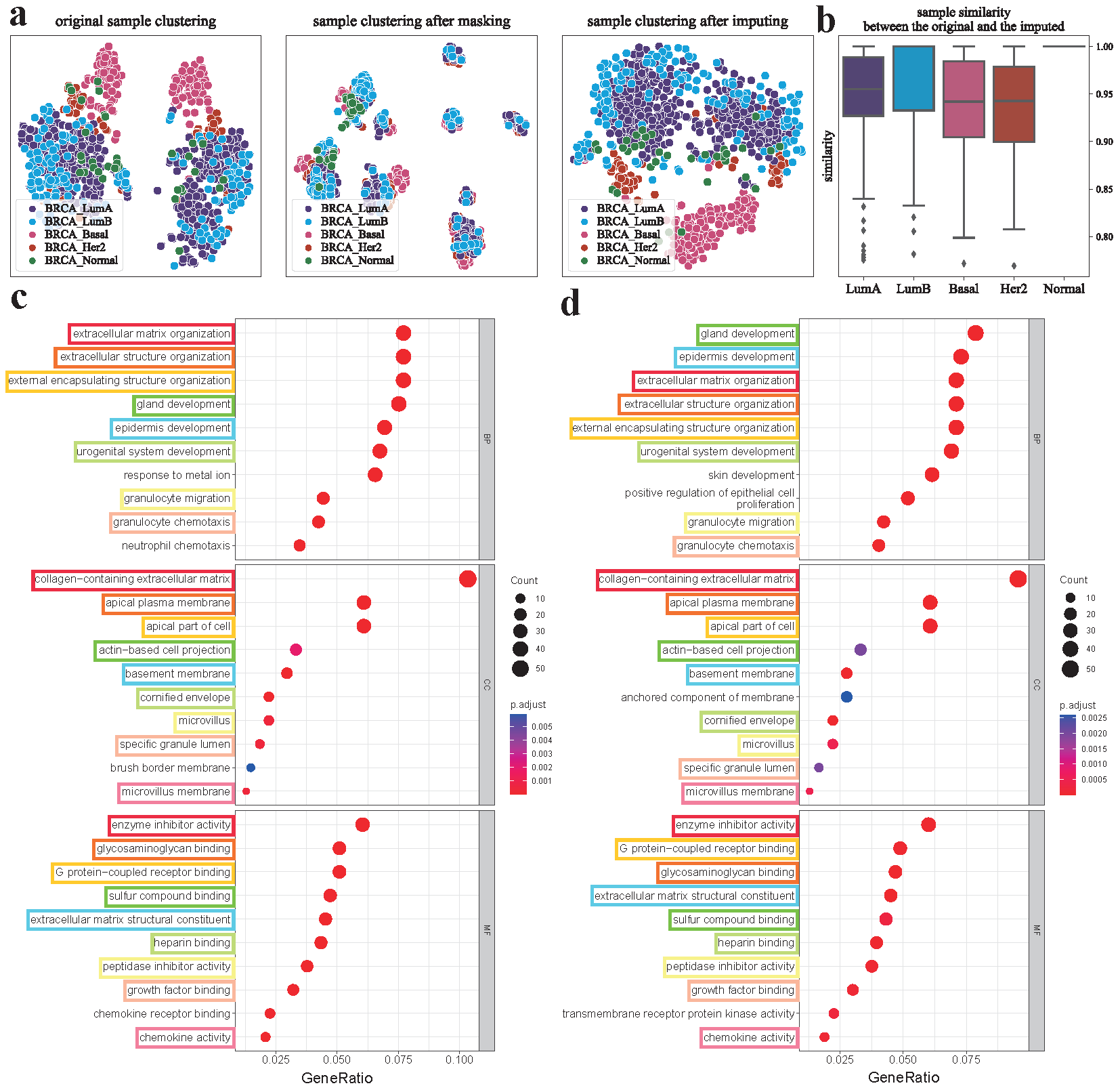

Supplement: S9 Fig — (a) Sample clustering under different scenarios.(b) Similarity between the original samples and the samples after simulating missingness and imputation.(c) Differentially expressed genes are obtained separately from the original mRNA data, following by Gene Ontology functional enrichment analysis.(d) Differentially expressed genes are obtained separately from the imputed mRNA data, following by Gene Ontology functional enrichment analysis. (TIF) [file pcbi.1012710.s009.tif]

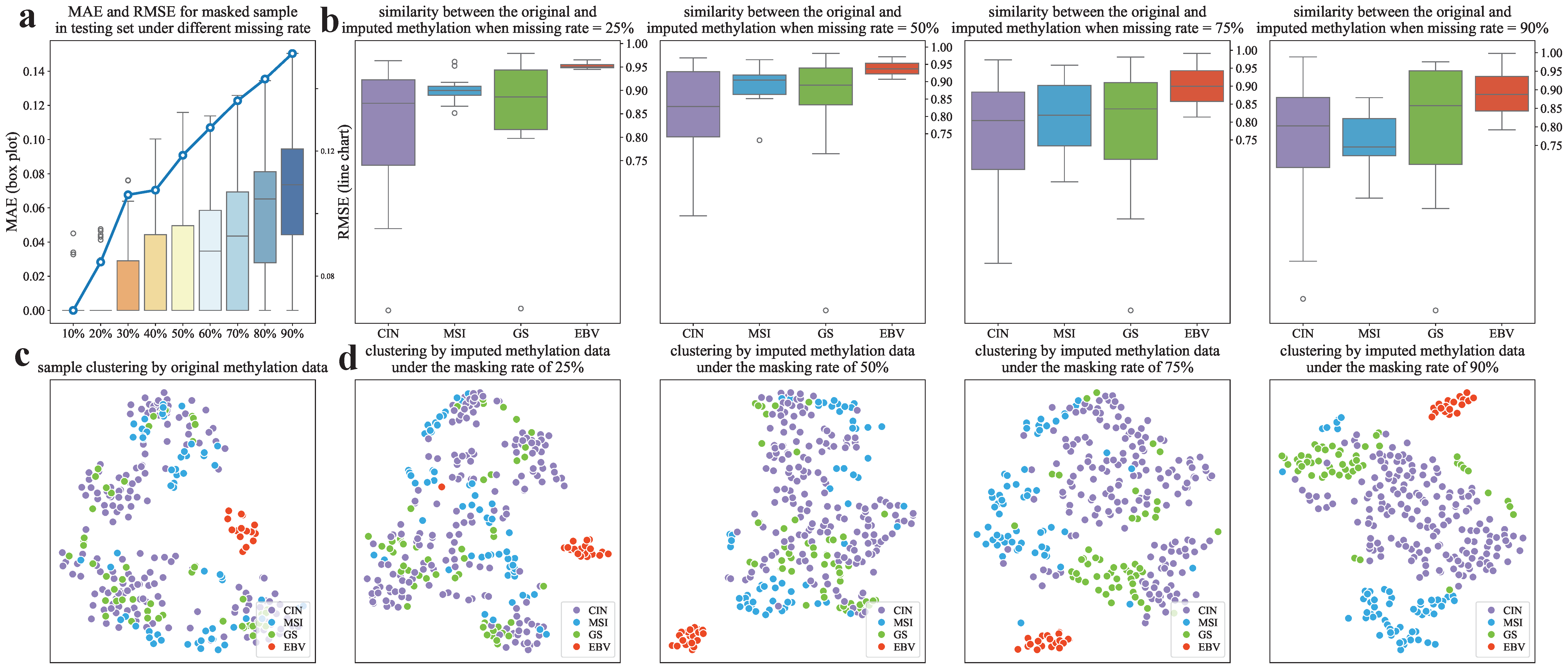

Supplement: S10 Fig — (a) Mean Absolute Error (MAE) between original and imputed methylation data in the testing set at different missing rates, and corresponding Root Mean Square Error (RMSE) at each rate.(b) Similarity between original and imputed methylation data under various missing rates across different subtypes.(c) Sample clustering using the original methylation data.(d) Sample clustering using the imputed methylation data under different missing rates. (TIF) [file pcbi.1012710.s010.tif]

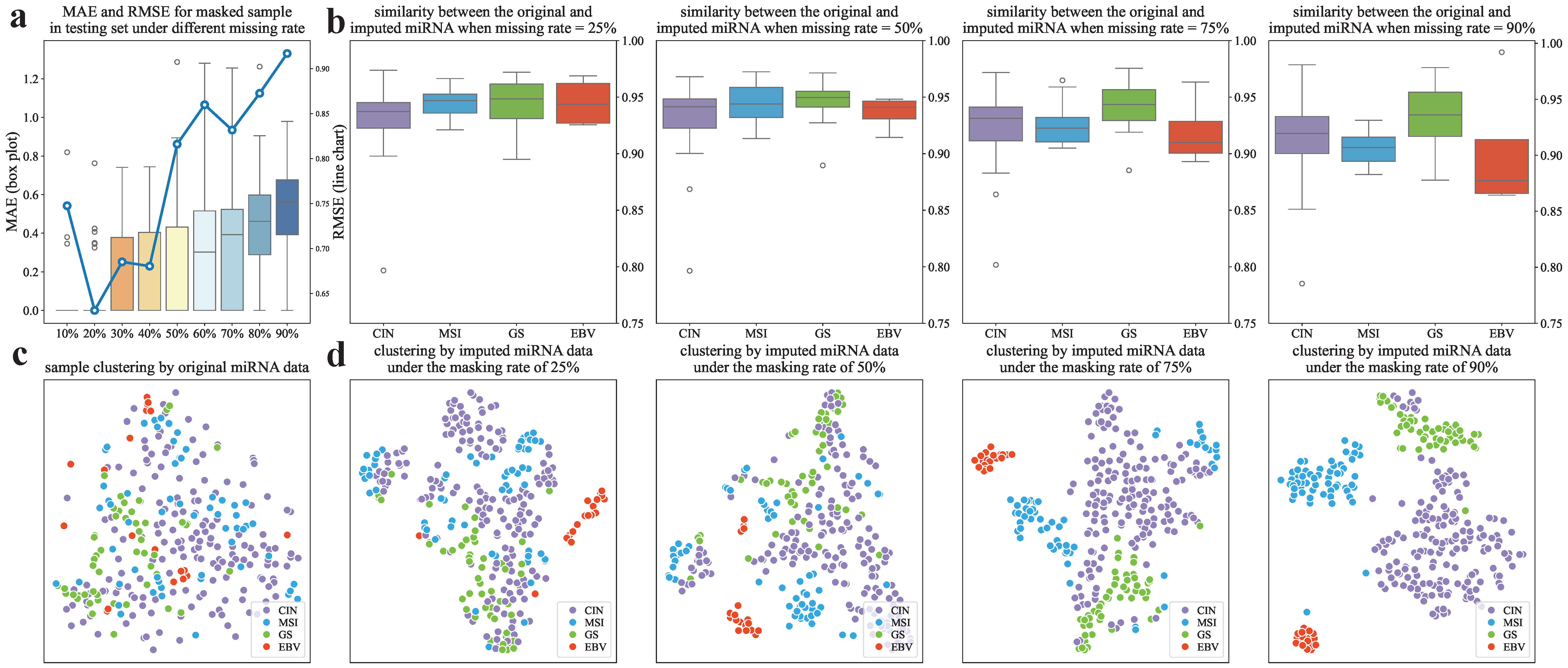

Supplement: S11 Fig — (a) Mean Absolute Error (MAE) between original and imputed miRNA data in the testing set at different missing rates, and corresponding Root Mean Square Error (RMSE) at each rate.(b) Similarity between original and imputed miRNA data under various missing rates across different subtypes.(c) Sample clustering using the original miRNA data.(d) Sample clustering using the imputed miRNA data under different missing rates. (TIF) [file pcbi.1012710.s011.tif]

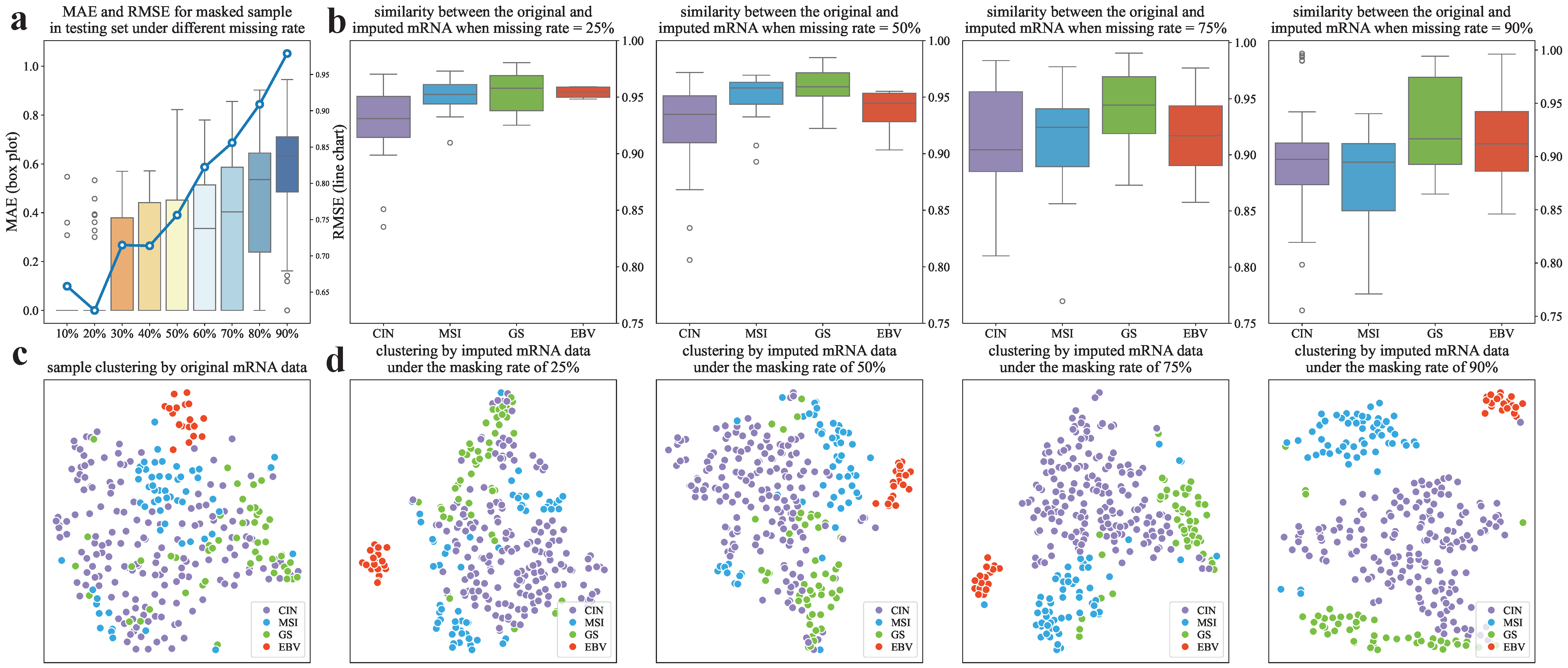

Supplement: S12 Fig — (a) Mean Absolute Error (MAE) between original and imputed mRNA data in the testing set at different missing rates, and corresponding Root Mean Square Error (RMSE) at each rate.(b) Similarity between original and imputed mRNA data under various missing rates across different subtypes.(c) Sample clustering using the original mRNA data.(d) Sample clustering using the imputed mRNA data under different missing rates. (TIF) [file pcbi.1012710.s012.tif]

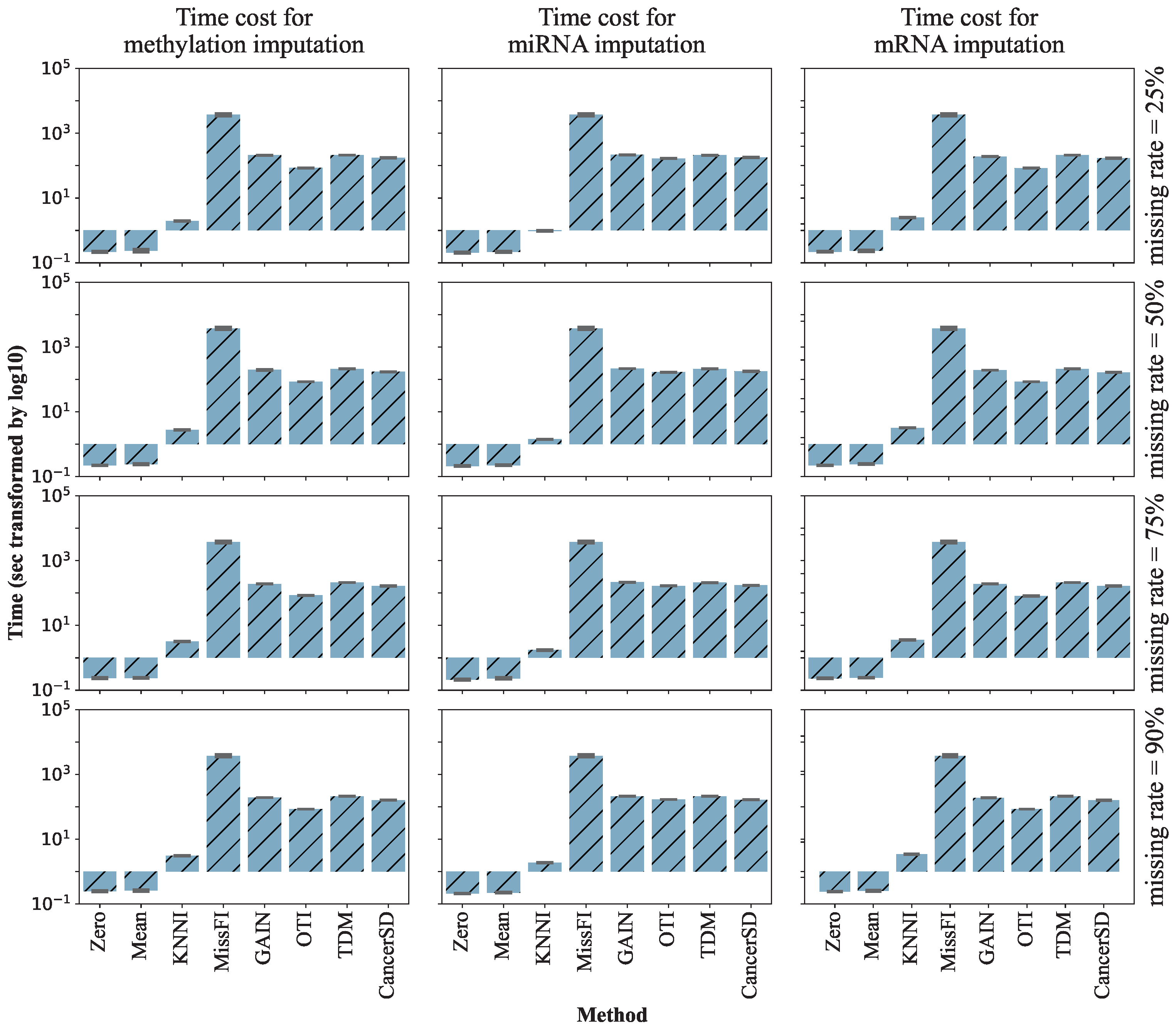

Supplement: S13 Fig — (TIF) [file pcbi.1012710.s013.tif]

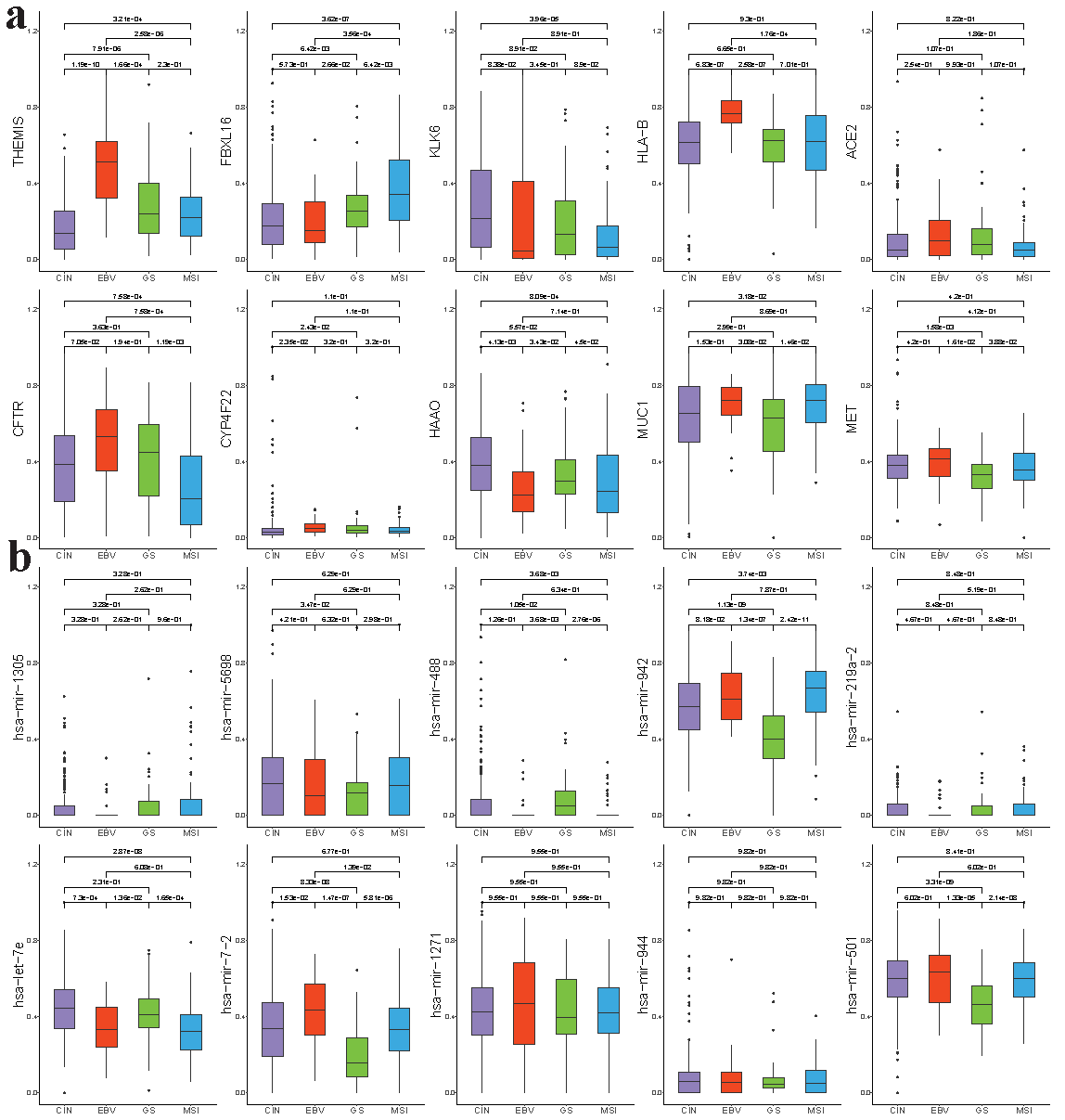

Supplement: S14 Fig — The expression values subjected to log2 transformation and normalization. Wilcoxon rank-sum test is employed to evaluate the differences in the expression levels of specific molecules among patients of distinct subtypes. (TIF) [file pcbi.1012710.s014.tif]

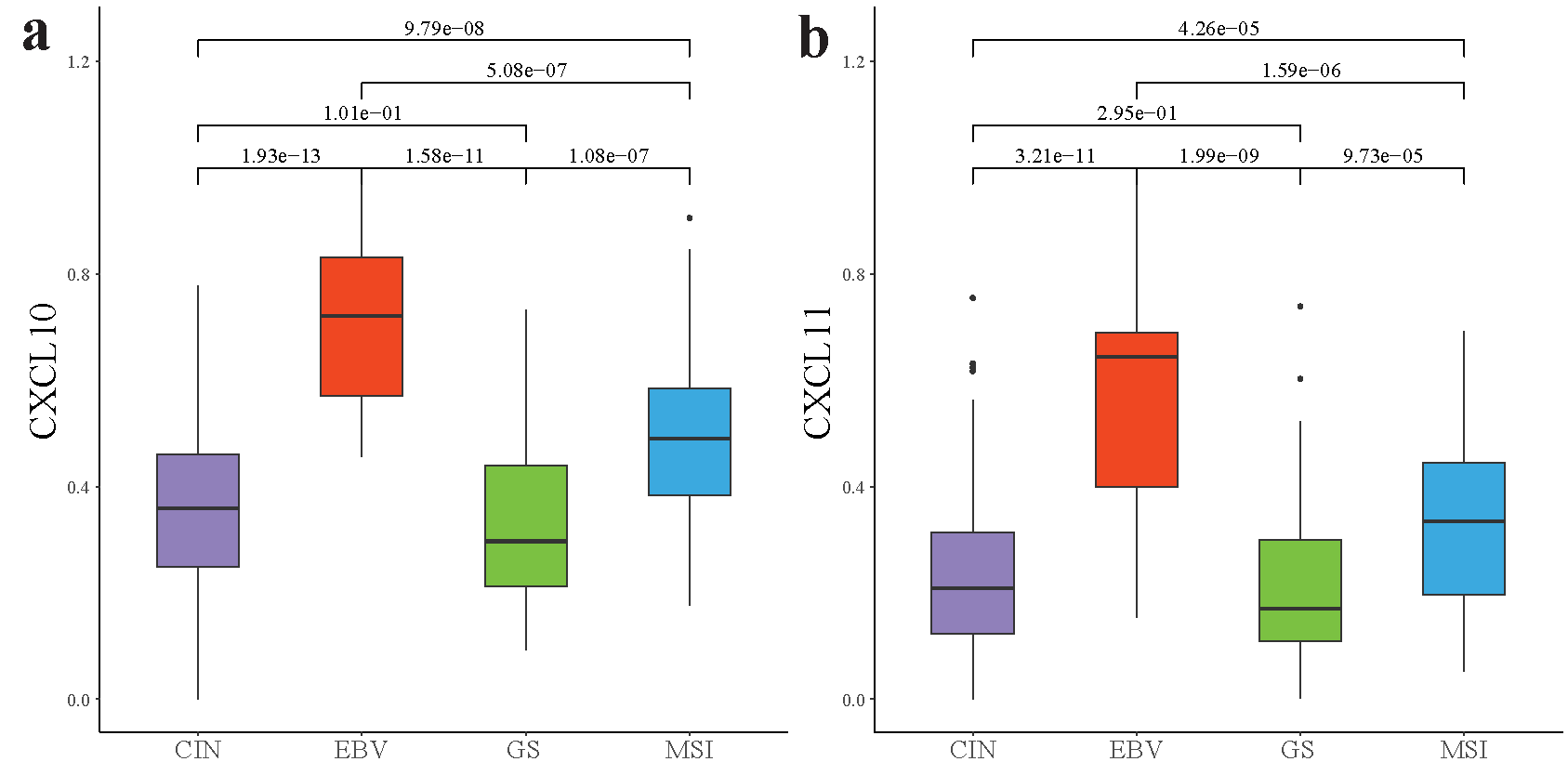

Supplement: S15 Fig — The expression values subjected to log2 transformation and normalization. Wilcoxon rank-sum test is employed to evaluate the differences in the expression levels of specific molecules among patients of distinct subtypes. (TIF) [file pcbi.1012710.s015.tif]

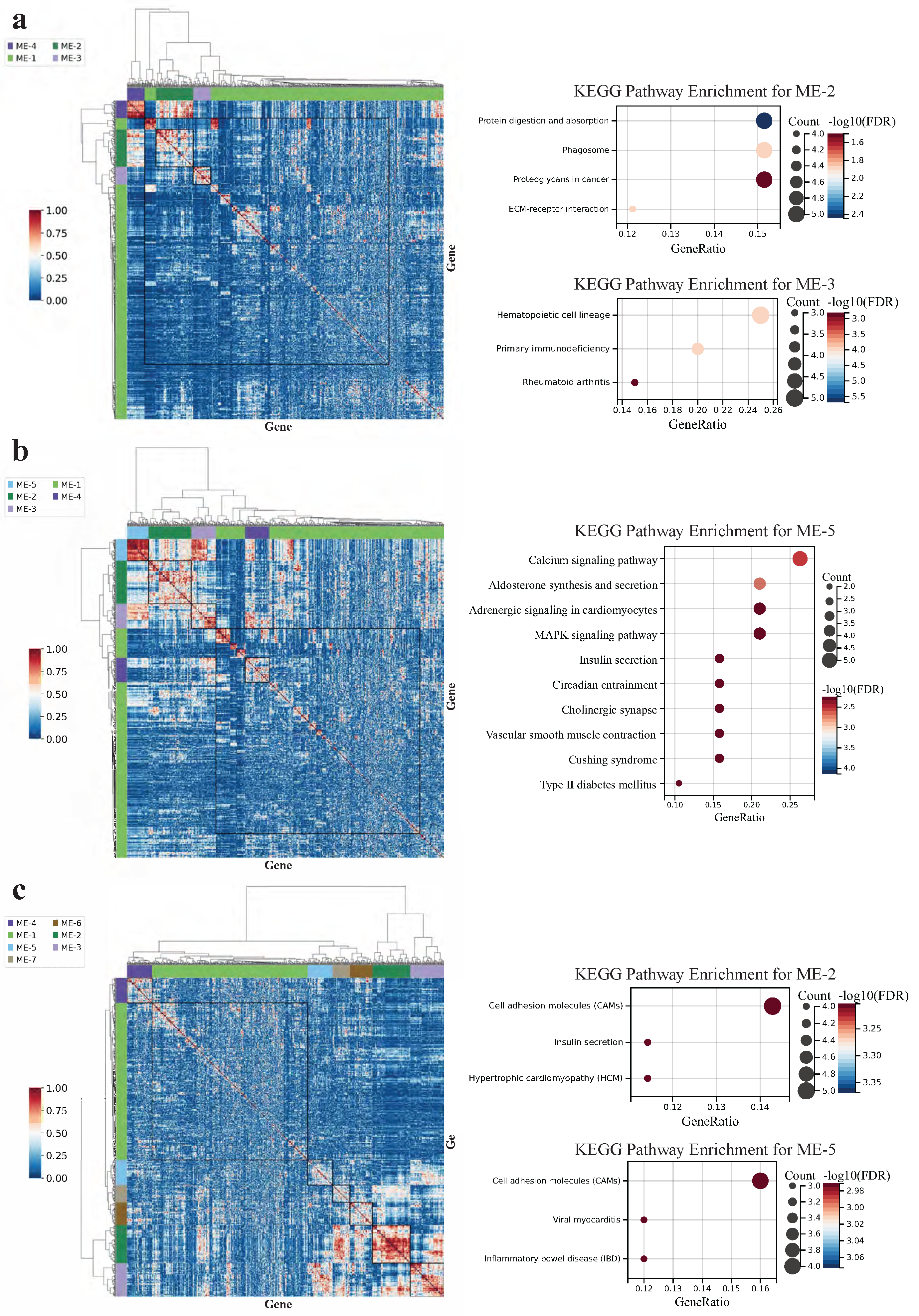

Supplement: S16 Fig — (TIF) [file pcbi.1012710.s016.tif]

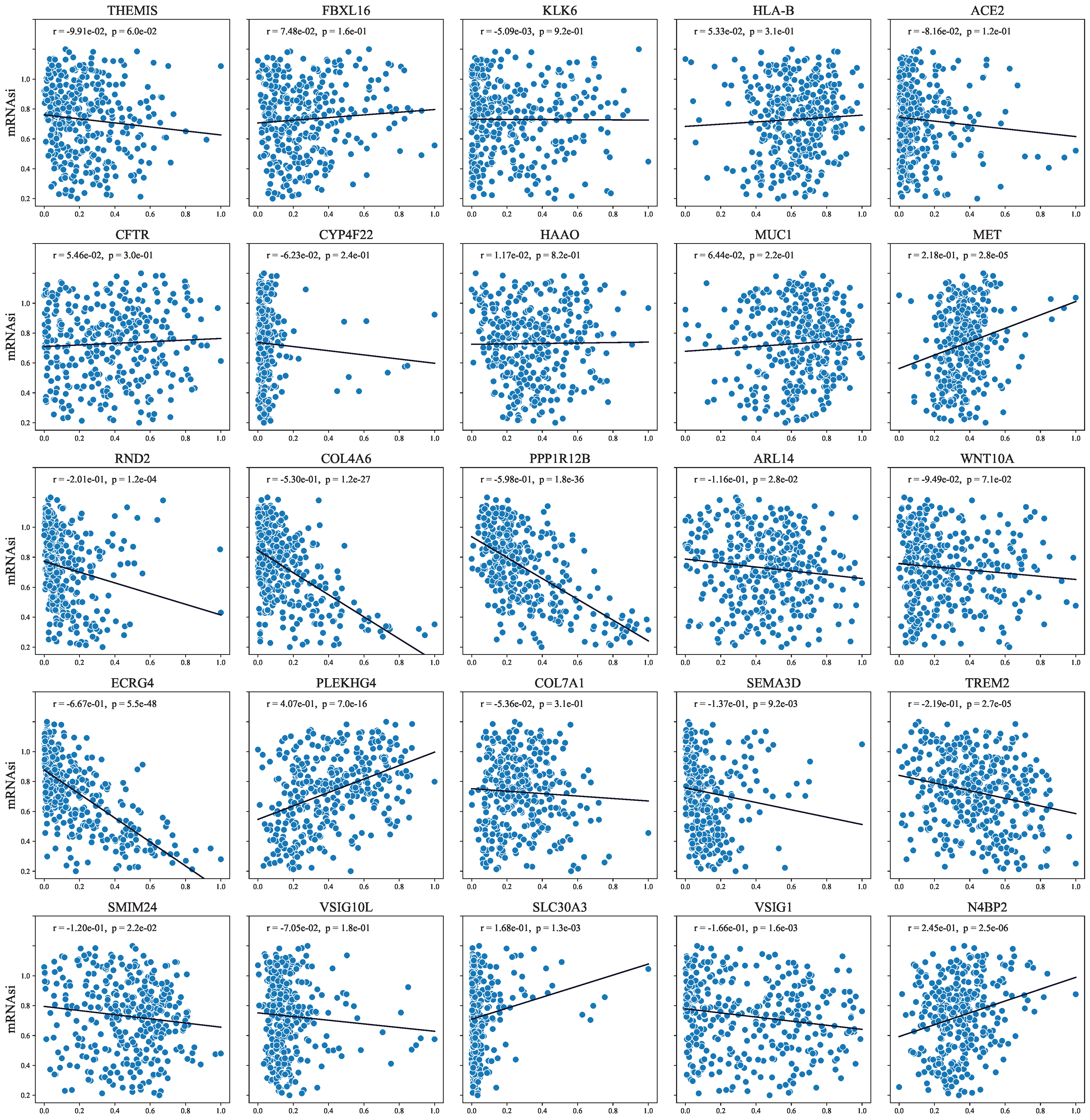

Supplement: S17 Fig — The regression lines in figures are fitted by the corresponding data. The significance in the figure is estimated by pearson correlation coefficient. (TIF) [file pcbi.1012710.s017.tif]
