# Supplementary material for: Cancer molecular subtyping using limited multi-omics data with missingness
Source: PLoS Comput Biol. 2024 Dec 26;20(12):e1012710. doi: 10.1371/journal.pcbi.1012710 (PMC11709273; doi:10.1371/journal.pcbi.1012710)
Supplement: S1 Text — Tables A-V. (PDF) [file pcbi.1012710.s018.pdf]

Supplementary Tables

Table A. Summary of cancer datasets.

Dataset	Cancer	Subtype	Number of features (meth, miRNA, mRNA)	Source
STAD	gastric	CIN: 230, MSI: 81, GS: 72, EBV: 32	485577, 1881, 19961	TCGA [1]
GSE62254	gastric	CIN: 66, MSI: 39, GS: 123, EBV: 14	-,-,20188	GEO [2]
ADSC	lung	LUAD: 518, LUSC: 503	485577, 1881, 19961	TCGA
CPTAC	lung	LUAD: 222, LUSC: 102	865918, 1881, 19961	CPTAC [3, 4]
BRCA	breast	Luminal A: 459, Luminal B: 168, Basal-like: 164, HER2-enriched: 66, Normal-like: 35	485577, 1881, 19961	TCGA

Note: meth refers to DNA methylation data, miRNA refers to miRNA expression data and mRNA refers to mRNA expression data. Due to the presence of only one major lung cancer subtype in each of the two lung cancer datasets (LUAD and LUSC) from the TCGA project, we merge the two datasets and refer to the combined dataset as ADSC.

Table B. Gastric cancer subtype diagnosis performance on the STAD dataset.

Category	Method	Accuracy	AUROC	Precision	F1-Score
Traditional ML	KNN	0.705±0.029 •	0.861±0.029 •	0.720±0.031 •	0.703±0.030 •
	RFC [5]	0.774±0.028 •	0.829±0.019 •	0.781±0.032 •	0.759±0.032 •
MI-base	AE-XGBoost [6]	0.812±0.025 •	0.943±0.017 •	0.817±0.027 •	0.806±0.031 •
	MOMA [7]	0.816±0.027 •	0.936±0.016 •	0.781±0.032 •	0.817±0.024 •
	MOGONET [8]	0.833±0.020 •	0.910±0.031 •	0.832±0.022 •	0.827±0.022 •
	MOFA+ [9]	0.807±0.016 •	0.921±0.011 •	0.824±0.025 •	0.819±0.020 •
	FactorCL [10]	0.831±0.024 •	0.921±0.019 •	0.839±0.028 •	0.824±0.023 •
	VICReg [11]	0.825±0.022 •	0.917±0.014 •	0.821±0.021 •	0.809±0.030 •
iMI-base	Subtype-GAN [12]	0.842±0.023 •	0.946±0.018	0.848±0.019 •	0.833±0.027 •
	scVAEIT [13]	0.855±0.016 •	0.943±0.013 •	0.857±0.012 •	0.849±0.021 •
	DCP [14]	0.852±0.018 •	0.948±0.017	0.853±0.019 •	0.847±0.019 •
	APADC [15]	0.846±0.018 •	0.953±0.016	0.853±0.014 •	0.837±0.023 •
	CancerSD	**0.887±0.018**	**0.960±0.014**	**0.889±0.016**	**0.884±0.019**

Note: The best results are shown in **bold** face. '•' indicates CancerSD performs significantly better than the other method. The statistical significance is assessed at the 95% level by student t-test.

Table C. Cancer subtype diagnosis performance on different cancer datasets.

Category	Method	Metric	GSE62254	ADSC (2-way 10-shot)	CPTAC (2-way 10-shot)	BRCA
Traditional ML	KNN	Accuracy	0.563±0.040 •	0.846±0.031 •	0.674±0.063 •	0.725±0.018 •
		AUROC	0.800±0.026 •	0.911±0.026 •	0.701±0.040 •	0.875±0.020 •
		Precision	0.562±0.049 •	0.834±0.052 •	0.520±0.157 •	0.748±0.051 •
		F1 Score	0.540±0.043 •	0.847±0.030 •	0.495±0.062 •	0.667±0.028 •
	RFC	Accuracy	0.631±0.036 •	0.875±0.028 •	0.718±0.037 •	0.828±0.012 •
		AUROC	0.836±0.016	0.946±0.017 •	0.778±0.067 •	0.959±0.008
		Precision	0.613±0.061 •	0.888±0.049 •	0.532±0.054 •	0.801±0.013 •
		F1 Score	0.597±0.041 •	0.869±0.042 •	0.565±0.078 •	0.804±0.013 •
MI-based	AE-XGBoost	Accuracy	0.624±0.031 •	0.887±0.049 •	0.732±0.019 •	0.845±0.017 •
		AUROC	0.808±0.032 •	0.889±0.051 •	0.790±0.044 •	0.950±0.017 •
		Precision	0.632±0.041 •	0.903±0.051	**0.804±0.139**	0.823±0.020 •
		F1 Score	0.618±0.033 •	0.882±0.052 •	0.329±0.081 •	0.826±0.017 •
	MOMA	Accuracy	0.643±0.032	0.874±0.032 •	0.721±0.031 •	0.840±0.022 •
		AUROC	0.818±0.024 •	0.954±0.015 •	0.791±0.038 •	0.955±0.014
		Precision	0.642±0.038 •	0.881±0.062 •	0.559±0.075 •	0.836±0.024 •
		F1 Score	0.633±0.037 •	0.871±0.038 •	0.536±0.132 •	0.834±0.023 •
	MOGONET	Accuracy	0.635±0.028 •	0.876±0.025 •	0.681±0.051 •	0.832±0.013 •
		AUROC	0.802±0.024 •	0.925±0.032 •	0.725±0.053 •	0.915±0.020 •
		Precision	0.641±0.029 •	0.906±0.034 •	0.495±0.089 •	0.825±0.028 •
		F1 Score	0.629±0.031 •	0.870±0.027 •	0.543±0.057 •	0.821±0.018 •
	MOFA+	Accuracy	0.629±0.034 •	0.882±0.027 •	0.707±0.033 •	0.838±0.019 •
		AUROC	0.812±0.029 •	0.912±0.030 •	0.761±0.048 •	0.935±0.018 •
		Precision	0.635±0.046 •	0.892±0.044 •	0.540±0.076 •	0.813±0.022 •
		F1 Score	0.621±0.035 •	0.879±0.036 •	0.532±0.097 •	0.811±0.020 •
	FactorCL	Accuracy	0.644±0.032	0.872±0.033 •	0.726±0.028 •	0.836±0.023 •
		AUROC	0.819±0.026 •	0.950±0.017 •	0.782±0.041 •	0.952±0.012 •
		Precision	0.629±0.039 •	0.879±0.060 •	0.541±0.088 •	0.831±0.027 •
		F1 Score	0.631±0.034 •	0.872±0.041 •	0.527±0.116 •	0.837±0.021 •
	VICReg	Accuracy	0.627±0.030 •	0.870±0.026 •	0.714±0.023 •	0.825±0.018 •
		AUROC	0.797±0.028 •	0.917±0.029 •	0.743±0.051 •	0.929±0.022 •
		Precision	0.631±0.035 •	0.902±0.037 •	0.482±0.081 •	0.818±0.025 •
		F1 Score	0.618±0.032 •	0.869±0.031 •	0.531±0.089 •	0.814±0.020 •
iMI-based	Subtype-GAN	Accuracy	0.643±0.037	0.891±0.020 •	0.737±0.029 •	0.849±0.016
		AUROC	0.822±0.029	0.957±0.008 •	0.801±0.033 •	0.944±0.013 •
		Precision	0.651±0.035	0.903±0.030 •	0.625±0.093	0.837±0.027 •
		F1 Score	0.637±0.033	0.894±0.023 •	0.541±0.075 •	0.843±0.018 •
	scVAEIT	Accuracy	0.657±0.035	0.918±0.022	0.754±0.030 •	0.851±0.017
		AUROC	0.837±0.027	0.969±0.010 •	0.827±0.036	0.958±0.009
		Precision	0.655±0.036	0.922±0.032	0.657±0.100	0.844±0.017 •
		F1 Score	0.640±0.034	0.910±0.025 •	0.573±0.080 •	0.834±0.019 •
	DCP	Accuracy	0.659±0.035	0.911±0.014 •	0.759±0.028	0.855±0.015
		AUROC	0.846±0.021	0.961±0.019 •	0.757±0.042 •	0.941±0.017 •
		Precision	0.671±0.039	0.919±0.034	0.680±0.110	0.843±0.026
		F1 Score	0.655±0.038	0.910±0.013 •	0.567±0.081 •	0.842±0.018 •
	APADC	Accuracy	0.645±0.023	0.904±0.024 •	0.751±0.033 •	0.857±0.012 •
		AUROC	0.840±0.024	0.975±0.010	0.830±0.044	0.951±0.011 •
		Precision	0.645±0.022 •	0.913±0.056	0.658±0.156	0.849±0.012
		F1 Score	0.641±0.024	0.901±0.027 •	0.552±0.116 •	0.849±0.012 •
	CancerSD	Accuracy	**0.676±0.046**	**0.935±0.013**	**0.784±0.032**	**0.862±0.015**
		AUROC	**0.848±0.030**	**0.979±0.007**	**0.858±0.034**	**0.965±0.007**
		Precision	**0.683±0.048**	**0.939±0.021**	0.655±0.094	**0.862±0.014**
		F1 Score	**0.673±0.046**	**0.934±0.014**	**0.656±0.046**	**0.859±0.014**

Note: The best results are shown in **bold** face. '•' indicates CancerSD performs significantly better than the other method. The statistical significance is assessed at the 95% level by student t-test.

Table D. Gastric cancer subtype diagnosis performance on STAD dataset under different omics missing rates.

Category	Method	Metric	Missing Rate			
			0%	25%	50%	75%
Traditional ML	KNN	Accuracy	0.754±0.035 •	0.670±0.045 •	0.641±0.024 •	0.607±0.021 •
		AUROC	0.910±0.019 •	0.853±0.022 •	0.827±0.035 •	0.797±0.036 •
		Precision	0.768±0.045 •	0.672±0.075 •	0.652±0.069 •	0.539±0.074 •
		F1 Score	0.732±0.044 •	0.637±0.055 •	0.557±0.038 •	0.496±0.033 •
	RFC	Accuracy	0.824±0.019 •	0.780±0.035 •	0.757±0.035 •	0.718±0.037 •
		AUROC	0.947±0.015	0.927±0.018 •	0.915±0.018 •	0.885±0.027 •
		Precision	0.835±0.023 •	0.795±0.043 •	0.784±0.039 •	0.744±0.053 •
		F1 Score	0.810±0.024 •	0.763±0.038 •	0.732±0.039 •	0.680±0.044 •
MI-based	AE-XGBoost	Accuracy	0.845±0.028 •	0.800±0.037 •	0.798±0.021 •	0.757±0.030 •
		AUROC	0.958±0.014	0.938±0.017	0.933±0.014	0.886±0.035 •
		Precision	0.849±0.028 •	0.805±0.044 •	0.806±0.025 •	0.755±0.031 •
		F1 Score	0.837±0.030 •	0.789±0.037 •	0.786±0.022 •	0.740±0.033 •
	MOMA	Accuracy	0.841±0.045 •	0.815±0.048 •	0.793±0.048 •	0.744±0.046 •
		AUROC	0.955±0.017	0.941±0.014	0.919±0.011 •	0.892±0.022 •
		Precision	0.849±0.050 •	0.824±0.045 •	0.802±0.051 •	0.751±0.052 •
		F1 Score	0.837±0.048 •	0.812±0.048 •	0.786±0.047 •	0.734±0.044 •
	MOGONET	Accuracy	0.854±0.024 •	0.816±0.024 •	0.777±0.033 •	0.715±0.027 •
		AUROC	0.920±0.024 •	0.919±0.027 •	0.909±0.026 •	0.861±0.029 •
		Precision	0.859±0.027 •	0.818±0.031 •	0.782±0.030 •	0.704±0.055 •
		F1 Score	0.851±0.024 •	0.807±0.026 •	0.761±0.042 •	0.690±0.043 •
iMI-based	DCP	Accuracy	0.854±0.015 •	0.848±0.023 •	0.821±0.017 •	0.771±0.030 •
		AUROC	0.958±0.013	0.935±0.018	0.901±0.027 •	0.898±0.033 •
		Precision	0.861±0.019 •	0.852±0.027	0.828±0.014 •	0.810±0.023
		F1 Score	0.855±0.015 •	0.844±0.023 •	0.820±0.015 •	0.778±0.027 •
	APADC	Accuracy	0.866±0.023 •	0.830±0.033 •	0.823±0.026 •	0.792±0.021 •
		AUROC	**0.959±0.012**	0.934±0.012	0.914±0.012 •	0.885±0.022 •
		Precision	0.871±0.025	0.830±0.060	0.837±0.026 •	0.814±0.029
		F1 Score	0.864±0.022 •	0.819±0.043 •	0.816±0.031 •	0.781±0.024 •
	CancerSD	Accuracy	**0.895±0.031**	**0.869±0.019**	**0.862±0.023**	**0.821±0.025**
		AUROC	0.955±0.016	**0.947±0.020**	**0.942±0.015**	**0.925±0.018**
		Precision	**0.898±0.033**	**0.873±0.022**	**0.869±0.021**	**0.831±0.029**
		F1 Score	**0.894±0.032**	**0.866±0.020**	**0.861±0.022**	**0.812±0.027**

Note: The best results are shown in **bold** face. '•' indicates CancerSD performs significantly better than the other method. The statistical significance is assessed at the 95% level by student t-test.

Table E. Gastric cancer subtype diagnosis performance on STAD dataset under different missing rates (missingness occurring on methylation).

Category	Method	Metric	Missing Rate			
			25%	50%	75%	90%
Traditional ML	KNN	Accuracy	0.746±0.018 •	0.715±0.042 •	0.736±0.040 •	0.702±0.037 •
		AUROC	0.885±0.016 •	0.899±0.024 •	0.878±0.020 •	0.869±0.018 •
		Precision	0.748±0.023 •	0.720±0.055 •	0.736±0.046 •	0.715±0.037 •
		F1 Score	0.741±0.020 •	0.694±0.045 •	0.730±0.041 •	0.702±0.032 •
	RFC	Accuracy	0.781±0.029 •	0.782±0.034 •	0.761±0.034 •	0.739±0.034 •
		AUROC	0.942±0.019	0.936±0.019	0.927±0.016	0.915±0.025
		Precision	0.794±0.039 •	0.806±0.032 •	0.779±0.034 •	0.754±0.057 •
		F1 Score	0.769±0.032 •	0.766±0.040 •	0.741±0.042 •	0.717±0.041 •
MI-based	AE-XGBoost	Accuracy	0.805±0.040 •	0.805±0.033 •	0.787±0.045 •	0.759±0.025 •
		AUROC	0.925±0.029	0.925±0.027 •	0.908±0.034 •	0.859±0.032 •
		Precision	0.805±0.038 •	0.805±0.036 •	0.796±0.051 •	0.762±0.026 •
		F1 Score	0.799±0.042 •	0.798±0.036 •	0.781±0.045 •	0.746±0.025 •
	MOMA	Accuracy	0.828±0.038 •	0.815±0.031 •	0.782±0.046 •	0.766±0.031 •
		AUROC	0.924±0.019 •	0.918±0.017 •	0.897±0.035 •	0.899±0.031 •
		Precision	0.836±0.043 •	0.827±0.025 •	0.793±0.041 •	0.771±0.026 •
		F1 Score	0.825±0.038 •	0.811±0.029 •	0.776±0.045 •	0.758±0.032 •
	MOGONET	Accuracy	0.834±0.036 •	0.816±0.020 •	0.797±0.022 •	0.769±0.025 •
		AUROC	0.924±0.027 •	0.903±0.027 •	0.905±0.029 •	0.869±0.036 •
		Precision	0.840±0.035 •	0.825±0.051 •	0.809±0.031 •	0.721±0.083 •
		F1 Score	0.825±0.042 •	0.791±0.029 •	0.782±0.024 •	0.711±0.036 •
iMI-based	DCP	Accuracy	0.849±0.024 •	0.825±0.027 •	0.808±0.031 •	0.784±0.022 •
		AUROC	0.946±0.009	0.934±0.020	0.868±0.047 •	0.859±0.033 •
		Precision	0.854±0.026 •	0.844±0.027 •	0.809±0.043 •	0.801±0.035 •
		F1 Score	0.846±0.024 •	0.829±0.026 •	0.799±0.036 •	0.782±0.027 •
	APADC	Accuracy	0.851±0.026 •	0.848±0.027	0.820±0.026 •	0.813±0.029 •
		AUROC	0.940±0.012	0.940±0.016	0.918±0.028	0.930±0.028
		Precision	0.859±0.026 •	0.857±0.024	0.833±0.027 •	0.845±0.032
		F1 Score	0.847±0.026 •	0.845±0.026 •	0.808±0.032 •	0.820±0.028
	CancerSD	Accuracy	**0.887±0.026**	**0.872±0.027**	**0.852±0.026**	**0.848±0.030**
		AUROC	**0.949±0.022**	**0.950±0.024**	**0.940±0.028**	**0.939±0.037**
		Precision	**0.891±0.027**	**0.879±0.027**	**0.858±0.025**	**0.859±0.028**
		F1 Score	**0.885±0.026**	**0.871±0.026**	**0.851±0.024**	**0.845±0.030**

Note: The best results are shown in **bold** face. '•' indicates CancerSD performs significantly better than the other method. The statistical significance is assessed at the 95% level by student t-test.

Table F. Gastric cancer subtype diagnosis performance on STAD dataset under different missing rates (missingness occurring on miRNA).

Category	Method	Metric	Missing Rate 25%	50%	75%	90%
Traditional ML	KNN	Accuracy	0.754±0.029 •	0.733±0.043 •	0.746±0.028 •	0.726±0.034 •
		AUROC	0.886±0.015 •	0.886±0.016 •	0.889±0.014 •	0.899±0.022 •
		Precision	0.750±0.031 •	0.732±0.045 •	0.740±0.031 •	0.742±0.048 •
		F1 Score	0.746±0.032 •	0.725±0.047 •	0.737±0.026 •	0.691±0.047 •
	RFC	Accuracy	0.815±0.023 •	0.815±0.025 •	0.797±0.045 •	0.777±0.036 •
		AUROC	0.945±0.014	0.946±0.014	0.929±0.024	0.898±0.023 •
		Precision	0.822±0.028 •	0.822±0.028 •	0.810±0.047 •	0.771±0.041 •
		F1 Score	0.800±0.027 •	0.796±0.031 •	0.777±0.055 •	0.759±0.039 •
MI-based	AE-XGBoost	Accuracy	0.830±0.035 •	0.820±0.031 •	0.816±0.035 •	0.801±0.039 •
		AUROC	0.941±0.024	0.944±0.025	0.939±0.028	0.937±0.029
		Precision	0.821±0.041 •	0.815±0.030 •	0.809±0.040 •	0.796±0.036 •
		F1 Score	0.816±0.044 •	0.809±0.035 •	0.804±0.044 •	0.791±0.042 •
	MOMA	Accuracy	0.830±0.035 •	0.820±0.028 •	0.811±0.030 •	0.791±0.031 •
		AUROC	0.896±0.026 •	0.906±0.017 •	0.930±0.014	0.933±0.009
		Precision	0.830±0.039 •	0.824±0.031 •	0.807±0.048 •	0.723±0.071 •
		F1 Score	0.822±0.037 •	0.811±0.031 •	0.787±0.034 •	0.745±0.054 •
	MOGONET	Accuracy	0.825±0.018 •	0.819±0.019 •	0.808±0.015 •	0.793±0.022 •
		AUROC	0.920±0.030 •	0.911±0.042 •	0.918±0.025 •	0.902±0.032 •
		Precision	0.821±0.028 •	0.826±0.020 •	0.801±0.019 •	0.734±0.060 •
		F1 Score	0.801±0.025 •	0.793±0.035 •	0.796±0.019 •	0.746±0.040 •
iMI-based	DCP	Accuracy	0.854±0.021 •	0.834±0.026 •	0.825±0.023 •	0.808±0.037 •
		AUROC	0.942±0.016	0.932±0.027	0.912±0.031 •	0.881±0.038 •
		Precision	0.859±0.026	0.847±0.030 •	0.831±0.028 •	0.818±0.033 •
		F1 Score	0.846±0.027 •	0.830±0.029 •	0.820±0.028 •	0.796±0.036 •
	APADC	Accuracy	0.862±0.023	0.833±0.016 •	0.828±0.030 •	0.826±0.029 •
		AUROC	0.927±0.025 •	0.936±0.028	0.939±0.020	0.922±0.021
		Precision	0.873±0.018	0.852±0.023 •	0.840±0.033	0.837±0.033
		F1 Score	0.860±0.022	0.821±0.015 •	0.819±0.035 •	0.814±0.032 •
	CancerSD	Accuracy	**0.882±0.028**	**0.875±0.032**	**0.862±0.032**	**0.854±0.021**
		AUROC	**0.953±0.019**	**0.952±0.020**	**0.943±0.015**	**0.938±0.017**
		Precision	**0.885±0.031**	**0.881±0.033**	**0.867±0.030**	**0.857±0.025**
		F1 Score	**0.881±0.029**	**0.875±0.031**	**0.860±0.031**	**0.849±0.021**

Note: The best results are shown in **bold** face. '•' indicates CancerSD performs significantly better than the other method. The statistical significance is assessed at the 95% level by student t-test.

Table G. Gastric cancer subtype diagnosis performance on STAD dataset under different missing rates (missingness occurring on mRNA).

Category	Method	Metric	Missing Rate			
			25%	50%	75%	90%
Traditional ML	KNN	Accuracy	0.715±0.027 •	0.645±0.034 •	0.670±0.026 •	0.647±0.022 •
		AUROC	0.855±0.024 •	0.865±0.017 •	0.856±0.027 •	0.858±0.031 •
		Precision	0.720±0.030 •	0.627±0.070 •	0.643±0.056 •	0.605±0.094 •
		F1 Score	0.706±0.030 •	0.579±0.054 •	0.603±0.037 •	0.558±0.038 •
	RFC	Accuracy	0.800±0.026 •	0.790±0.026 •	0.774±0.039 •	0.734±0.044 •
		AUROC	0.939±0.016	0.933±0.021	0.887±0.027 •	0.865±0.027 •
		Precision	0.807±0.056 •	0.812±0.033 •	0.785±0.045 •	0.730±0.059 •
		F1 Score	0.773±0.037 •	0.763±0.341	0.750±0.044 •	0.710±0.055 •
MI-based	AE-XGBoost	Accuracy	0.811±0.035 •	0.805±0.039 •	0.793±0.035 •	0.762±0.042 •
		AUROC	0.943±0.023	0.938±0.026	0.928±0.020	0.895±0.024 •
		Precision	0.810±0.037 •	0.802±0.041 •	0.792±0.040 •	0.758±0.046 •
		F1 Score	0.804±0.036 •	0.793±0.042 •	0.780±0.035 •	0.752±0.046 •
	MOMA	Accuracy	0.821±0.031 •	0.807±0.030 •	0.795±0.036 •	0.777±0.038 •
		AUROC	0.915±0.024 •	0.881±0.019 •	0.878±0.038 •	0.870±0.035 •
		Precision	0.800±0.058 •	0.828±0.027 •	0.800±0.043 •	0.798±0.043 •
		F1 Score	0.797±0.042 •	0.805±0.030 •	0.754±0.040 •	0.769±0.044 •
	MOGONET	Accuracy	0.823±0.012 •	0.792±0.022 •	0.789±0.024 •	0.766±0.024 •
		AUROC	0.911±0.029 •	0.910±0.030 •	0.907±0.025 •	0.897±0.037 •
		Precision	0.839±0.020 •	0.766±0.062 •	0.800±0.042 •	0.765±0.031 •
		F1 Score	0.804±0.020 •	0.757±0.037 •	0.772±0.028 •	0.738±0.027 •
iMI-based	DCP	Accuracy	0.851±0.030 •	0.834±0.026 •	0.810±0.038 •	0.798±0.027 •
		AUROC	0.942±0.016	0.932±0.027	0.910±0.034 •	0.858±0.038 •
		Precision	0.861±0.032	0.847±0.030 •	0.831±0.031 •	0.813±0.030 •
		F1 Score	0.849±0.031 •	0.830±0.029 •	0.810±0.040 •	0.796±0.027 •
	APADC	Accuracy	0.852±0.019 •	0.833±0.022 •	0.833±0.027	0.813±0.017 •
		AUROC	0.939±0.022	0.923±0.027 •	0.918±0.019 •	0.895±0.020 •
		Precision	0.862±0.022 •	0.842±0.029 •	0.841±0.026	0.828±0.020
		F1 Score	0.847±0.021 •	0.824±0.027 •	0.823±0.028 •	0.800±0.022 •
	CancerSD	Accuracy	**0.885±0.030**	**0.874±0.029**	**0.854±0.030**	**0.847±0.032**
		AUROC	**0.950±0.020**	**0.949±0.015**	**0.939±0.021**	**0.933±0.021**
		Precision	**0.889±0.030**	**0.880±0.026**	**0.862±0.030**	**0.851±0.035**
		F1 Score	**0.885±0.030**	**0.874±0.028**	**0.851±0.029**	**0.842±0.032**

Note: The best results are shown in **bold** face. '•' indicates CancerSD performs significantly better than the other method. The statistical significance is assessed at the 95% level by student t-test.

Table H. Cancer subtype diagnosis performance of CancerSD and its variants on the STAD dataset.

Variant	Accuracy	AUROC	Precision	F1-Score
CancerSD-FC	0.855±0.029 •	0.956±0.012	0.861±0.027 •	0.854±0.029 •
CancerSD-w/oGen	0.867±0.018 •	0.956±0.015	0.869±0.017 •	0.864±0.019 •
CancerSD-w/oCon	0.849±0.039 •	0.945±0.015 •	0.853±0.037 •	0.846±0.039 •
CancerSD-w/oPrj	0.830±0.033 •	0.920±0.025 •	0.836±0.032 •	0.825±0.034 •
CancerSD	**0.887±0.018**	**0.960±0.014**	**0.889±0.016**	**0.884±0.019**

Note: The best results are shown in **bold** face. '•' indicates CancerSD performs significantly better than the other method. The statistical significance is assessed at the 95% level by student t-test.

Table I. The diagnostic performance of different methods on the GSE62254 dataset under few-sample scenario.

Method	Accuracy	AUROC	Precision	F1 Score
kNN	0.475±0.078	0.780±0.024	0.653±0.036	0.486±0.080
RFC	0.527±0.035	0.806±0.023	0.624±0.031	0.540±0.036
AE-XGBoost	0.536±0.045	0.813±0.024	0.629±0.038	0.546±0.046
MOMA	0.525±0.057	0.835±0.017	**0.660±0.046**	0.531±0.059
MOGONET	0.537±0.046	0.780±0.031	0.629±0.034	0.545±0.046
DCP	0.537±0.033	0.813±0.021	0.652±0.035	0.537±0.051
APADC	0.552±0.041	0.834±0.017	0.646±0.039	0.563±0.039
CancerSD	**0.564±0.041**	**0.836±0.018**	0.657±0.031	**0.574±0.040**

Note: The "few-sample scenario" refers to to training the model using only a 4-way 10-shot subset of data from the GSE62254 dataset, without allowing access to any external datasets. The best results are shown in **bold** face.

Table J. The comparison of knowledge transfer performance across different gastric cancer datasets (multi2mRNA).

Method	Accuracy	AUROC	Precision	F1 Score
kNN	0.470±0.078 •	0.780±0.024 •	0.646±0.032 •	0.479±0.081 •
RFC [5]	0.570±0.022 •	0.820±0.014 •	0.681±0.020 •	0.566±0.024 •
DeepBDC [16]	0.625±0.028 •	0.866±0.011	0.706±0.019 •	0.632±0.027 •
QSFormer [17]	0.634±0.014 •	0.853±0.009 •	**0.729±0.022**	0.631±0.016 •
MOMA[7]-PT	0.591±0.025 •	0.845±0.009 •	0.691±0.028 •	0.593±0.027 •
DCP[14]-PT	0.596±0.027 •	0.806±0.038 •	0.535±0.140 •	0.540±0.069 •
CancerSD-PT	0.596±0.031 •	0.853±0.013 •	0.670±0.025 •	0.601±0.032 •
CancerSD-MOMA	0.617±0.029 •	0.850±0.008 •	0.703±0.017 •	0.620±0.031 •
CancerSD-DCP	0.606±0.025 •	0.794±0.021 •	0.711±0.023	0.601±0.030 •
CancerSD-SIM	0.628±0.018 •	0.866±0.011	0.700±0.019 •	0.631±0.018 •
CancerSD-w/oCLC	0.631±0.023 •	0.862±0.010	0.697±0.019 •	0.638±0.022 •
CancerSD	**0.679±0.022**	**0.868±0.008**	**0.729±0.025**	**0.682±0.021**

Note: "multi2mRNA" denotes that transferring knowledge from multi-omics data in STAD to support the optimization of mRNA-based diagnostic models in GSE62254. The best results are shown in **bold** face. '•' indicates CancerSD performs significantly better than the other method. The statistical significance is assessed at the 95% level by student t-test.

Table K. The comparison of knowledge transfer performance across different gastric cancer datasets (mRNA2mRNA).

Method	Accuracy	AUROC	Precision	F1 Score
kNN	0.467±0.077 •	0.778±0.022 •	0.641±0.034 •	0.475±0.081 •
RFC	0.562±0.036 •	0.830±0.014	0.696±0.026 •	0.550±0.044 •
DeepBDC	0.642±0.024 •	**0.867±0.009**	0.721±0.026	0.647±0.025 •
QSFormer	0.638±0.020 •	0.829±0.019	0.730±0.012	0.636±0.021 •
MOMA-PT	0.619±0.022 •	0.854±0.006	0.706±0.019 •	0.622±0.024 •
DCP-PT	0.590±0.046 •	0.836±0.013	0.677±0.042 •	0.593±0.044 •
CancerSD-PT	0.622±0.023 •	0.851±0.010	0.695±0.029 •	0.629±0.021 •
CancerSD-MOMA	0.627±0.025 •	0.852±0.013	0.715±0.030	0.632±0.026 •
CancerSD-DCP	0.608±0.037 •	0.829±0.017	0.665±0.020 •	0.608±0.036 •
CancerSD-SIM	0.628±0.022 •	0.854±0.004	0.705±0.017 •	0.633±0.020 •
CancerSD-w/oCLC	0.641±0.023 •	0.856±0.007	0.707±0.019 •	0.646±0.024 •
CancerSD	**0.675±0.016**	0.837±0.007	**0.733±0.022**	**0.676±0.017**

Note: "mRNA2mRNA" denotes that transferring knowledge from mRNA data in STAD to support the optimization of mRNA-based diagnostic models in GSE62254. The best results are shown in **bold** face. '•' indicates CancerSD performs significantly better than the other method. The statistical significance is assessed at the 95% level by student t-test.

Table L. Cancer subtype diagnosis performance of CancerSD on various datasets under different omics types.

Omics Type	Metric	STAD	ADSC	BRCA
DNA methylation (a) (independent learning)	Accuracy	0.802±0.028 ●	0.760±0.051 ●	0.652±0.052 ●
	AUROC	0.943±0.012	0.850±0.050 ●	0.866±0.021 ●
	Precision	0.820±0.029 ●	0.749±0.048 ●	0.713±0.036 ●
	F1 Score	0.805±0.027 ●	0.749±0.065 ●	0.658±0.045 ●
miRNA expression (b) (independent learning)	Accuracy	0.772±0.041 ●	0.898±0.036 ●	0.739±0.038 ●
	AUROC	0.929±0.024 ●	**0.987±0.007**	0.917±0.015 ●
	Precision	0.805±0.031 ●	0.937±0.070	0.781±0.028 ●
	F1 Score	0.776±0.040 ●	0.887±0.047 ●	0.740±0.031 ●
mRNA expression (c) (independent learning)	Accuracy	0.821±0.036 ●	0.844±0.063 ●	0.815±0.027 ●
	AUROC	0.941±0.018	0.944±0.024 ●	0.961±0.010
	Precision	0.847±0.028 ●	0.814±0.097 ●	0.851±0.022
	F1 Score	0.827±0.033 ●	0.851±0.049 ●	0.821±0.022 ●
DNA methylation (a) (joint learning)	Accuracy	0.844±0.040 ●	0.748±0.050 ●	0.694±0.027 ●
	AUROC	0.939±0.010 ●	0.844±0.055 ●	0.885±0.013 ●
	Precision	0.852±0.041 ●	0.727±0.058 ●	0.725±0.027 ●
	F1 Score	0.843±0.069	0.744±0.064 ●	0.703±0.024 ●
miRNA expression (b) (joint learning)	Accuracy	0.787±0.040 ●	0.916±0.045	0.784±0.028 ●
	AUROC	0.916±0.030 ●	0.983±0.007	0.939±0.012 ●
	Precision	0.800±0.034 ●	0.926±0.090	0.806±0.033 ●
	F1 Score	0.786±0.039 ●	0.914±0.039	0.790±0.028 ●
mRNA expression (c) (joint learning)	Accuracy	0.864±0.028 ●	0.907±0.024 ●	0.842±0.024
	AUROC	0.947±0.025	0.955±0.022 ●	**0.966±0.010**
	Precision	0.864±0.028 ●	0.892±0.062	0.858±0.019
	F1 Score	0.867±0.031	0.906±0.021 ●	0.847±0.022
Multi-omics (a+b)	Accuracy	0.857±0.028 ●	0.897±0.031 ●	0.809±0.030 ●
	AUROC	0.950±0.012	0.964±0.022 ●	0.935±0.014 ●
	Precision	0.864±0.034 ●	0.881±0.046 ●	0.809±0.032 ●
	F1 Score	0.854±0.031 ●	0.895±0.033 ●	0.804±0.031 ●
Multi-omics (a+c)	Accuracy	0.869±0.027	0.889±0.032 ●	0.841±0.030
	AUROC	0.956±0.016	0.959±0.015 ●	0.958±0.010
	Precision	0.879±0.030	0.878±0.058 ●	0.843±0.032
	F1 Score	0.869±0.028	0.887±0.032 ●	0.839±0.030
Multi-omics (b+c)	Accuracy	0.852±0.021 ●	0.921±0.022	0.853±0.025
	AUROC	**0.957±0.019**	0.978±0.014	0.965±0.012
	Precision	0.862±0.020 ●	**0.958±0.054**	0.858±0.025
	F1 Score	0.852±0.017 ●	0.914±0.026	0.853±0.025
Multi-omics (a+b+c)	Accuracy	**0.895±0.031**	**0.935±0.024**	**0.859±0.023**
	AUROC	0.955±0.016	0.982±0.009	0.961±0.014
	Precision	**0.898±0.033**	0.931±0.041	**0.862±0.024**
	F1 Score	**0.894±0.032**	**0.933±0.025**	**0.856±0.024**
Multi-omics fusion (independent learning)	Accuracy	0.844±0.034	0.899±0.023	0.829±0.028
	AUROC	0.954±0.011	0.955±0.015	0.939±0.021
	Precision	0.851±0.036	0.896±0.046	0.836±0.029
	F1 Score	0.845±0.034	0.896±0.021	0.825±0.027
Multi-omics fusion (joint learning)	Accuracy	0.877±0.032	0.916±0.037	0.852±0.023
	AUROC	0.957±0.018	0.971±0.014	0.949±0.022
	Precision	0.881±0.034	0.914±0.041	0.853±0.026
	F1 Score	0.875±0.033	0.912±0.042	0.847±0.025

Note: Independent learning (single-omics independent learning) refers to the training strategy of optimizing CancerSD using only a single type of omics data. The multi-omics fusion under independent learning involves initially constructing and training a separate CancerSD model for each omics data to diagnose subtypes. Next, it obtains the omics embedding encoded by omics-specific feature extractors during the diagnosis process. Following that, it constructs a fusion network to integrate these embeddings and obtain the multi-omics fusion representation. Finally, it utilizes this representation for subtype diagnosis. In contrast, joint learning (multi-omics joint learning) involves simultaneously using multiple types of omics data during the optimization process. Moreover, "Multi-omics fusion" implies fixing the embeddings of various omics and reconstructing and optimizing a fusion network for them. '●' indicates CancerSD performs significantly better than the other method.

Table M. Cancer subtype diagnosis performance of well-performing methods (RFC, MOMA, APADC, and CancerSD) on STAD under different omics types.

Omics Type	Metric	RFC	MOMA	APADC	CancerSD
DNA methylation (a)	Accuracy	0.786±0.023	0.810±0.037	**0.819±0.031**	0.802±0.028
	AUROC	0.915±0.026 •	0.934±0.021	0.926±0.022	**0.943±0.012**
	Precision	0.785±0.039 •	0.809±0.040	**0.822±0.028**	0.820±0.029
	F1 Score	0.764±0.025 •	0.804±0.039	**0.809±0.027**	0.805±0.027
miRNA expression (b)	Accuracy	0.659±0.032 •	0.738±0.033	**0.779±0.037**	0.772±0.041
	AUROC	0.889±0.026 •	0.899±0.022 •	0.924±0.017	**0.929±0.024**
	Precision	0.687±0.058 •	0.756±0.038 •	0.787±0.046	**0.805±0.031**
	F1 Score	0.608±0.044 •	0.739±0.034 •	**0.777±0.041**	0.776±0.040
mRNA expression (c)	Accuracy	0.779±0.026 •	0.811±0.033	0.819±0.030	**0.821±0.036**
	AUROC	0.939±0.011	0.938±0.018	0.938±0.016	**0.941±0.018**
	Precision	0.796±0.029 •	0.833±0.027	0.836±0.028	**0.847±0.028**
	F1 Score	0.766±0.029 •	0.813±0.031	0.820±0.029	**0.827±0.033**
Multi-omics (a+b)	Accuracy	0.790±0.034 •	0.815±0.017 •	0.828±0.025 •	**0.857±0.028**
	AUROC	0.925±0.029 •	0.943±0.017	0.917±0.020 •	**0.950±0.012**
	Precision	0.801±0.032 •	0.839±0.014 •	0.835±0.025 •	**0.864±0.028**
	F1 Score	0.771±0.036 •	0.814±0.016 •	0.820±0.026 •	**0.867±0.031**
Multi-omics (a+c)	Accuracy	0.797±0.031 •	0.844±0.025 •	0.839±0.022 •	**0.869±0.027**
	AUROC	0.927±0.032 •	0.951±0.017	0.914±0.019 •	**0.956±0.016**
	Precision	0.810±0.041 •	0.849±0.030 •	0.847±0.028 •	**0.879±0.030**
	F1 Score	0.781±0.033 •	0.840±0.025 •	0.834±0.023 •	**0.869±0.028**
Multi-omics (b+c)	Accuracy	0.779±0.030 •	0.817±0.027 •	0.820±0.025 •	**0.852±0.021**
	AUROC	0.939±0.012 •	0.932±0.036	0.927±0.025 •	**0.957±0.019**
	Precision	0.795±0.031 •	0.833±0.030 •	0.820±0.028 •	**0.862±0.020**
	F1 Score	0.766±0.032 •	0.819±0.026 •	0.809±0.029 •	**0.852±0.017**
Multi-omics (a+b+c)	Accuracy	0.803±0.032 •	0.834±0.025 •	0.854±0.026 •	**0.895±0.031**
	AUROC	0.928±0.029 •	0.955±0.014	**0.956±0.021**	0.955±0.016
	Precision	0.816±0.044 •	0.853±0.034 •	0.863±0.028 •	**0.898±0.033**
	F1 Score	0.789±0.031 •	0.838±0.026 •	0.849±0.029 •	**0.894±0.032**

Note: The best results are shown in **bold** face. '•' indicates CancerSD performs significantly better than the other method. The statistical significance is assessed at the 95% level by student t-test.

Table N. Summary of different cancer datasets after masking.

Dataset	Cancer	Number of Samples (Proportion)						
		total	masked	w/o meth	w/o miRNA	w/o mRNA	w/o one omics	w/o two omics
STAD	gastric	301	226 (75.1%)	109 (36.2%)	119 (39.5%)	112 (37.2%)	112 (37.2%)	112 (37.2%)
ADSC	lung	984	738 (75.0%)	367 (37.3%)	372 (37.8%)	383 (38.9%)	354 (36.0%)	384 (39.0%)
BRCA	breast	881	661 (75.0%)	330 (37.5%)	330 (37.5%)	348 (39.5%)	314 (35.6%)	347 (39.4%)

Note: masked means that the multi-omics data for the sample are simulated as incomplete data. meth refers to DNA methylation data, miRNA refers to miRNA expression data and mRNA refers to mRNA expression data. "w/o" stands for "without," indicating the absence of the corresponding omics data.

Table O. Top-100 ranked important CpG sites identified by CancerSD.

Rank	CpG Site	Importance Score	Rank	CpG Site	Importance Score	Rank	CpG Site	Importance Score
1	cg11620873	1.6889e-02	35	cg19778698	1.5304e-02	68	cg17870792	1.4942e-02
2	cg24946544	1.6738e-02	36	cg14998713	1.5274e-02	69	cg26991199	1.4936e-02
3	cg20951821	1.6695e-02	37	cg07215749	1.5273e-02	70	cg18592307	1.4923e-02
4	cg18084798	1.6565e-02	38	cg04922833	1.5273e-02	71	cg21890738	1.4910e-02
5	cg03608167	1.6513e-02	39	cg15878317	1.5264e-02	72	cg11638200	1.4906e-02
6	cg26904406	1.6478e-02	40	cg26764555	1.5258e-02	73	cg26703664	1.4896e-02
7	cg20652640	1.6326e-02	41	cg21421701	1.5254e-02	74	cg26071978	1.4894e-02
8	cg01655355	1.6326e-02	42	cg20750215	1.5241e-02	75	cg05927017	1.4882e-02
9	cg16427670	1.6320e-02	43	cg04473302	1.5234e-02	76	cg14350002	1.4881e-02
10	cg14402472	1.6266e-02	44	cg12706983	1.5209e-02	77	cg22464423	1.4877e-02
11	cg10553028	1.6200e-02	45	cg15628498	1.5203e-02	78	cg06335889	1.4873e-02
12	cg13843773	1.6111e-02	46	cg08954856	1.5196e-02	79	cg07710481	1.4866e-02
13	cg27543230	1.6018e-02	47	cg05112986	1.5170e-02	80	cg22771603	1.4857e-02
14	cg16861508	1.5736e-02	48	cg03332113	1.5139e-02	81	cg12104707	1.4854e-02
15	cg19840387	1.5736e-02	49	cg20028291	1.5090e-02	82	cg02613803	1.4846e-02
16	cg25169784	1.5671e-02	50	cg17731079	1.5077e-02	83	cg17965230	1.4821e-02
17	cg12548454	1.5635e-02	51	cg06626655	1.5069e-02	84	cg16174234	1.4820e-02
18	cg11784785	1.5612e-02	52	cg09462826	1.5067e-02	85	cg12012426	1.4818e-02
19	cg27309253	1.5609e-02	53	cg25999015	1.5059e-02	86	cg20947775	1.4808e-02
20	cg16539981	1.5603e-02	54	cg24793470	1.5050e-02	87	cg11692477	1.4805e-02
21	cg07212894	1.5601e-02	55	cg27194921	1.5043e-02	88	cg13325529	1.4790e-02
22	cg24170535	1.5575e-02	56	cg04733302	1.5041e-02	89	cg21518938	1.4783e-02
23	cg06073471	1.5538e-02	57	cg14679587	1.5028e-02	90	cg21549904	1.4780e-02
24	cg09837361	1.5498e-02	58	cg16338347	1.5025e-02	91	cg17414431	1.4764e-02
25	cg12177743	1.5485e-02	59	cg22050705	1.5023e-02	92	cg04195127	1.4760e-02
26	cg27376271	1.5482e-02	60	cg27011042	1.4990e-02	93	cg19237753	1.4756e-02
27	cg24777065	1.5474e-02	61	cg25263140	1.4990e-02	94	cg08682341	1.4743e-02
28	cg18676237	1.5372e-02	62	cg07273415	1.4983e-02	95	cg11278262	1.4734e-02
29	cg10525488	1.5372e-02	63	cg20970875	1.4978e-02	96	cg20583316	1.4734e-02
30	cg15973234	1.5356e-02	64	cg22476295	1.4966e-02	97	cg11574612	1.4725e-02
31	cg19088651	1.5354e-02	65	cg11840540	1.4966e-02	98	cg26660414	1.4720e-02
32	cg05449414	1.5320e-02	66	cg04333463	1.4957e-02	99	cg19275050	1.4716e-02
33	cg15702701	1.5320e-02	67	cg16640096	1.4954e-02	100	cg26842024	1.4715e-02
34	cg08026144	1.5310e-02						

Table P. Researches related to the top-ranked important molecules identified by CancerSD in gastric cancer (Continued on the next page).

Source	Molecule	Reference	Description
DNA methylation profiles	*CD9*	Nakamoto et al. [18]	Anti-CD9 antibody ALB6 suppressed tumor growth and proliferation, induced apoptosis, and inhibited angiogenesis in human gastric cancer xenografts. These results suggest that ***CD9*** might be a new attractive targeting molecule in gastric cancer therapy.
	CXCR4	Yasumoto et al. [19]<br>Hashimoto et al. [20]<br>Xiang et al. [21]	***CXCR4*/*CXC12* axis** plays an important role in the development of peritoneal carcinomatosis from gastric carcinoma. Thus, ***CXCR4*** may be a potential therapeutic target for peritoneal carcinomatosis of gastric carcinoma; Blocking on the ***CXCR4*/mTOR signalling pathway** may be useful for the future development of a more effective therapeutic strategy for gastric cancer involved in peritoneal dissemination; ***CXCR4*** and *CXCR2* cross-activate each other to promote the metastasis of gastric cancer.
mRNA expression profiles	*KLK6*	Nagahara et al. [22]	The ***KLK6*** gene is markedly overexpressed in gastric cancer tissue and its expression status may be a powerful prognostic indicator for patients with gastric cancer. ***KLK6*** may possibly be a novel target for gastric cancer therapy by gene-silencing procedures.
	HLA-B	Lu et al. [23]	Unveiling how ***HLA-B* evolutionary divergence** influences the ICB response in patients with gastrointestinal cancers, supporting its potential utility as a combinatorial biomarker together with TMB for patient stratification in the future.
	MUC1	Saeki et al. [24]	***MUC1*** is silenced in intestinal metaplasia, a preneoplastic lesion, but frequently reactivated in gastric cancer, and its expression is correlated to poor prognosis.
	MET	Kawakami et al. [25]	Preclinical evidence has suggested that the **HGF–*MET* axis** and ***MET* amplification** are potential "druggable" targets in gastric cancer, with both HGF- or MET-targeted antibodies and *MET* TKIs currently being the subject of intensive clinical investigation.
miRNA expression profiles	hsa-mir-1305	Shi et al. [26]	**miR-1305** inhibits migration, invasion and proliferation in gastric cancer cells via JAK1 and STAT1. circ_0088300 served as a sponge that directly targeted **miR-1305** and promoted gastric cancer cell proliferation, migration and invasion.
	hsa-mir-488	Luo et al. [27]<br>Yang et al. [28]	Overexpression of circPOFUT1 enhanced cell proliferation, migration, invasion and autophagy-associated chemoresistance in gastric cancer, which were suppressed by **miR-488-3p** overexpression; **miR-488** overexpression enhances EZH2-mediated p53 expression via inhibition of *HULC*, potentially delaying the malignant progression of gastric cancer.
	hsa-mir-942	Lu et al. [29]	circ-CEP85L binds to **miR-942-5p** competitively to promote expression of *NFKBIA*, which inhibits the progression of gastric cancer.
	hsa-mir-219a-2	Yang et al. [30]	LBX2-AS1/**miR-219a-2-3p**/FUS/LBX2 positive feedback loop mainly affected the proliferation and apoptosis abilities of gastric cancer cells, offering novel therapeutic targets for the treatment of patients with gastric cancer.

* Remaining part of the table is presented on the next page.

Table P. Researches related to the top-ranked important molecules identified by CancerSD in gastric cancer (Continued).

Source	Molecule	Reference	Description
miRNA expression profiles	hsa-mir-7-2	Zhao et al. [31] Kong et al. [32]	Both gain-of-function and loss-of-function experiments showed that increased **miR-7** expression significantly reduced gastric cancer cell migration and invasion, whereas decreased **miR-7** expression dramatically enhanced cell migration and invasion. In vivo metastasis assays also demonstrated that overexpression of **miR-7** markedly inhibited gastric cancer metastasis; It is possible that the downregulation of **miR-7** contributes to suppression of differentiation, resulting in the promotion of gastric tumorigenesis. **miR-7** may be useful for devising a new preventive or therapeutic strategy against gastric cancer through the induction of cancer cell differentiation.
	hsa-mir-944	Ma et al. [33]	**hsa-miR-944** specifically affected the growth, migration, or invasion of gastric cancer cells through *PPM1E* targeting. These results suggest that **hsa-miR-944** may regulate the progression of gastric cancer in combination with *PPM1E*.

Note: We selectively choose works that are representative and influential in the relevant field.

Table Q. KEGG pathway-based enrichment analysis results for EBV subtype of gastric cancer (Continued on the next page).

Module	KEGG ID	Description	p value	p.adjust	Genes
Module-2	ko04510	**Focal adhesion***	2.57E-05	2.94E-03	*FLNC, ITGA7, MAPK10, ITGA8, CAV1, COL4A3, ITGA9*
	ko04514	**Cell adhesion molecules (CAMs)**	4.56E-05	2.94E-03	*NRXN3, JAM2, ITGA8, CLDN5, ITGA9, SELP*
	ko05412	Arrhythmogenic right ventricular cardiomyopathy (ARVC)	3.85E-04	1.66E-02	*ITGA7, SLC8A2, ITGA8, ITGA9*
	ko04512	**ECM-receptor interaction**	6.41E-04	1.80E-02	*ITGA7, ITGA8, COL4A3, ITGA9*
	ko05410	Hypertrophic cardiomyopathy (HCM)	6.98E-04	1.80E-02	*ITGA7, SLC8A2, ITGA8, ITGA9*
	ko05414	Dilated cardiomyopathy (DCM)	8.90E-04	1.91E-02	*ITGA7, SLC8A2, ITGA8, ITGA9*
Module-3	-	-	-	-	-
Module-4	ko04061	**Viral protein interaction with cytokine and cytokine receptor***	8.70E-09	1.30E-06	*CXCL10, CXCL11, ACKR4, IL2RG, CCL4L2, TNFSF14, CX3CL1, CCL22*
	ko04060	**Cytokine-cytokine receptor interaction**	2.91E-08	2.16E-06	*CD4, CXCL10, CXCL11, ACKR4, BMP8A, IL2RG, CCL4L2, TNFSF14, TNFSF13B, CX3CL1, CCL22*
	ko04064	**NF-kappa B signaling pathway***	4.74E-06	2.35E-04	*PRKCB, CCL4L2, BIRC3, TNFSF14, TNFSF13B, VCAM1*
	ko04062	**Chemokine signaling pathway**	1.51E-05	5.64E-04	*PRKCB, CXCL10, CXCL11, NCF1, CCL4L2, CX3CL1, CCL22*
	ko04658	**Th1 and Th2 cell differentiation**	4.69E-05	1.40E-03	*CD4, HLA-DQA1, HLA-DQA2, CD3D, IL2RG*
	ko04672	**Intestinal immune network for IgA production**	5.90E-05	1.46E-03	*CD28, HLA-DQA1, HLA-DQA2, TNFSF13B*
	ko04659	**Th17 cell differentiation**	9.67E-05	2.06E-03	*CD4, HLA-DQA1, HLA-DQA2, CD3D, IL2RG*
	ko04623	**Cytosolic DNA-sensing pathway**	1.59E-04	2.96E-03	*CXCL10, AIM2, CASP1, CCL4L2*
	ko05140	Leishmaniasis	3.28E-04	5.40E-03	*PRKCB, NCF1, HLA-DQA1, HLA-DQA2*
	ko04612	**Antigen processing and presentation**	3.63E-04	5.40E-03	*CD4, HLA-DQA1, HLA-DQA2, CTSS*
	ko04514	**Cell adhesion molecules (CAMs)**	4.28E-04	5.80E-03	*CD28, CD4, HLA-DQA1, HLA-DQA2, VCAM1*
	ko04145	Phagosome	4.99E-04	5.97E-03	*MPO, NCF1, HLA-DQA1, HLA-DQA2, CTSS*
	ko05143	African trypanosomiasis	5.55E-04	5.97E-03	*PRKCB, IDO1, VCAM1*
	ko05330	Allograft rejection	6.01E-04	5.97E-03	*CD28, HLA-DQA1, HLA-DQA2*
	ko05340	Primary immunodeficiency	6.01E-04	5.97E-03	*CD4, CD3D, IL2RG*
	ko05323	Rheumatoid arthritis	7.08E-04	6.45E-03	*CD28, HLA-DQA1, HLA-DQA2, TNFSF13B*
	ko05332	Graft-versus-host disease	7.53E-04	6.45E-03	*CD28, HLA-DQA1, HLA-DQA2*
	ko05164	Influenza A	8.30E-04	6.45E-03	*PRKCB, CXCL10, HLA-DQA1, CASP1, HLA-DQA2*
	ko04640	Hematopoietic cell lineage	8.62E-04	6.45E-03	*CD4, HLA-DQA1, HLA-DQA2, CD3D*
	ko04940	Type I diabetes mellitus	8.66E-04	6.45E-03	*CD28, HLA-DQA1, HLA-DQA2*

* Remaining part of the table is presented on the next page.

Table Q. KEGG pathway-based enrichment analysis results for EBV subtype of gastric cancer (Continued).

Module	KEGG ID	Description	p value	p.adjust	Genes
Module-4	ko04668	**TNF signaling pathway**	1.42E-03	1.01E-02	*CXCL10, BIRC3, CX3CL1, VCAM1*
	ko05320	Autoimmune thyroid disease	1.60E-03	1.08E-02	*CD28, HLA-DQA1, HLA-DQA2*
	ko05416	Viral myocarditis	2.28E-03	1.48E-02	*CD28, HLA-DQA1, HLA-DQA2*
	ko05166	Human T-cell leukemia virus 1 infection	2.56E-03	1.59E-02	*CD4, HLA-DQA1, HLA-DQA2, CD3D, IL2RG*
	ko05321	Inflammatory bowel disease (IBD)	2.87E-03	1.71E-02	*HLA-DQA1, HLA-DQA2, IL2RG*
	ko05235	**PD-L1 expression and PD-1 checkpoint pathway in cancer***	6.94E-03	3.98E-02	*CD28, CD4, CD3D*
	ko05310	Asthma	8.15E-03	4.50E-02	*HLA-DQA1, HLA-DQA2*
	ko05150	Staphylococcus aureus infection	8.55E-03	4.55E-02	*C5AR1, HLA-DQA1, HLA-DQA2*
Module-5	ko4974	**Protein digestion and absorption**	2.40E-05	1.15E-03	*COL11A1, COL1A1, COL21A1, COL4A6*
	ko4512	**ECM-receptor interaction**	5.49E-04	1.01E-02	*FN1, COL1A1, COL4A6*
	ko4933	AGE-RAGE signaling pathway in diabetic complications	7.98E-04	1.01E-02	*FN1, COL1A1, COL4A6*
	ko5146	Amoebiasis	8.45E-04	1.01E-02	*FN1, COL1A1, COL4A6*
Module-6	ko4512	**ECM-receptor interaction**	4.43E-04	9.54E-03	*COL6A3, THBS2, ITGA11*
	ko0514	**Other types of O-glycan biosynthesis**	6.58E-04	9.54E-03	*ST6GAL2, GXYLT2*
	ko5144	Malaria	3.26E-03	3.15E-02	*GYPC, THBS2*
	ko4510	**Focal adhesion***	4.65E-03	3.37E-02	*COL6A3, THBS2, ITGA11*

Note: The term 'p.adjust' signifies the p-value after undergoing False Discovery Rate (FDR) correction. In this table, pathways indicated in **bold** have been confirmed to be associated with gastric cancer (or other digestive cancers), while terms in **bold** with an '*' signify pathways potentially or already confirmed to be associated with the EBV subtype of gastric cancer. '-' denotes that there it no enriched pathway in the corresponding module.

Table R. Univariate and multivariate analyses of clinical variables associated with overall survival in patients with gastric cancer.

Variable	Value	Proportion	Univariate			Multivariate		
			HR	95%CI	p value	HR	95%CI	p value
Diagnosis Age	< 64	40.1%	Reference			Reference		
	65-75	37.3%	1.74	1.15-2.63	0.01	2.07	1.32-3.24	< 0.005
	≥ 75	22.6%	1.59	0.98-2.59	0.06	2.17	1.27-3.71	< 0.005
Gender	Male	63.8%	Reference					
	Female	36.2%	0.78	0.53-1.15	0.20	0.78	0.52-1.16	0.22
Lauren Classification	diffuse	18.1%	Reference			Reference		
	intestinal	68.0%	0.84	0.53-1.32	0.45	0.73	0.45-1.18	0.19
	mixed	9.2%	1.31	0.56-3.05	0.53	0.79	0.31-2.02	0.63
	other	4.7%	0.91	0.42-1.97	0.82	0.69	0.30-1.61	0.39
Tumor Stage	T1	4.7%	Reference			Reference		
	T2	17.3%	4.74	0.63-35.54	0.13	2.78	0.34-22.52	0.34
	T3	49.9%	6.57	0.91-47.38	0.06	4.44	0.48-40.83	0.19
	T4	25.9%	7.65	1.05-55.95	0.05	4.71	0.51-43.87	0.17
	TX	2.2%	14.91	1.34-165.33	0.03	4.04	0.14-114.34	0.41
Lymph Node Stage	N0	28.8%	Reference			Reference		
	N1	25.1%	1.35	0.79-2.31	0.28	1.15	0.54-2.48	0.72
	N2	21.5%	1.69	0.97-2.93	0.06	1.20	0.48-3.05	0.69
	N3	20.1%	2.37	1.42-3.98	< 0.005	1.51	0.59-3.85	0.38
	NX	4.5%	2.72	0.94-7.87	0.06	2.29	0.59-8.94	0.23
Metatasis Stage	M0	89.7%	Reference			Reference		
	M1	6.7%	2.35	1.29-4.28	0.01	1.28	0.49-3.39	0.61
	MX	3.6%	1.94	0.79-4.76	0.15	2.13	0.70-6.45	0.18
TNM Stage	STAGE I	12.7%	Reference			Reference		
	STAGE II	33.8%	1.38	0.65-2.95	0.40	0.85	0.26-2.75	0.78
	STAGE III	43.4%	2.38	1.18-4.80	0.02	1.05	0.23-4.77	0.94
	STAGE IV	10.1%	4.03	1.85-8.81	< 0.005	1.91	0.44-8.27	0.39
ICS Level	Low	30.6%	Reference			Reference		
	High	69.4%	1.54	1.01-2.34	**0.04**	1.7	1.07-2.70	**0.03**

Note: HR and 95%CI are abbreviations of Hazard Ratio and 95% Confidence Interval, respectively. IGS are categorized into low and high level by a cutoff point of 16.2. In addition, TX, NX, and MX indicate that it is not possible to accurately assess the current stage of patients.

Table S. Researches related to the relationships between important genes and stemness features of cancer cells.

Gene	Analysis or Description
MET	c-Met, the protein product of the *MET* proto-oncogene, has been demonstrated to promote tumor angiogenesis, growth, and metastasis [34]. The combination of c-Met inhibitors with SN38 may effectively target cancer stem cells in diffuse-type gastric cancer [35]. c-Met/ALK inhibitors could reduce the expression of cancer stem cell markers in gastrointestinal cancers [36].
ECRG4	*ECRG4* serves as an inhibitory upstream regulator of the NF-κB pathway [37], while the latter is persistently activated in cancer stem cells across various malignancies, participating in several crucial biological processes of cancer stem cells.
PPP1R12B	*PEAK1-PPP1R12B* axis can inhibit cell growth and metastasis in colorectal cancer by attenuating the Grb2/PI3K/Akt signaling pathway [38]. Moreover, Considering the activation effect of PI3K/Akt pathway on the NF-κB system [39], the high expression of *PPP1R12B* might indirectly play a role in suppressing the stemness of gastric cancer cells.
VSIG1	*VSIG1* is a cell-cell adhesion molecule, which may play a role in modulating epithelial-mesenchymal transition (EMT) during tumor metastasis [40], while EMT can promote tumor cells to generate cancer stem cell [41].
PLEKHG4	In vitro assays revealed that *PLEKHG4* promoted Thyroid Cancer cell proliferation, migration, invasion, and epithelial-mesenchymal transformation. Knockdown of *PLEKHG4* led to the opposite effects, and the loss of *PLEKHG4* enhanced the apoptosis ability and inhibited the stemness properties of Thyroid Cancer cells [42].

Table T. The comparison of knowledge transfer performance across different lung cancer dataset.

Method	Metric	ADSC→CPTAC		CPTAC→ADSC	
		5-shot	10-shot	5-shot	10-shot
kNN	Accuracy	0.736±0.038 •	0.766±0.007 •	0.833±0.012 •	0.846±0.021 •
	AUROC	0.813±0.025 •	0.785±0.012 •	0.936±0.006 •	0.934±0.006 •
	Precision	0.593±0.083 •	0.700±0.020	**0.981±0.005**	**0.981±0.006**
	F1 Score	0.593±0.033 •	0.509±0.016 •	0.799±0.019 •	0.816±0.031 •
RFC	Accuracy	0.705±0.077 •	0.778±0.024 •	0.842±0.028 •	0.898±0.011 •
	AUROC	0.858±0.023 •	0.849±0.032 •	0.911±0.015 •	0.952±0.005 •
	Precision	0.552±0.110 •	0.650±0.080 •	0.917±0.024 •	0.927±0.034 •
	F1 Score	0.628±0.032 •	0.632±0.044 •	0.822±0.038 •	0.892±0.012 •
DeepBDC	Accuracy	0.791±0.013 •	0.806±0.024 •	0.903±0.004 •	0.902±0.003 •
	AUROC	0.843±0.029 •	0.869±0.025 •	0.945±0.002 •	0.946±0.002 •
	Precision	**0.810±0.126**	0.700±0.094	0.937±0.013 •	0.941±0.011 •
	F1 Score	0.572±0.087 •	0.677±0.061 •	0.897±0.004 •	0.896±0.004 •
QSFormer	Accuracy	0.804±0.010 •	0.814±0.008 •	0.903±0.008 •	0.905±0.006 •
	AUROC	0.870±0.010 •	0.861±0.009 •	0.942±0.005 •	0.945±0.003 •
	Precision	0.803±0.112	**0.910±0.070**	0.918±0.023 •	0.917±0.025 •
	F1 Score	0.619±0.045 •	0.587±0.019 •	0.900±0.007 •	0.902±0.004 •
MOMA-PT	Accuracy	0.773±0.028 •	0.783±0.023 •	0.876±0.012 •	0.888±0.018 •
	AUROC	0.860±0.018 •	0.866±0.013 •	0.936±0.009 •	0.951±0.007 •
	Precision	0.625±0.071 •	0.621±0.051 •	0.913±0.025 •	0.880±0.052 •
	F1 Score	0.667±0.021 •	0.678±0.016 •	0.868±0.017 •	0.889±0.015 •
DCP-PT	Accuracy	0.769±0.033 •	0.789±0.022 •	0.879±0.035 •	0.889±0.018 •
	AUROC	0.793±0.048 •	0.823±0.046 •	**0.989±0.021**	0.939±0.014 •
	Precision	0.665±0.088 •	0.684±0.055 •	0.939±0.034	0.933±0.023 •
	F1 Score	0.615±0.054 •	0.652±0.036 •	0.867±0.040 •	0.881±0.021 •
CancerSD-PT	Accuracy	0.776±0.046 •	0.814±0.025 •	0.888±0.014 •	0.900±0.027 •
	AUROC	0.884±0.020 •	0.902±0.022	0.944±0.007 •	0.952±0.006 •
	Precision	0.622±0.075 •	0.668±0.049 •	0.917±0.052 •	0.902±0.058 •
	F1 Score	0.689±0.030	0.719±0.035 •	0.883±0.012 •	0.900±0.021 •
CancerSD-MOMA	Accuracy	0.794±0.041 •	0.821±0.015 •	0.891±0.007 •	0.900±0.019 •
	AUROC	0.889±0.013 •	0.896±0.011 •	0.937±0.004 •	0.948±0.009 •
	Precision	0.645±0.072 •	0.680±0.034 •	0.920±0.028 •	0.902±0.049 •
	F1 Score	0.710±0.026	0.727±0.017 •	0.886±0.010 •	0.899±0.015 •
CancerSD-DCP	Accuracy	0.793±0.028 •	0.814±0.021 •	0.902±0.016 •	0.914±0.017 •
	AUROC	0.830±0.035 •	0.858±0.025 •	0.958±0.010 •	0.963±0.013 •
	Precision	0.702±0.092 •	0.723±0.063	0.923±0.045	0.938±0.017 •
	F1 Score	0.658±0.052 •	0.693±0.046 •	0.898±0.013 •	0.910±0.017 •
CancerSD-SIM	Accuracy	0.784±0.068 •	0.801±0.032 •	0.906±0.018 •	0.910±0.008 •
	AUROC	0.878±0.032 •	0.825±0.052 •	0.951±0.006 •	0.954±0.003 •
	Precision	0.694±0.129	0.794±0.140	0.923±0.042 •	0.932±0.020 •
	F1 Score	0.662±0.076	0.615±0.070 •	0.903±0.015 •	0.906±0.009 •
CancerSD-w/oCLC	Accuracy	0.796±0.037 •	0.832±0.011 •	0.894±0.012 •	0.902±0.015 •
	AUROC	0.908±0.013	0.919±0.007	0.937±0.003 •	0.955±0.006 •
	Precision	0.648±0.086 •	0.692±0.037	0.915±0.040 •	0.910±0.046 •
	F1 Score	**0.719±0.020**	0.746±0.017	0.890±0.009 •	0.900±0.013 •
CancerSD	Accuracy	**0.835±0.020**	**0.848±0.019**	**0.947±0.003**	**0.949±0.006**
	AUROC	**0.910±0.010**	**0.920±0.008**	0.980±0.001	**0.981±0.002**
	Precision	0.788±0.076	0.740±0.062	0.961±0.006	0.961±0.015
	F1 Score	0.710±0.045	**0.759±0.021**	**0.945±0.004**	**0.947±0.006**

Note: ADSC → CPTAC means to transfer the knowledge from ADSC to CPTAC, that is, using ADSC as the meta training set and CPTAC as the meta testing set. The ADSC dataset is obtained by merging the TCGA LUAD and LUSC datasets. The best results are shown in **bold** face. '•' indicates CancerSD performs significantly better than the other method. The statistical significance is assess at the 95% level by t-test.

Table U. The performance comparison of Imputation Algorithms on STAD dataset (measured by Mean Absolute Error).

Missing Omics	Missing Rate	Statistical		Machine learning-based		Deep learning-based			
		Zero	Mean [43]	KNNI [44]	MissFI [45]	GAIN [46]	OTI [47]	TDM [48]	CancerSD
meth	25%	0.035±0.007	0.024±0.005	0.021±0.004	0.022±0.003	0.019±0.002	0.020±0.001	**0.016±0.002**	0.018±0.002
	50%	0.066±0.008	0.046±0.007	0.040±0.006	0.041±0.005	0.036±0.004	**0.032±0.004**	0.033±0.003	0.037±0.004
	75%	0.101±0.007	0.071±0.005	0.063±0.005	0.067±0.006	0.058±0.004	0.054±0.003	**0.052±0.005**	0.056±0.005
	90%	0.119±0.005	0.085±0.004	0.080±0.004	0.077±0.005	0.074±0.007	0.072±0.003	**0.070±0.002**	0.076±0.004
miRNA	25%	0.763±0.137	0.161±0.028	0.146±0.028	0.152±0.030	0.143±0.020	0.138±0.015	**0.135±0.021**	0.144±0.020
	50%	1.479±0.194	0.312±0.044	0.284±0.037	0.279±0.045	0.270±0.030	0.265±0.026	0.265±0.018	**0.258±0.031**
	75%	2.251±0.166	0.484±0.038	0.444±0.035	0.465±0.036	0.437±0.028	0.420±0.025	**0.413±0.011**	0.427±0.025
	90%	2.657±0.099	0.574±0.027	0.549±0.024	0.540±0.025	0.525±0.018	0.524±0.017	0.513±0.019	**0.502±0.020**
mRNA	25%	0.481±0.087	0.173±0.031	0.157±0.029	0.159±0.021	0.146±0.018	0.143±0.015	0.138±0.017	**0.136±0.017**
	50%	0.920±0.126	0.336±0.044	0.307±0.039	0.300±0.032	0.293±0.026	0.286±0.022	**0.263±0.023**	0.274±0.025
	75%	1.419±0.113	0.521±0.040	0.488±0.038	0.476±0.035	0.476±0.031	0.472±0.028	0.461±0.019	**0.440±0.022**
	90%	1.670±0.069	0.627±0.027	0.609±0.021	0.605±0.020	0.584±0.018	0.573±0.013	0.576±0.014	**0.548±0.022**

Note: meth refers to DNA methylation data, miRNA refers to miRNA expression data and mRNA refers to mRNA expression data. Results shown in terms of mean±std of MAE under different missing rates. MAE is the Mean Absolute Error. The best results and the second-best results are highlighted in **bold** face and in underline.

Table V. The performance comparison of Imputation Algorithms on STAD dataset (measured by Root Mean Square Error).

Missing Omics	Missing Rate	Statistical		Machine learning-based		Deep learning-based			
		Zero	Mean	KNNI	MissFI	GAIN	OTI	TDM	CancerSD
meth	25%	0.243±0.015	0.165±0.007	0.156±0.006	0.159±0.005	0.153±0.004	0.150±0.003	0.152±0.004	**0.145±0.005**
	50%	0.244±0.009	0.166±0.006	0.160±0.006	0.158±0.004	0.156±0.003	0.157±0.004	0.153±0.003	**0.150±0.004**
	75%	0.243±0.006	0.167±0.003	0.163±0.005	0.162±0.004	0.161±0.002	0.160±0.002	0.158±0.003	**0.157±0.004**
	90%	0.244±0.005	0.168±0.003	0.169±0.003	0.168±0.002	0.167±0.002	0.168±0.003	**0.164±0.005**	0.167±0.006
miRNA	25%	4.830±0.063	0.930±0.059	0.851±0.053	0.869±0.055	0.837±0.046	0.827±0.042	**0.813±0.040**	0.824±0.037
	50%	4.863±0.061	0.961±0.051	0.879±0.030	0.870±0.040	0.863±0.031	0.854±0.030	0.852±0.027	**0.841±0.030**
	75%	4.832±0.039	0.966±0.038	0.896±0.026	0.885±0.036	0.876±0.033	0.861±0.023	0.851±0.034	**0.837±0.017**
	90%	4.830±0.041	0.973±0.038	0.939±0.048	0.935±0.022	0.921±0.028	0.913±0.025	**0.910±0.019**	0.929±0.020
mRNA	25%	2.745±0.034	1.010±0.032	0.955±0.069	0.940±0.023	0.906±0.029	0.891±0.022	0.876±0.024	**0.856±0.026**
	50%	2.744±0.020	1.026±0.031	0.973±0.044	0.964±0.030	0.907±0.026	0.901±0.021	0.885±0.016	**0.866±0.016**
	75%	2.745±0.017	1.037±0.015	0.993±0.026	0.985±0.037	0.953±0.022	0.946±0.018	0.947±0.029	**0.926±0.026**
	90%	2.738±0.020	1.044±0.015	1.036±0.023	1.033±0.016	1.011±0.016	0.993±0.015	0.981±0.017	**0.976±0.026**

Note: meth refers to DNA methylation data, miRNA refers to miRNA expression data and mRNA refers to mRNA expression data. Results shown in terms of mean±std of RMSE under different missing rates. RMSE is the Root Mean Square Error. The best results and the second-best results are highlighted in **bold** face and in underline.

References

[1] Weinstein JN, Collisson EA, Mills GB, Shaw KR, Ozenberger BA, Ellrott K, et al. The cancer genome atlas pan-cancer analysis project. Nature Genetics. 2013;45(10):1113–1120. https://doi.org/10.1038/ng.2764 PMID:24071849

[2] Edgar R, Domrachev M, Lash AE. Gene Expression Omnibus: NCBI gene expression and hybridization array data repository. Nucleic Acids Research. 2002;30(1):207–210. https://doi.org/10.1093/nar/30.1.207 PMID:11752295

[3] Whiteaker JR, Halusa GN, Hoofnagle AN, Sharma V, MacLean B, Yan P, et al. CPTAC Assay Portal: a repository of targeted proteomic assays. Nature Methods. 2014;11(7):703–704. https://doi.org/10.1038/nmeth.3002 PMID:24972168

[4] Edwards NJ, Oberti M, Thangudu RR, Cai S, McGarvey PB, Jacob S, et al. The CPTAC data portal: a resource for cancer proteomics research. Journal of Proteome Research. 2015;14(6):2707–2713. https://doi.org/10.1021/pr501254j PMID:25873244

[5] Breiman L. Random forests. Machine Learning. 2001;45:5–32. https://doi.org/10.1023/A:1010933404324

[6] Ma B, Meng F, Yan G, Yan H, Chai B, Song F. Diagnostic classification of cancers using extreme gradient boosting algorithm and multi-omics data. Computers in Biology and Medicine. 2020;121:103761. https://doi.org/10.1016/j.compbiomed.2020.103761 PMID:32339094

[7] Moon S, Lee H. MOMA: a multi-task attention learning algorithm for multi-omics data interpretation and classification. Bioinformatics. 2022;38(8):2287–2296. https://doi.org/10.1093/bioinformatics/btac080 PMID:35157023

[8] Wang T, Shao W, Huang Z, Tang H, Zhang J, Ding Z, et al. MOGONET integrates multi-omics data using graph convolutional networks allowing patient classification and biomarker identification. Nature Communications. 2021;12(1):3445. https://doi.org/10.1038/s41467-021-23774-w PMID:34103512

[9] Argelaguet R, Arnol D, Bredikhin D, Deloro Y, Velten B, Marioni JC, et al. MOFA+: a statistical framework for comprehensive integration of multi-modal single-cell data. Genome Biology. 2020;21:1–17. https://doi.org/10.1186/s13059-020-02015-1 PMID:32393329

[10] Liang PP, Deng Z, Ma MQ, Zou JY, Morency LP, Salakhutdinov R. Factorized contrastive learning: Going beyond multi-view redundancy. Advances in Neural Information Processing Systems. 2024;36.

[11] Bardes A, Ponce J, Lecun Y. VICReg: Variance-Invariance-Covariance Regularization For Self-Supervised Learning. In: ICLR 2022-International Conference on Learning Representations; 2022.

[12] Yang H, Chen R, Li D, Wang Z. Subtype-GAN: a deep learning approach for integrative cancer subtyping of multi-omics data. Bioinformatics. 2021;37(16):2231–2237. https://doi.org/10.1093/bioinformatics/btab109 PMID:33599254

[13] Du JH, Cai Z, Roeder K. Robust probabilistic modeling for single-cell multimodal mosaic integration and imputation via scVAEIT. Proceedings of the National Academy of Sciences. 2022;119(49):e2214414119. https://doi.org/10.1073/pnas.2214414119 PMID:36459654

[14] Lin Y, Gou Y, Liu X, Bai J, Lv J, Peng X. Dual contrastive prediction for incomplete multi-view representation learning. IEEE Transactions on Pattern Analysis and Machine Intelligence. 2022;45(4):4447–4461. https://doi.org/10.1109/tpami.2022.3197238 PMID:35939466

[15] Xu J, Li C, Peng L, Ren Y, Shi X, Shen HT, et al. Adaptive feature projection with distribution alignment for deep incomplete multi-view clustering. IEEE Transactions on Image Processing. 2023;32:1354–1366. https://doi.org/10.1109/tip.2023.3243521 PMID:37022865

[16] Xie J, Long F, Lv J, Wang Q, Li P. Joint distribution matters: Deep brownian distance covariance for few-shot classification. In: Proceedings of the IEEE/CVF Conference on Computer Vision and Pattern Recognition; 2022. p. 7972–7981.

[17] Wang X, Wang X, Jiang B, Luo B. Few-Shot Learning Meets Transformer: Unified Query-Support Transformers for Few-Shot Classification. IEEE Transactions on Circuits and Systems for Video Technology. 2023;33:7789–7802. https://doi.org/10.1109/TCSVT.2023.3282777

[18] Nakamoto T, Murayama Y, Oritani K, Boucheix C, Rubinstein E, Nishida M, et al. A novel therapeutic strategy with anti-CD9 antibody in gastric cancers. Journal of Gastroenterology. 2009;44:889–896. https://doi.org/10.1007/s00535-009-0081-3 PMID:19468669

[19] Yasumoto K, Koizumi K, Kawashima A, Saitoh Y, Arita Y, Shinohara K, et al. Role of the CXCL12/CXCR4 axis in peritoneal carcinomatosis of gastric cancer. Cancer Research. 2006;66(4):2181–2187. https://doi.org/10.1016/j.semcancer.2019.12.007 PMID:31874281

[20] Hashimoto I, Koizumi K, Tatematsu M, Minami T, Cho S, Takeno N, et al. Blocking on the CXCR4/mTOR signalling pathway induces the anti-metastatic properties and autophagic cell death in peritoneal disseminated gastric cancer cells. European Journal of Cancer. 2008;44(7):1022–1029. https://doi.org/10.1016/j.ejca.2008.02.043 PMID:18375114

[21] Xiang Z, Zhou Z, Xia G, Zhang X, Wei Z, Zhu J, et al. A positive crosstalk between CXCR4 and CXCR2 promotes gastric cancer metastasis. Oncogene. 2017;36(36):5122–5133. https://doi.org/10.1038/onc.2017.108 PMID:28481874

[22] Nagahara H, Mimori K, Utsunomiya T, Barnard GF, Ohira M, Hirakawa K, et al. Clinicopathologic and biological significance of kallikrein 6 overexpression in human gastric cancer. Clinical Cancer Research. 2005;11(19):6800–6806. https://doi.org/10.1158/1078-0432.ccr-05-0943 PMID:16203767

[23] Lu Z, Chen H, Jiao X, Wang Y, Wu L, Sun H, et al. Germline HLA-B evolutionary divergence influences the efficacy of immune checkpoint blockade therapy in gastrointestinal cancer. Genome Medicine. 2021;13:1–15. https://doi.org/10.1186/s13073-021-00997-6 PMID:34732240

[24] Saeki N, Sakamoto H, Yoshida T. Mucin 1 gene (MUC1) and gastric-cancer susceptibility. International Journal of Molecular Sciences. 2014;15(5):7958–7973. https://doi.org/10.3390/ijms15057958 PMID:24810688

[25] Kawakami H, Okamoto I. MET-targeted therapy for gastric cancer: the importance of a biomarker-based strategy. Gastric Cancer. 2016;19:687–695. https://doi.org/10.1007/s10120-015-0585-x PMID:26690587

[26] Shi H, Huang S, Qin M, Xue X, Guo X, Jiang L, et al. Exosomal circ_0088300 derived from cancer-associated fibroblasts acts as a miR-1305 sponge and promotes gastric carcinoma cell tumorigenesis. Frontiers in Cell and Developmental Biology. 2021;9:676319. https://doi.org/10.3389/fcell.2021.676319 PMID:34124064

[27] Luo M, Deng X, Chen Z, Hu Y. Circular RNA circPOFUT1 enhances malignant phenotypes and autophagy-associated chemoresistance via sequestrating miR-488-3p to activate the PLAG1-ATG12 axis in gastric cancer. Cell Death & Disease. 2023;14(1):10. https://doi.org/10.1038/s41419-022-05506-0 PMID:36624091

[28] Yang D, Shi M, You Q, Zhang Y, Hu Z, Xu J, et al. Tumor-and metastasis-promoting roles of miR-488 inhibition via HULC enhancement and EZH2-mediated p53 repression in gastric cancer. Cell Biology and Toxicology. 2023;39(4):1341–1358. https://doi.org/10.1007/s10565-022-09760-y PMID:36449143

[29] Lu J, Wang Yh, Huang Xy, Xie Jw, Wang Jb, Lin Jx, et al. circ-CEP85L suppresses the proliferation and invasion of gastric cancer by regulating NFKBIA expression via miR-942-5p. Journal of Cellular Physiology. 2020;235(9):6287–6299. https://doi.org/10.1002/jcp.29556 PMID:32026471

[30] Yang Z, Dong X, Pu M, Yang H, Chang W, Ji F, et al. LBX2-AS1/miR-219a-2-3p/FUS/LBX2 positive feedback loop contributes to the proliferation of gastric cancer. Gastric Cancer. 2020;23:449–463. https://doi.org/10.1007/s10120-019-01019-6 PMID:31673844

[31] Zhao X, Dou W, He L, Liang S, Tie J, Liu C, et al. MicroRNA-7 functions as an anti-metastatic microRNA in gastric cancer by targeting insulin-like growth factor-1 receptor. Oncogene. 2013;32(11):1363–1372. https://doi.org/10.1038/onc.2012.156 PMID:22614005

[32] Kong D, Piao YS, Yamashita S, Oshima H, Oguma K, Fushida S, et al. Inflammation-induced repression of tumor suppressor miR-7 in gastric tumor cells. Oncogene. 2012;31(35):3949–3960. https://doi.org/10.1038/onc.2011.558 PMID:22139078

[33] Ma S, Xinliang G, Shen L, Yinhao C, Chen Q, Xianjuan S, et al. CircHAS2 promotes the proliferation, migration, and invasion of gastric cancer cells by regulating PPM1E mediated by hsa-miR-944. Cell Death and Disease. 2021;12(10). https://doi.org/10.1038/s41419-021-04158-w PMID:34556632

[34] Gu Y, Chen Y, Wei L, Wu S, Shen K, Liu C, et al. ABHD5 inhibits YAP-induced c-Met overexpression and colon cancer cell stemness via suppressing YAP methylation. Nature Communications. 2021;12(1):6711. https://doi.org/10.1038/s41467-021-26967-5 PMID:34795238

[35] Yashiro M, Nishii T, Hasegawa T, Matsuzaki T, Morisaki T, Fukuoka T, et al. A c-Met inhibitor increases the chemosensitivity of cancer stem cells to the irinotecan in gastric carcinoma. British Journal of Cancer. 2013;109(10):2619–2628. https://doi.org/10.1038/bjc.2013.638 PMID:24129235

[36] Bahrami A, Shahidsales S, Khazaei M, Ghayour-Mobarhan M, Maftouh M, Hassanian SM, et al. C-Met as a potential target for the treatment of gastrointestinal cancer: Current status and future perspectives. Journal of Cellular Physiology. 2017;232(10):2657–2673. https://doi.org/10.1002/jcp.25794 PMID:28075018

[37] Li D, Liu X, Liu T, Liu H, Tong L, Jia S, et al. Neurochemical regulation of the expression and function of glial fibrillary acidic protein in astrocytes. Glia. 2020;68(5):878–897. https://doi.org/10.1002/glia.23734 PMID:31626364

[38] Ding C, Tang W, Wu H, Fan X, Luo J, Feng J, et al. The PEAK1–PPP1R12B axis inhibits tumor growth and metastasis by regulating Grb2/PI3K/Akt signalling in colorectal cancer. Cancer Letters. 2019;442:383–395. https://doi.org/10.1016/j.canlet.2018.11.014 PMID:30472186

[39] Chao X, Zao J, Xiao-Yi G, Li-Jun M, Tao S. Blocking of PI3K/AKT induces apoptosis by its effect on NF-κB activity in gastric carcinoma cell line SGC7901. Biomedicine & Pharmacotherapy. 2010;64(9):600–604. https://doi.org/10.1016/j.biopha.2010.08.008 PMID:20947290

[40] Zhou X, Khan S, Huang D, Li L. V-Set and immunoglobulin domain containing (VSIG) proteins as emerging immune checkpoint targets for cancer immunotherapy. Frontiers in Immunology. 2022;13:938470. https://doi.org/10.3389/fimmu.2022.938470 PMID:36189222

[41] Mani SA, Guo W, Liao MJ, Eaton EN, Ayyanan A, Zhou AY, et al. The epithelial-mesenchymal transition generates cells with properties of stem cells. Cell. 2008;133(4):704–715. https://doi.org/10.1016/j.cell.2008.03.027 PMID:18485877

[42] Yuan Q, Fan Y, Liu Z, Wang X, Jia M, Dong Y, et al. Pleckstrin homology and RhoGEF domain containing G4 (PLEKHG4) leads to the activation of RhoGTPases promoting the malignant phenotypes of thyroid cancer. Apoptosis. 2023;28(9):1315–1331. https://doi.org/10.1007/s10495-023-01861-1 PMID:37336836

[43] Farhangfar A, Kurgan LA, Pedrycz W. A novel framework for imputation of missing values in databases. IEEE Transactions on Systems, Man, and Cybernetics-Part A: Systems and Humans. 2007;37(5):692–709. https://doi.org/10.1109/TSMCA.2007.902631

[44] Troyanskaya O, Cantor M, Sherlock G, Brown P, Hastie T, Tibshirani R, et al. Missing value estimation methods for DNA microarrays. Bioinformatics. 2001;17(6):520–525. https://doi.org/10.1093/bioinformatics/17.6.520 PMID:11395428

[45] Stekhoven DJ, Bühlmann P. MissForest—non-parametric missing value imputation for mixed-type data. Bioinformatics. 2012;28(1):112–118. https://doi.org/10.1093/bioinformatics/btr597 PMID:22039212

[46] Yoon J, Jordon J, Schaar M. Gain: Missing data imputation using generative adversarial nets. In: International Conference on Machine Learning. PMLR; 2018. p. 5689–5698.

[47] Muzellec B, Josse J, Boyer C, Cuturi M. Missing data imputation using optimal transport. In: International Conference on Machine Learning. PMLR; 2020. p. 7130–7140.

[48] Zhao H, Sun K, Dezfouli A, Bonilla EV. Transformed distribution matching for missing value imputation. In: International Conference on Machine Learning. PMLR; 2023. p. 42159–42186.
