# Supplementary material for: Cancer molecular subtyping using limited multi-omics data with missingness
Source: PLoS Comput Biol. 2024 Dec 26;20(12):e1012710. doi: 10.1371/journal.pcbi.1012710 (PMC11709273; doi:10.1371/journal.pcbi.1012710)
Supplement: S2 Text — Sections A-J. (PDF) [file pcbi.1012710.s019.pdf]

Supplementary Texts

Section A. The details of the comparison methods

To comprehensively evaluate the performance of the base learner of CancerSD, we compared it with thirteen representative methods.
(i) traditional machine learning, including:

- k-Nearest Neighbor classifier (kNN) is a typical classifier in supervised learning, which determines the sample as the class with the largest proportion among the k samples closest to it.
- Random Forest Classifier (RFC) [1] is an algorithm based on ensemble learning, which accomplishes classification tasks by constructing multiple decision trees and synthesizing their predictive outcomes.

(ii) Multi-omics Integration (MI)-based methods, including:

- AE-XGBoost [2] first constructs an auto-encoder for each omics, then concatenates the low-dimensional embeddings from the bottleneck layer to get a multi-omics fusion representation, and finally employs XGBoost to classify samples.
- MOGONET [3] first utilizes Graph Convolutional Network (GCN) for omics-specific learning, projecting patient features into the label space. Then, it designs a View Correlation Discovery Network (VCDN) to explore cross-omics correlations and diagnose the cancer subtype of patients.
- MOMA [4] employs a geometric method to vectorize genes and modules, identifies related modules in multiple omics views by attention mechanism, and captures important modules in each omics by cross-omics correlation. Finally, it uses a multi-task learning framework to diagnose the subtype of patients.
- MOFA+ [5] provides an unsupervised framework for the integration of multi-view data. It leverages the dependencies between features to learn a low-dimensional representation of the data, defined by K latent factors, which capture the global sources of molecular variability. Subsequently, the learned low-dimensional representations are employed in a series of downstream tasks.
- FactorCL [6] captures task-relevant information by maximizing mutual information (MI) lower bounds while removing irrelevant redundant information by minimizing MI upper bounds. Additionally, FactorCL uses multimodal data augmentations to approximate task relevance without the need for labels. It factorizes task-relevant information into shared and unique representations, which are then fed into a fully connected neural network (FCNN) to diagnose cancer subtypes.
- VICReg [7] explicitly avoids the collapse problem (i.e., when negative samples are absent, the encoder can solely learn a constant representation) by applying a simple regularization term to each dimension of the embeddings independently. It combines the variance term with a decorrelation mechanism based on redundancy reduction and covariance regularization to further optimize the encoder.

(iii) incomplete Multi-omics Integration (iMI)-based methods, including:

- DCP [8] solely recovers the shared information of the missing views and available views, reducing the cost of imputation while ensuring that the learned representation retains the least information that can adequately meet the needs of downstream tasks. In the end, DCP measures the average similarity between the pending patient and patients of each subtype within the training set in the representation space and treat the subtype with the highest similarity as the diagnosis.
- APADC [9] utilizes adaptive feature projection to avoid the imputation of missing data, and considers the distribution alignment between samples with complete and incomplete data to deal with view incompleteness. Finally, it feeds the multi-omics fusion representations into a FCNN to obtain the cancer subtype diagnosis of patients.

- scVAEIT [10] constructs an integrative probabilistic model by utilizing shared and unshared features from different modalities. Simultaneously, it uses a missing mask to learn the conditional distribution of unobserved modalities and features, enabling flexible imputation, integration, and downstream tasks in multimodal dataset through an end-to-end approach.

- Subtype-GAN [11] models complex multi-omics data by learning and outputting low-dimensional latent factors that conform to a prior distribution from diverse omics data with different distributions. These factors can be directly fed into FCNN to diagnose the cancer subtype of the corresponding samples.

We implemented kNN and RF classifiers using the sklearn [12] library. As for the remaining methods, we directly utilized shared code.

In addition, to demonstrate the cross-dataset knowledge transfer capability of CancerSD, apart from equipping well-performing subtype diagnostic methods with the pretraining strategy as baselines (MOMA [4]-PT and DCP [8]-PT), we also selected two advanced meta-learning-based knowledge transfer frameworks, including:

- QSFormer [13] addresses the challenge of achieving consistent representation and efficient metric learning between the support set and query set by incorporating a global query-support sample transformer branch and a local patch transformer learning branch. Additionally, it introduces a cross-scale interactive feature extractor as a backbone module to extract and fuse different scale CNN features. Finally, through end-to-end training of various modules, QSFormer learns knowledge from different datasets and applies it to the target dataset.

- DeepBDC [14] is a few-shot classification method based on the deep Brownian distance covariance, whose central idea is to learn sample representations by measuring the discrepancy between joint characteristic functions of embedded features and product of the marginals. DeepBDC is a decoupled and highly modularized network that can be instantiated in various few-shot classification frameworks.

Section B. Further analyses of diagnostic performance for cancer subtyping in the standard supervised learning setting

To delve deeper into the strengths and weaknesses of different subtype diagnosis methods, using the gastric cancer dataset (TCGA-STAD) as an example, we further analyzed their diagnostic performance presented in Table B in S1 Text.

Deep learning methods, including MI-based and iMI-based methods, usually offer apparent advantages and demonstrate greater potential for analyzing multi-omics data than traditional machine learning methods. This superiority stems from the enhanced feature representation capabilities of deep learning methods, allowing them to capture intricate connections and differences among different omics. In contrast, traditional machine learning methods, such as k-Nearest Neighbor classifier (kNN) and Random Forest Classifier (RFC), treat different omics as independent types of features, thus failing to exploit the benefits of multi-omics integration.

iMI-based methods are generally more effective than MI-based ones. CancerSD outperforms the best MI-based method, MOGONET, with Accuracy increased by 6.5%, AUROC by 5.5%, Precision by 6.9%, and F1 Score by 6.9%. The superior performance of iMI-based methods can be attributed to their ability to handle incomplete data, a prevalent fact overlooked by MI-based methods. Taking MOGONET as an example, an adjacent graph based on patient similarity needs to be built before the graph convolution operation. However, incomplete data can result in a significant number of noisy edges in the built similarity network, which dramatically compromises the performance of MOGONET.

Among the methods employing contrastive learning strategies (i.e., FactorCL, VICReg, and CancerSD), CancerSD significantly outperforms the other two in the cancer subtype diagnosis task. We attributed this to two main reasons. First, the presence of missing omics data introduces substantial noise, disrupting the feature space and leading to incomplete or even incorrect matching of positive and negative samples constructed by FactorCL and VICReg. This makes it challenging for these methods to capture the true similarity and difference patterns between samples. Second, the full utilization of multi-omics data is essential for the accurate diagnosis

of cancer subtypes. CancerSD constructs contrastive learning tasks to capture cross-omics consistency while designing masking-and-reconstruction tasks to maintain omics-specific information. Through the low-rank multimodal fusion strategy, CancerSD effectively balances consistency and specificity, and explores interactions between different omics, resulting in more accurate cancer subtype diagnosis. In contrast, VICReg prioritizes multi-omics consistency over omics-specific information, which limits its capability to fully leverage multi-omics data.

The comparison results highlight the importance of exploring the cooperation among different omics. CancerSD significantly surpasses all other iMI-based methods in most performance metrics. For instance, compared with DCP, CancerSD improves Accuracy by 4.1%, Precision by 4.2%, and F1 Score by 4.4% on the STAD dataset. This is because such these iMI-based methods focus more on inter-omics consistency and even assume that embedded representations of different omics should be identical. In the case of scVAEIT and Subtype-GAN, they attempt to leverage distributional information to project different omics data into a shared feature space, emphasizing the proximity of embeddings from different omics of the same sample within this space. As such, they overlook the exploration of specific information of multi-omics data. In contrast, CancerSD recovers the missing omics and employs a tensor fusion network to integrate multi-omics, exploring the inter-omics cooperation without compromising omics-specific information. Meanwhile, CancerSD extracts inter-omics consistency information by aligning various augmented views of the same samples. In this way, CancerSD ensures the consistency among different omics and explores their cooperation, thus improving subtype diagnostic performance under scenarios of incomplete multi-omics data.

In summary, from traditional machine learning methods to MI-based approaches, and further to iMI-based ones, as the aspects considered continue to expand, the performance of the models also improves consistently. This observation underscores the importance of detailed modeling of real-world problems for advancing the practical application of deep learning in clinical settings.

Section C. The performance of diagnostic models under scenarios of non-random omics data missingness

In clinical scenarios, factors such as cost and equipment limitations often lead to consistent missingness in certain omics data for patients. In other words, the omics missingness is non-random. To further assess the effectiveness of CancerSD in addressing incomplete multi-omics data, we extended our experiments, with the results presented in Tables E-G in S1 Text. Specifically, we first selected samples with complete data from the datasets and then masked certain omics types in several samples with probabilities of $\{25\%, 50\%, 75\%, 90\%\}$. We then evaluated CancerSD's performance in subtype diagnosis and missing omics imputation. Taking the case of missing mRNA data within the STAD dataset as an example, we analyzed the outcomes of the aforementioned experiments.

As shown in Table G in S1 Text, CancerSD consistently makes the top performance across all the missing rates. Overall, the performance of various tested methods exhibits a step-like distribution at each missing rate. Incomplete Multi-omics Integration (iMI)-based methods generally outperform those based on Multi-omics Integration (MI), while traditional machine learning approaches struggle to handle omics data missingness. For instance, the F1 Score for KNN dropped by over 20% (calculated by $(0.706 - 0.558)/0.706$) as the missing rate increases from 25% to 90%. In contrast, the fluctuation in the F1 Score for iMI-based methods remains around 5% under the same conditions. Additionally, it can be observed that the diagnostic performance of almost all methods presents a decreasing trend as the missing rate increases. An exception is KNN, which exhibits a slight performance improvement when the missing rate rises from 50% to 75% (a similar pattern can be observed in Tables E-F in S1 Text). This phenomenon could be attributed to KNN's sensitivity to distance metrics. Specifically, within a certain missing rate range, samples in the feature space may roughly cluster into two groups: one primarily consisting of samples with complete omics data, and the other of those with incomplete data. Consequently, samples undergoing diagnosed are likely grouped into one of these clusters based on their omics data integrity. As a result, KNN performs diagnosis within the cluster, leading to a diminished impact of omics missingness and a rebound in overall diagnostic performance.

Furthermore, by comparing the results in Table G in S1 Text (missingness occurs in mRNA) with those in Table M in S1 Text (partial multi-omics integration), we found that different methods exhibit varying levels of tolerance to missing omics data. Specifically, when the missing rate for mRNA approaches 50%, the diagnostic performance of traditional machine learning and MI-based methods is comparable to their performance when only methylation and miRNA profiles are used. However, as the missing rate further increases, the substantial missingness in mRNA data becomes an encumbrance for these two types of algorithm, adversely affecting multi-omics integration. For iMI-based methods, this critical threshold generally exceeds 75%. In other words, even when more than 75% of samples lacking mRNA data, iMI-based methods can still effectively extract valuable information from the remaining data to facilitate cancer subtype diagnosis.

In a word, CancerSD can reliably handle scenarios of non-random omics data absence. Even when certain omics type is severely missing, CancerSD is still able to effectively integrate the remaining incomplete multi-omics data to make accurate cancer subtype diagnosis.

Section D. Evaluation for the robustness of CancerSD

Besides superior diagnostic performance, the robustness of the model is also a crucial evaluation perspective. In our initial experimental setup, we performed ten completely random splits of the training and testing sets, along with random initialization of model parameters. As shown in Tables B and C in S1 Text and subsequent experimental results, the diagnostic performance of CancerSD remained relatively stable. To further investigate CancerSD's robustness to parameter initialization, we extended the experiments by fixing the dataset split and randomly initializing the network parameters. Specifically, for each fold in a ten-fold validation, we randomly initialized CancerSD ten times and reported its performance, where each fold represents a unique random split of the dataset. As illustrated in S3 Fig, while performance fluctuations are observed across different initializations, they are confined to a narrow range. Moreover, within each fold, CancerSD consistently maintains stable performance with random initialization. These findings suggest that CancerSD exhibits considerable robustness to both the dataset variations and network parameter initialization, indicating its adaptability to complex clinical scenarios.

Section E. Exploration for the optimal architecture of CancerSD backbone

To investigate the necessity of each module of CancerSD Backbone ($\mathrm{CancerSD_b}$) for effective cancer subtype diagnosis, we conducted extensive ablation studies, where four variations are considered: (i) CancerSD-FC utilizes a FCNN to integrate multi-omics; (ii) CancerSD-w/oGen removes omics-specific generators; (iii) CancerSD-w/oCon removes the patient representation augmenting module; (iv) CancerSD-w/oPrj discards the projector from data representation module.

As shown in Table H in S1 Text, CancerSD consistently outperforms all its variants in cancer subtype diagnosis tasks, with an Accuracy of 88.7%, AUROC of 96.0%, Precision of 88.9%, and F1 Score of 88.0%. Specifically, compared to CancerSD-FC, CancerSD demonstrates superior performance with a 3.9% increase in Accuracy, 3.3% in Precision, and 3.5% in F1 Score. This is because CancerSD-FC simply concatenates multiple omics data, which fails to capture deeper correlations among different omics. In contrast, CancerSD employs a tensor-based approach to integrate multi-omics data, which enables the exploration of intricate cooperation among different omics while also retaining the original omics-specific features. Therefore, CancerSD makes a more accurate and reliable subtype diagnosis.

Then, the imputation of the missing omics also contributes to improving subtype diagnosis, while the removal of omics-specific generators leads to a considerable decline in subtyping performance. This is because the absence of omics disrupts sample distributions, making it challenging to identify samples of different subtypes and complicating the optimization process for CancerSD.

To obtain more informative patient representations, we constructed contrastive learning tasks to optimize the patient feature encoder further. The performance decrease observed in CancerSD-w/oCon and CancerSD-w/oPrj highlights the importance of contrastive learning in improving sample representation quality. Specifi-

cally, CancerSD-w/oCon exhibits a decrease in Accuracy by 4.3%, AUROC by 1.6%, Precision by 4.0%, and F1 Score by 4.3%. This is because the instance-level contrastive loss encourages the encoder to explore consistency between different omics, supporting the learning of more discriminative representations by misaligning augmented views of different patients. Consequently, the enhanced patient encoder improves subsequent missing omics imputation and cancer subtype diagnosis processes. Moreover, CancerSD outperforms CancerSD-w/oPrj with increased Accuracy by 7.8%, AUROC by 3.4%, Precision by 7.8%, and F1 Score by 8.3%, indicating that the projector can significantly enhance the sample representation quality. We conjectured that the dimensionality reduction applied by the projector may lead to information loss, resulting in low-quality representations. Unfortunately, these representations make the contrastive learning tasks more challenging. In response to the difficulties arising from this information loss, the patient feature encoder in CancerSD tends to enrich and maintain more meaningful and discriminative information in patient representations. As a result, CancerSD demonstrates superior subtype diagnosis performance compared to CancerSD-w/oPrj.

In summary, the comprehensive evaluation of $\mathrm{CancerSD_b}$ and its variants underscores the synergistic contribution of all components to make superior cancer subtype diagnosis performance. The results also affirm the effectiveness of the proposed CancerSD model in leveraging incomplete multi-omics data for accurate cancer subtype diagnosis.

Section F. Selection of data augmentation operations is crucial for enhancing the patient feature encoder

In our work, we applied different omics-level masking strategies to the multi-omics features of patients, generating diverse augmented views. However, the choice of data augmentation operations is not one-size-fits-all and should be tailored according to the specific downstream tasks [15]. Therefore, to systematically study the effect of data augmentation operations, we considered several typical augmentations, including:

- identity mapping, which does not perform any processing on the patient features, namely $augment(\boldsymbol{x}_i) = \boldsymbol{x}_i$.
- omics-level masking, which randomly masks out some omics for each patient while retaining at least one type of omics data.
- molecule-level masking, which randomly masks out a given proportion of molecular characteristics for each patient.
- Gaussian blur, which introduces Gaussian noise to the multi-omics data of patients.
- mix-up, which mixes features of patients within the batch. Specifically, the features of the i-th patient can be represented as $\boldsymbol{x}_i = (1 - \gamma)\boldsymbol{x}_i + \gamma\boldsymbol{x}_j, i \neq j$, where $\boldsymbol{x}_j$ refers to other patient features within the same batch as $\boldsymbol{x}_i$.
- random scaling, where the features of each patient are multiplied by a scale factor.

We compared the F1 Score of CancerSD that utilizes different combinations of augmentation operations in gastric cancer subtype diagnosis tasks and the results are illustrated in S4a Fig. It can be observed that CancerSD makes the top performance when both augmentation operations involve the omics-level masking strategy. Meanwhile, the combination of omics-level masking with other operations also significantly improves the diagnostic performance of CancerSD compared to alternative augmentation compositions. Additionally, it is evident that the heatmap shown in Figure S10 is asymmetric. This is because we did not strictly ensure the symmetrical status of the two data augmentation branches, but force the auxiliary branch to align with the main one. In fact, we could observe that in most scenarios, this setup does not significantly impact the diagnostic performance of CancerSD.

We believed that there are two main reasons for the superior diagnostic performance made based on the combination of different omics-level masking strategies. On the one hand, the effectiveness of the patient feature encoder, trained based on omics-level masking strategies, lies in its enhanced capability to extract meaningful

information from patient features. Alternative augmentation operations, including molecule-level masking, Gaussian blur, mix-up, and random scaling, essentially introduce noise to patient features while retaining most of the original information in the representations. As shown in S4b Fig, we assessed the similarity between patient features after undergoing various data augmentation operations and the original features. Evidently, operations other than the omics-level masking exhibit higher similarity, suggesting that they preserve more of the original information during the transformation of patient features. Thus, the encoder may only need to extract shallow-level features to align different augmented views of patients, meeting the designed contrastive learning tasks. In contrast, the omics-level masking operation masks out at least a single or even multiple omics of patients each time, resulting in a more demanding alignment. In such cases, the encoder must capture intrinsic information from patient features to reach the alignment of different augmented views of the same patient.

On the other hand, the omics-level masking also synergizes effectively with the imputation of missing omics. To maintain the imputation capabilities of the generators, we designed the masking-and-reconstruction tasks, which are precisely in line with the principles of omics-level masking. Consequently, in comparison to other augmentations, the omics-level masking strategy aligns more harmoniously with CancerSD in the context of cancer subtype diagnosis using incomplete multi-omics data.

Section G. Performance of CancerSD under different hyper-parameters

To evaluate the parameter sensitivity of the CancerSD backbone network ($\mathrm{CancerSD_b}$), we compared the performance of our model under different hyper-parameters. For $\mathrm{CancerSD_b}$, one important parameter is the temperature parameter τ, which addresses the issue of hard negative samples [16]. In the context of CancerSD, we defined the negative sample as augmented views derived from different patients within the same batch, and the parameter τ is utilized to adjust the distribution of samples in the representation space. Specifically, we expected that patient representations embedded by the encoder can have a more uniform distribution while representations of patients with the same cancer subtype are close together, which requires striking a balance between uniformity and proximity of patient representations. Therefore, we chose different values of τ for experiments to explore its influence.

From S5a Fig, we can observe that, except for AUROC, the performance of CancerSD shows a trend of being higher in the midrange of τ values and lower on the sides. This is because the contrastive loss gradually concentrates on high-similarity regions as the value of τ decreases [16], and patients with the same cancer subtype generally exhibit higher similarity. In other words, when τ becomes small, the loss tends to penalize similar samples, increasing their distance in the representation space, even if they may belong to the same subtype, potentially leading to a decline in diagnostic performance. With the increase of τ, the loss penalizes samples more uniformly, making the similarity between samples smoother. This means that the differences between samples are smaller, which is not conducive to accurate cancer subtype diagnosis. In light of these insights, keeping the value of τ in a reasonable range is crucial for improving the diagnostic performance of CancerSD.

To integrate multi-omics data, we employ the tensor outer product operation and utilize tensor decomposition to simplify the integration process. Within this context, R is an important parameter that determines the number of low-rank tensors obtained by decomposing the transformation tensor $\mathbf{W}$. To evaluate the impact of different settings of R for CancerSD, we measure the change in performance on the STAD dataset while varying the values of R. As illustrated in S5b Fig, the overall performance of CancerSD gets its optimum value when R is assigned a value of 60. When R is too small, the approximate representation of $\mathbf{W}$ risks losing information inherent in the original transformation tensor, engendering a decrease in the effectiveness of multi-omics integration, thereby limiting the representation learning capability of CancerSD. As the increase of R, the complexity of the model correspondingly escalates. When R is too big, CancerSD becomes challenging to optimize, resulting in a decline in model performance. Therefore, striking a balance between the representation learning capability and complexity of the model is crucial for CancerSD to make the best cancer subtype diagnosis performance.

In addition, the training process of the CancerSD backbone involves the coordinated optimization of three loss terms, including instance-level contrastive loss, missing omics generation loss, and subtype diagnosis loss. To balance these three different losses, we have assigned two trade-off parameters, λ_1 and λ_2, which are both set to 1 by default in practice. Although CancerSD with fixed values of these parameters demonstrates excellent performance, exploring the impact of these parameters and unlocking the full potential of CancerSD remains crucial. As depicted in S5c,d Figs, we changed the value of λ_1 and λ_2 in the range of $\{0.01, 0.1, 0.2, 0.5, 1, 10, 100\}$. From the results, one could observe that CancerSD is not sensitive to λ_2. Conversely, a good choice for λ_1 can significantly improve the performance, and the optimal result is obtained when $\lambda_1 = 1$. While increasing λ_1 from 1 to 100, the performance of CancerSD experiences a significant decline. The right panel of S5e Fig provides a clearer illustration of this tendency (taking F1 Score as an example). When λ_1 increases from 1 to 100, the F1 Score of CancerSD sharply declines, whereas in other cases, the change in the F1 Score is not significant. This decline can be attributed to the fact that, when learning cross-omics consistency for patients, the instance-level contrastive loss treats different augmented views of the same patient as positive samples and those from different patients as negative ones. In other words, within the context of CancerSD, the contrastive loss considers each patient as a distinct category. With the growth of λ_1, the contrastive loss forces different patients to be more evenly distributed in the representation space (see S5f Fig). Consequently, the diagnosis loss is no longer able to effectively cluster patients of the same subtype together. This shift makes it challenging for CancerSD to distinguish between patient subtypes, resulting in a notable decline in diagnostic performance.

Section H. The performance of diagnostic models under different omics data types

To verify the importance of multi-omics integration in improving diagnosis and to evaluate CancerSD's capability in this regard, we granted it access to different combinations of omics data and reported its diagnostic performance. Table L in S1 Text demonstrates that CancerSD continuously improves its performance as more omics data are integrated. These results also indicate that CancerSD effectively captures the cooperation between different omics types, thereby enhancing the multi-omics fusion. To further underscore the advantages of CancerSD, we also compared it with several established methods. In this part of experiment, we selected methods that have demonstrated well performance in multi-omics cancer subtype diagnosis tasks, including RFC, MOMA, and APADC (each representing a distinct category of data fusion methods).

As shown in Table M in S1 Text, the diagnostic performance of all methods improves continuously by integrating more omics data. Models trained using all three omics (DNA methylation, miRNA expression, and mRNA expression) outperform those trained with combinations of two types of omics, which in turn perform better than corresponding models utilizing only a single omics modality. These findings underscore the significant benefits of integrating multiple omics data for more accurate subtype diagnosis. When constrained to use only a single omics modality, CancerSD does not stand out among other deep learning-based methods. In such cases, these methods essentially degenerate into simple classifiers, and slight differences in performance may be attributed to aspects such as variations in the omics encoder architecture or the design of auxiliary tasks. However, when multi-omics integration is applied, CancerSD demonstrates its potential and advantages. Through elaborately designed contrastive learning tasks, CancerSD adeptly captures cross-omics consistency. Meanwhile, it incorporates masking-and-reconstruction tasks to maintain omics-specific information as much as possible. By adequately utilizing and balancing both omics-shared and omics-specific information, CancerSD effectively integrates multi-omics data, thereby greatly improving its cancer subtype diagnosis performance.

Section I. Effectiveness of CancerSD in imputation for missing omics data

Due to cost, legal and ethical concerns, it is impractical and even infeasible to collect all omics data from patients. In practice, only partial omics data from the same patient is available for diagnosis. CancerSD adopts an imputation strategy to mitigate the negative impact that arose from these cases and demonstrates superior subtype diagnostic performance across multiple challenging cancer datasets with incomplete data. To

further validate the authenticity of CancerSD in leveraging incomplete multi-omics data for subtype diagnosis, we assessed its imputation capability by masking and subsequently recovering omics data. Concretely, we first selected samples with complete-paired multi-omics data and randomly mask certain omics of some samples with a masking rate of 75%, indicating that 75% of samples have some omics masked, simulating multi-omics data with incomplete issues. Table N in S1 Text lists the overview of different datasets after masking. Next, we utilized CancerSD to impute these simulated missing data and compare these imputations with the original data, which serves as the ground truth. Relevant results on the STAD dataset are shown in S7 Fig, while those on lung cancer and breast cancer datasets are presented in S8 and S9 Figs. At each step, we applied t-SNE to the sample data for dimensionality reduction and visualization and present the results in S7a Fig. We could observe that the absence of omics data significantly distorts the real sample distribution, highlighting a key reason why iMI-based methods tend to outperform MI-based ones. However, even in the face of such unrecognizable sample distributions, CancerSD can reliably recover initial distributions. Moreover, the high cosine similarity between the original and imputed data (see S7b Fig) also underscores the effectiveness of CancerSD for imputing missing omics.

To further demonstrate the imputation capability of CancerSD, we focused on mRNA expression data as an illustrative example and compare the differentially expressed genes (DEGs) identified from real and imputed scenarios (as mentioned in Section 6 of the Supplementary Texts). First, we employed the **limma** R package [17] to identify DEGs from different scenarios. Next, we used the **clusterProfiler** R package [18] to perform Gene Ontology (GO) analysis on identified DEGs. As shown in S7c,d Figs, a strong consistency is exhibited between the enrichment patterns of DEGs obtained from CancerSD-imputed mRNA expression data and the actual scenario. The enrichment results demonstrate a remarkable similarity in the biological process (BP), cellular component (CC), and molecular function (MF) ontologies. This consistency indicates that CancerSD can accurately recover biologically meaningful expression values, affirming the authenticity and effectiveness of CancerSD in imputing missing omics data.

In addition to evaluating the imputation capability of CancerSD for random data missingness, we also conducted experiments where each omics modality was selectively masked at varying proportions. This allowed us to further assess the flexibility and effectiveness of CancerSD in handling different missing data patterns. The relevant results of these extended experiments are presented in S10-S12 Figs. Taking the results from mRNA expression profiles as an example, we analyzed the imputation effectiveness of CancerSD for missing omics data. When the missing rate is relatively moderate (e.g., below 50%), CancerSD effectively utilizes the available methylation and miRNA data to recover the missing mRNA values, as evidenced by the low Mean Absolute Error (MAE) and Root Mean Square Error (RMSE) values in S12a Fig and the high similarity scores in S12b Fig. Additionally, the first two subplots of S12d Fig show that the imputed mRNA data by CancerSD accurately preserves the sample distribution seen in the original data. As the missing rate continues to increase, however, the clustering results based on the imputed data showed a trend of gathering according to sample subtypes (as depicted in the last two subplots of S12d Fig). This occurs because, at higher missing rate, CancerSD prioritizes the subtype diagnosis loss (i.e., $\mathcal{L}_{diagnosis}$) over the omics generation task (i.e., $\mathcal{L}_{generation}$). As a result, the imputed omics data contains more cancer subtype patterns rather than the unobserved original information (most of which has already been masked under high missing rates). In short, CancerSD demonstrates an adaptive capability to handle omics data missingness, thereby maintaining accurate cancer subtype diagnosis while effectively recovering missing data in a manner that closely reflects the original information.

To more intuitively demonstrate CancerSD's imputation capability, we compared it against several representative imputation strategies, including two statistical imputation algorithms (i.e., Zero and Mean [19]), two traditional machine learning imputation approaches (i.e., KNNI [20], MissFI [21]), and three deep learning imputation methods (i.e., GAIN [22], OTI [23], TDM [24]. Following previous work [25], we used MAE and RMSE to evaluate the accuracy of different methods in the imputation tasks. As shown in Tables U and V in S1 Text, Zero imputation consistently performs the worst across all experimental settings, establishing a lower bound for imputation performance. In contrast, CancerSD makes superior (or at least comparable) imputation

performance to its competitors across most scenarios. We believe its advantages primarily stem from two key aspects. First, CancerSD designs an imputation module based on auto-encoder architecture, which does not bind with any specific data distributions, thereby providing greater flexibility and adaptability. Conversely, distribution-alignment methods like OTI and TDM often operate under the assumption that different batches of data originate from the same distribution. However, due to the high heterogeneity of tumors [26], the omics data of different cancer subtypes may exhibit significant distribution difference, with notable variations even within the same subtype. These distribution difference hinders the further performance improvement of distribution-alignment methods.

Second, to fully leverage multi-omics data, CancerSD defines contrastive learning tasks and masking-and-reconstruction tasks. The former captures cross-omics consistency, while the latter minimizes the loss of omics-specific information. Most existing imputation methods focus primarily on individual molecular features, neglecting omics-level interactions, which results in their inferior performance in imputing missing cancer omics data. Although recent incomplete multi-view integration methods [8, 9, 27] emphasize exploring intra- and inter-view relationships and recovering missing data, they are inclined to adopt the strategy that only recovers task-relevant cross-view shared information. Consequently, we did not directly compare these methods (DCP [8] and APADC [9]) in terms of imputation quality. Instead, we focused on comparing the cancer subtype diagnosis performance of CancerSD with these methods (see Tables B and C in S1 Text).

We also observed that, as the missing rate rises, the imputation accuracy of all methods (as measured by RMSE and MAE) declines consistently. This is because a higher proportion of missing values results in less observable and usable information, diminishing the effectiveness of imputation algorithms. When the extent of missing omics is moderate, the imputation performance across different types of methods manifests a step-like distribution. In other words, deep learning methods generally outperform shallow machine learning ones, which surpass statistical approaches. When a certain omics modality experiences severely missingness, all methods (with the exception of Zero imputation) tend to perform at a similar level. In some cases, even statistical methods (Mean imputation in particular) may outperform some of their deep learning counterparts. We speculate that this occurs because severe data missingness significantly obscures the true distribution, making it difficult for distribution-fitting-based methods like GAIN to model the data effectively. Meanwhile, Mean imputation maintains the overall average level of the data without introducing additional variability, thereby reducing the impact of imputation on the overall data structure and allowing it to perform more robustly under extreme conditions of missing data.

In addition, we also studied the time consumption of aforementioned methods, as depicted in S13 Fig. We can observe from the figure that, (i) the time cost for a given imputation method remains stable when imputing different omics data at varying missing rates; (ii) the time overhead for Mean and Zero imputation is significantly shorter than that of other methods; (iii) MissFI consistently exhibits the longest execution time across all scenarios, due to the computational complexity of tree-based imputation methods over high-dimensional datasets, which necessitate building a predictive model for each incomplete feature; (iv) the average time cost of GAIN, OTI, TDM, and CancerSD remains at a similar level across different scenarios. To sum up, while simple statistical methods like Zero and Mean offer a clear advantage in terms of time efficiency, their limitations in imputation accuracy make them less suitable for handling complex data. In contrast, deep learning methods and certain traditional machine leaning approaches strike a better balance between efficiency and performance. Particularly in scenarios where high accuracy is required, the time cost associated with these methods is acceptable given the accuracy.

Section J. Summary of cancer subtype diagnosis results in lung cancer and breast cancer datasets

Although we conducted extensive experiments and in-depth analyses on the STAD dataset, CancerSD is not exclusively effective on gastric cancer. It can also be applied to subtype diagnosis tasks in other types of cancer. To demonstrate the versatility of our model, we conducted relevant experiments on datasets associated with lung cancer (LUAD, LUSC, and CPTAC) and breast cancer (BRCA). Among them, considering that the LUAD and LUSC datasets each predominantly represent a distinct subtype of lung cancer, we integrated them

into a unified lung cancer dataset, denoted as ADSC. An overview of these datasets is provided in Table A in S1 Text.

First, we assessed the effectiveness of CancerSD in subtype diagnosis on lung and breast cancer. It is worth mentioning that, as illustrated in S6 Fig, the original features of lung cancer samples exhibit good discriminative characteristics, with samples of the same subtype tending to cluster together. Therefore, to showcase the capabilities of different methods better, we employed a few-shot setting to increase the difficulty of lung cancer subtype diagnosis tasks. Specifically, we sampled only ten samples from each subtype for the training of the tested models. For breast cancer subtype diagnosis, we adhered to the canonical random 80/20 dataset split, where 80% of the samples were utilized for training and 20% for testing. As shown in Tables B and C in S1 Text, it is evident that traditional machine learning, MI-based, and iMI-based methods exhibit a stair-step improvement in performance for lung cancer subtype diagnosis, with our CancerSD making the optimal performance. This finding is in line with the results obtained on the gastric cancer dataset (STAD). It is noteworthy that MOGONET performs poorly in the few-shot subtype diagnosis task on the CPTAC dataset, aligning closely with KNN. This can be attributed to MOGONET's dependence on constructing a sample similarity network for graph convolution operations, where the initial distribution of samples significantly impacts its performance. This issue becomes more pronounced in the context of few-shot conditions, as MOGONET can only generate a similarity network with a noticeable bias, severely constraining its diagnostic capabilities. In addition, we could observe that iMI-based methods are marginally superior to MI-based ones for breast cancer subtype diagnosis. This phenomenon might be primarily attributed to the fact that the problem of incomplete data in the BRCA dataset is not severe (11 / 892, samples with incomplete data / all samples), limiting iMI-based methods to harness their potential fully. Interestingly, even though most samples in the BRCA dataset possess completely paired multi-omics data, we still found a distinct separation into two clusters among the samples. Further analysis of this result may provide new insights into breast cancer.

Then, we investigated the importance of different omics in the subtype diagnosis of lung cancer and breast cancer. For these analyses, we exclusively utilized samples with completely paired omics data and maintain consistency with previous experimental setups across different cancer datasets. Specifically, in the lung cancer dataset (ADSC), we allocated ten samples from each subtype for training, with the remaining samples used for testing. On the other hand, in the breast cancer dataset (BRCA), the training and testing sets are split in an 80/20 ratio. From Table L in S1 Text, we observed results similar to those in the gastric cancer dataset (STAD). In general, the performance of subtype diagnosis improves steadily with the integration of more omics types. However, the importance of various omics may vary across different cancers when only a single omics type is allowed for model training. In the gastric cancer dataset, CancerSD trained with mRNA expression data exhibits optimal performance. In ADSC, despite possessing the lowest dimensionality, miRNA features demonstrate the superior discriminative capability for lung cancer subtypes. In contrast, methylation features fail to effectively differentiate lung cancer subtypes, indicating potential similarities in DNA methylation patterns between the two subtypes (lung adenocarcinoma and lung squamous cell carcinoma) of lung cancer. Nevertheless, the inclusion of methylation features also modestly enhances diagnostic performance. In BRCA, mRNA expression profile emerges as the most critical omics for subtype diagnosis. Given that the PAM50 classification system [28] used in our experiments is constructed based on mRNA expression profiles, mRNA features indeed should excel in distinguishing different breast cancer subtypes, with other omics types primarily serving as supplementary information. In addition, experimental results under different training strategies (single-omics independent learning and multi-omics joint learning) on lung cancer and breast cancer datasets further validate conclusions obtained from the gastric cancer dataset, underscoring the effectiveness of our CancerSD in integrating multi-omics data. Besides its notable multi-omics integration capability, CancerSD also showcases outstanding performance in imputing missing omics data. Following the previous settings, we conducted the same experiments in the lung and breast cancer datasets, which randomly mask completely paired data to simulate omics data missingness and subsequently recover them. As illustrated in S8 and S9 Figs, CancerSD demonstrates the capability of recovering biologically meaningful expressions, even in cases of substantial omics data missingness (see Table N in S1 Text).

Finally, we validated the capabilities of the meta learner of CancerSD (CancerSD_m) through knowledge transfer tasks defined between the ADSC and CPTAC datasets. Specifically, ADSC is designated as the meta-training set, from which we sampled N-way K-shot tasks for optimizing our CancerSD. Each task comprises a support set $\mathcal{S}$ and a query set $\mathcal{Q}$, both including K samples for each of the N subtypes. Meanwhile, CPTAC serves as the meta-testing set, which is split into a training set containing one N-way K-shot task for fine-tuning and a testing set for diagnostic performance evaluation. Subsequently, we swapped the roles of the ADSC and CPTAC datasets for a more comprehensive evaluation and presented the results in Table T in S1 Text. Unlike the experiments on the gastric cancer dataset, which involve knowledge transfer only from multi-omics to single omics or from single omics to single omics, experiments on the lung cancer dataset encompass knowledge transfer from multi-omics to multi-omics. Regardless of how the knowledge transfer tasks are defined, CancerSD consistently shows superior performance, attributed to the generalization capability of its backbone network (CancerSD_b) and the capability of CancerSD_m on extracting domain-specific knowledge. Moreover, by comparing the performance of MOMA, DCP, and CancerSD in Tables C and T in S1 Text, we observed that due to disparities (i.e., sample distributions) among different datasets, directly transferring knowledge from external datasets may fail to optimize diagnostic models on the target dataset. These results further highlight the importance of purposefully learning knowledge from external datasets, an area where CancerSD excels.

References

[1] Breiman L. Random forests. Machine Learning. 2001;45:5–32. https://doi.org/10.1023/A:1010933404324

[2] Ma B, Meng F, Yan G, Yan H, Chai B, Song F. Diagnostic classification of cancers using extreme gradient boosting algorithm and multi-omics data. Computers in Biology and Medicine. 2020;121:103761. https://doi.org/10.1016/j.compbiomed.2020.103761 PMID:32339094

[3] Wang T, Shao W, Huang Z, Tang H, Zhang J, Ding Z, et al. MOGONET integrates multi-omics data using graph convolutional networks allowing patient classification and biomarker identification. Nature Communications. 2021;12(1):3445. https://doi.org/10.1038/s41467-021-23774-w PMID:34103512

[4] Moon S, Lee H. MOMA: a multi-task attention learning algorithm for multi-omics data interpretation and classification. Bioinformatics. 2022;38(8):2287–2296. https://doi.org/10.1093/bioinformatics/btac080 PMID:35157023

[5] Argelaguet R, Arnol D, Bredikhin D, Deloro Y, Velten B, Marioni JC, et al. MOFA+: a statistical framework for comprehensive integration of multi-modal single-cell data. Genome Biology. 2020;21:1–17. https://doi.org/10.1186/s13059-020-02015-1 PMID:32393329

[6] Liang PP, Deng Z, Ma MQ, Zou JY, Morency LP, Salakhutdinov R. Factorized contrastive learning: Going beyond multi-view redundancy. Advances in Neural Information Processing Systems. 2024;36.

[7] Bardes A, Ponce J, Lecun Y. VICReg: Variance-Invariance-Covariance Regularization For Self-Supervised Learning. In: ICLR 2022-International Conference on Learning Representations; 2022.

[8] Lin Y, Gou Y, Liu X, Bai J, Lv J, Peng X. Dual contrastive prediction for incomplete multi-view representation learning. IEEE Transactions on Pattern Analysis and Machine Intelligence. 2022;45(4):4447–4461. https://doi.org/10.1109/tpami.2022.3197238 PMID:35939466

[9] Xu J, Li C, Peng L, Ren Y, Shi X, Shen HT, et al. Adaptive feature projection with distribution alignment for deep incomplete multi-view clustering. IEEE Transactions on Image Processing. 2023;32:1354–1366. https://doi.org/10.1109/tip.2023.3243521 PMID:37022865

[10] Du JH, Cai Z, Roeder K. Robust probabilistic modeling for single-cell multimodal mosaic integration and imputation via scVAEIT. Proceedings of the National Academy of Sciences. 2022;119(49):e2214414119. https://doi.org/10.1073/pnas.2214414119 PMID:36459654

[11] Yang H, Chen R, Li D, Wang Z. Subtype-GAN: a deep learning approach for integrative cancer subtyping of multi-omics data. Bioinformatics. 2021;37(16):2231–2237. https://doi.org/10.1093/bioinformatics/btab109 PMID:33599254

[12] Pedregosa F, Varoquaux G, Gramfort A, Michel V, Thirion B, Grisel O, et al. Scikit-learn: Machine learning in Python. The Journal of Machine Learning Research. 2011;12:2825–2830. https://doi/10.5555/1953048.2078195

[13] Wang X, Wang X, Jiang B, Luo B. Few-Shot Learning Meets Transformer: Unified Query-Support Transformers for Few-Shot Classification. IEEE Transactions on Circuits and Systems for Video Technology. 2023;33:7789–7802. https://doi.org/10.1109/TCSVT.2023.3282777

[14] Xie J, Long F, Lv J, Wang Q, Li P. Joint distribution matters: Deep brownian distance covariance for few-shot classification. In: Proceedings of the IEEE/CVF Conference on Computer Vision and Pattern Recognition; 2022. p. 7972–7981.

[15] Tian Y, Sun C, Poole B, Krishnan D, Schmid C, Isola P. What makes for good views for contrastive learning? Advances in Neural Information Processing Systems. 2020;33:6827–6839.

[16] Wang F, Liu H. Understanding the behaviour of contrastive loss. In: Proceedings of the IEEE/CVF Conference on Computer Vision and Pattern Recognition; 2021. p. 2495–2504.

[17] Ritchie ME, Phipson B, Wu D, Hu Y, Law CW, Shi W, et al. limma powers differential expression analyses for RNA-sequencing and microarray studies. Nucleic Acids Research. 2015;43(7):e47–e47. https://doi.org/10.1093/nar/gkv007 PMID:25605792

[18] Yu G, Wang LG, Han Y, He QY. clusterProfiler: an R package for comparing biological themes among gene clusters. Omics: a journal of integrative biology. 2012;16(5):284–287. https://doi.org/10.1089/omi.2011.0118 PMID:22455463

[19] Farhangfar A, Kurgan LA, Pedrycz W. A novel framework for imputation of missing values in databases. IEEE Transactions on Systems, Man, and Cybernetics-Part A: Systems and Humans. 2007;37(5):692–709. https://doi.org/10.1109/TSMCA.2007.902631

[20] Troyanskaya O, Cantor M, Sherlock G, Brown P, Hastie T, Tibshirani R, et al. Missing value estimation methods for DNA microarrays. Bioinformatics. 2001;17(6):520–525. https://doi.org/10.1093/bioinformatics/17.6.520 PMID:11395428

[21] Stekhoven DJ, Bühlmann P. MissForest—non-parametric missing value imputation for mixed-type data. Bioinformatics. 2012;28(1):112–118. https://doi.org/10.1093/bioinformatics/btr597 PMID:22039212

[22] Yoon J, Jordon J, Schaar M. Gain: Missing data imputation using generative adversarial nets. In: International Conference on Machine Learning. PMLR; 2018. p. 5689–5698.

[23] Muzellec B, Josse J, Boyer C, Cuturi M. Missing data imputation using optimal transport. In: International Conference on Machine Learning. PMLR; 2020. p. 7130–7140.

[24] Zhao H, Sun K, Dezfouli A, Bonilla EV. Transformed distribution matching for missing value imputation. In: International Conference on Machine Learning. PMLR; 2023. p. 42159–42186.

[25] Miao X, Wu Y, Chen L, Gao Y, Yin J. An experimental survey of missing data imputation algorithms. IEEE Transactions on Knowledge and Data Engineering. 2022;35(7):6630–6650. https://doi.org/10.1109/TKDE.2022.3186498

[26] Hausser J, Alon U. Tumour heterogeneity and the evolutionary trade-offs of cancer. Nature Reviews Cancer. 2020;20(4):247–257. https://doi.org/10.1038/s41568-020-0241-6 PMID:32094544

[27] Zhou L, Du G, Lü K, Wang L, Du J. A Survey and an Empirical Evaluation of Multi-view Clustering Approaches. ACM Computing Surveys. 2024;56(7):1–38. https://doi.org/10.1145/3645108

[28] Parker JS, Mullins M, Cheang MC, Leung S, Voduc D, Vickery T, et al. Supervised risk predictor of breast cancer based on intrinsic subtypes. Journal of Clinical Oncology. 2009;27(8):1160. https://doi.org/10.1200/jco.2008.18.1370 PMID:19204204
